# Systemic immunosuppression promotes survival and integration of subretinally implanted human ESC-derived photoreceptor precursors in dogs

**DOI:** 10.1016/j.stemcr.2022.06.009

**Published:** 2022-07-28

**Authors:** Ana Ripolles-Garcia, Natalia Dolgova, M. Joseph Phillips, Svetlana Savina, Allison L. Ludwig, Sara A. Stuedemann, Uchenna Nlebedum, John H. Wolfe, Oliver A. Garden, Arvydas Maminishkis, Juan Amaral, Kapil Bharti, David M. Gamm, Gustavo D. Aguirre, William A. Beltran

**Affiliations:** 1 Division of Experimental Retinal Therapies, Department of Clinical Sciences and Advanced Medicine, School of Veterinary Medicine, University of Pennsylvania, Philadelphia, PA 19104, USA; 2 Waisman Center, University of Wisconsin-Madison, Madison, WI 53705, USA; 3 McPherson Eye Research Institute, University of Wisconsin-Madison, Madison, WI 53705, USA; 4 Department of Ophthalmology and Visual Sciences, University of Wisconsin-Madison, Madison, WI 53705, USA; 5 Walter Flato Goodman Center for Comparative Medical Genetics, School of Veterinary Medicine, University of Pennsylvania, Philadelphia, PA 19104, USA; 6 Children’s Hospital of Philadelphia, Philadelphia, PA 19104, USA; 7 Section on Epithelial and Retinal Physiology and Disease, National Eye Institute, NIH, Bethesda, MD 20892, USA; 8 Office of Scientific Director, National Eye Institute, NIH, Bethesda, MD 20892, USA; 9 Unit on Ocular and Stem Cell Translational Research, National Eye Institute, NIH, Bethesda, MD 20892, USA

**Keywords:** photoreceptor precursor, hESC-PRPCs, retinal organoid, retinal degeneration, canine models, fluorescence live imaging, immunosuppression

## Abstract

Regenerative therapies aimed at replacing photoreceptors are a promising approach for the treatment of otherwise incurable causes of blindness. However, such therapies still face significant hurdles, including the need to improve subretinal delivery and long-term survival rate of transplanted cells, and promote sufficient integration into the host retina. Here, we successfully delivered *in vitro*-derived human photoreceptor precursor cells (PRPCs; also known as immature photoreceptors) to the subretinal space of seven normal and three rcd1/*PDE6B* mutant dogs with advanced inherited retinal degeneration. Notably, while these xenografts were rejected in dogs that were not immunosuppressed, transplants in most dogs receiving systemic immunosuppression survived up to 3–5 months postinjection. Moreover, differentiation of donor PRPCs into photoreceptors with synaptic pedicle-like structures that established contact with second-order neurons was enhanced in rcd1/*PDE6B* mutant dogs. Together, our findings set the stage for evaluating functional vision restoration following photoreceptor replacement in canine models of inherited retinal degeneration.

## Highlights

• *In vivo* multimodal imaging identified early signs of transplant rejection
• Systemic immunosuppression was necessary for long-term survival of xenotransplants
• Human donor photoreceptors integrated and differentiated mostly into M/L cones
• A degenerating retinal environment enhances structural synaptogenesis in PRPCs

## Graphical abstract

**Figure:**
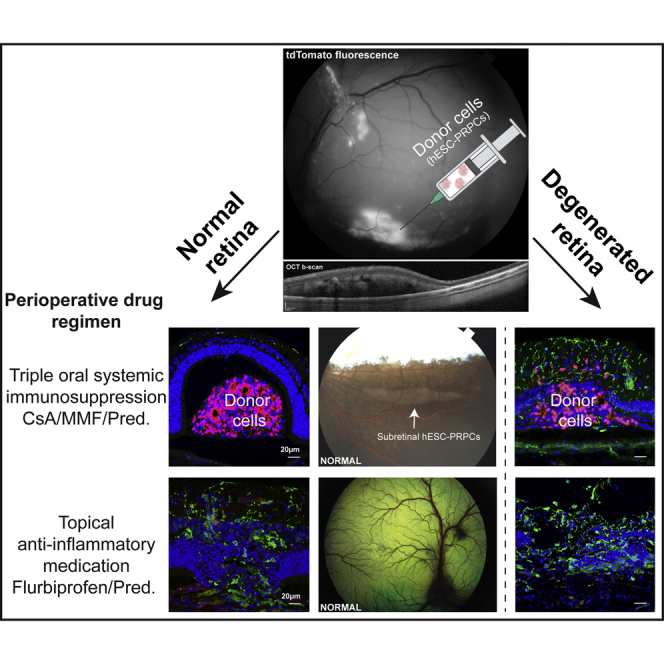

## Teaser

In this article, Beltran and colleagues described the subretinal delivery, immunosuppression protocol, and longitudinal multimodal imaging of transplanted PSC-PRPCs in normal and *PDE6B* mutant dogs. Immunosuppression was required for long-term (5 months) xenotransplant survival and integration. Degenerating retinas were more receptive to PRPC integration, where these differentiated into photoreceptors, developed synaptic pedicle-like structures, and established contact with second-order neurons.

## Introduction

Mammalian photoreceptors, the light-sensing outer retinal cells, lack self-regenerative capacity, and their degeneration in inherited retinal disease (IRD) is a major cause of blindness. However, while photoreceptors are lost in human IRDs and the dry form of age-related macular degeneration (AMD), the inner retinal structure is retained, even in advanced disease (Aghaizu et al., 2017). As such, regenerative therapies aimed at replacing photoreceptors and establishing functional synapses with the remaining viable inner retinal neurons is a promising approach for restoring vision recovery in otherwise blind patients.

Human embryonic stem cells (hESCs) or induced pluripotent stem cell (iPSC)-derived photoreceptor precursor cells (PRPCs) that develop into retinal organoids (ROs) are a promising source for regenerative experimental therapies to restore photoreceptor function (Ludwig and Gamm, 2021). Cell replacement studies in rodents have shown restoration of visual function using either ESC- or iPSC-PRPCs (Ribeiro et al., 2021; Tu et al., 2019), and several clinical trials in humans are exploring the use of allogenic and autologous cell therapies for photoreceptor replacement (Wang et al., 2020). However, exogenous photoreceptor cell replacement therapies face critical challenges (Ludwig and Gamm, 2021), including the need to (1) successfully deliver large numbers of cells subretinally to cover a large area, (2) ensure long-term photoreceptor survival rate by preventing immune rejection, and (3) promote sufficient integration into the host retina to recover visual function (Singh et al., 2018).

The dog offers unique advantages for advancing experimental retinal therapies (Ludwig and Gamm, 2021). Multiple models of dogs with naturally occurring mutations in some of the genes responsible for several forms of human IRDs are available and exhibit similar biological, pathological, and functional deficits as those reported for the human disease (Bunel et al., 2019). In addition, the size of the canine eye allows development and optimization of surgical and therapeutic approaches that can then be used in patients, and outcomes can be monitored using instruments commonly used in human clinics.

Here, we compared the ability of hPRPCs to survive and integrate in normal and in rcd1/*PDE6B* mutant dogs, an IRD model that undergoes early-onset progressive retinal degeneration primarily affecting rods. These mutant dogs undergo an acute phase of rods loss at 4–6 weeks of age that is followed by a progressive degeneration of rods and cones over the course of several months (Genini et al., 2013). Using a subretinal injector that was modified to accommodate hPRPC aggregates derived from ROs, we successfully delivered fluorescently tagged hPRPCs into the canine subretinal space (SRS) using a simple surgical procedure. A non-invasive multimodal imaging approach was used to assess cell viability and distribution and also enabled longitudinal analysis of individual cell clusters. A systemic immunosuppressive (IS) protocol of oral prednisolone, cyclosporine A (CsA), and mycophenolate mofetil (MMF) preserved-long term survival of hPRPC-derived cells. Donor cells in normal dogs receiving IS remained mostly in the SRS and attempted to create structural synapses but were largely unsuccessful, likely because of their inability to penetrate the intact outer limiting membrane (OLM). In contrast, the OLM of mutant dogs receiving IS was focally disrupted, and hPRPCs had extended axons and structural formations that resembled synaptic terminals, suggesting that disease-related retinal changes enhance the potential of hPRPCs to form synapses when properly integrated and polarized within the retina.

## Results

### Multimodal retinal imaging enables non-invasive monitoring of hESC-PRPCs injected into the SRS of normal and degenerated canine retinas

To accommodate the larger size of hPRPC aggregates obtained from ROs, we performed the subretinal delivery of donor cells using a custom-modified subretinal injector with a 31G or 33G cannula. With this approach, a prior vitrectomy was not required, and the cells could be delivered by bolus injection controlled by manual pressure. Human ESC-PRPCs derived from one of two photoreceptor reporter lines (either WA09 *CRX*-tdTomato or WA09 *NRL*-EGFP) (Phillips et al., 2018a, 2018b) were successfully injected into the SRS of normal (12 eyes) and rcd1/*PDE6B* mutant (6 eyes) dogs without surgical complications. Details are listed in Table 1. In all dogs, some of the cell suspension was seen backflowing into the vitreous at the time of the bolus injection before the retinotomy was formed and the bleb began to expand. In non-vitrectomized eyes (n = 16), retinal blebs reattached within 24–48 h, while in the two vitrectomized eyes, 6–7 days were needed for full reattachment.

**Table 1:** Details of cell transplantation and monitoring time points in all study animals

	Dog ID (sex, age in weeks at injection)	Eye	Genotype (disease)	Cell suspension type	Estimation of cells delivered in the SRS	Donor cells age at transplant/euthanasia (days)	1 Week^a^	4 Weeks^a^	8 Weeks^a^	12 Weeks^a^	16 Weeks^a^	22 Weeks^a^	32 Weeks^a^
Normal IS	SP-8 (M, 45)	OS	Wild type (normal)	Aggr CRX^/tdTomato+^	4 million in 150 μL	D107/D188	LI	LI	LI	LI/IHC			
	AS2-427 (F, 161)	OD	Wild type (normal)	Aggr CRX^/tdTomato+^	2.7 million in 100 μL	D151/D233	LI	LI	LI	LI/IHC			
		OS		Aggr CRX^/tdTomato+^	2.7 million in 100 μL	D151/D233	LI	LI	LI	LI/IHC			
	N339 (M, 19)	OD	Wild type (normal)	Aggr NRL^+/EGFP^	4 million in 100 μL	D130/D215	LI	LI	LI	LI/IHC			
		OS		Aggr NRL^+/EGFP^	4 million in 100 μL	D130/D215	LI	LI	LI	LI/IHC			
	2294 (F, 80)	OD	Wild type (normal)	Diss CRX^/tdTomato+^	4 million in 150 μL	D133/D277	LI	LI	LI	LI	LI	LI/IHC	
		OS		Aggr CRX^/tdTomato+^	4 million in 150 μL	D133/D277	LI	LI	LI	LI	LI	LI/IHC	
	SSA-1 (M, 131)	OD	Wild type (normal)	Aggr CRX^/tdTomato+^	2 million in 150 μL	D109/D120	LI/IHC						
		OS		Aggr CRX^/tdTomato+^	4 million in 150 μL	D110/D120	LI/IHC						
	SSA-3 (M, 76)	OD	Wild type (normal)	Aggr CRX^/tdTomato+^	4 million in 150 μL	D133/D354	LI	LI	LI	LI	LI		
		OS		Aggr CRX^/tdTomato+^	4 million in 150 μL	D133/D354	LI	LI	LI	LI	LI		
Normal No-IS	SSA-3 (M, 76)	OD	Wild type (normal)									LI	LI/IHC
		OS										LI	LI/IHC
	SP-10 (M, 45)	OD	Wild type (normal)	Aggr CRX^/tdTomato+^	4 million in 150 μL	D104/D185	LI	LI	LI	LI/IHC			
Mutant IS	2299 (M, 29)	OD	*Pde6β*^*−/−*^ (rcd1)	Aggr CRX^/tdTomato+^	4 million in 100 μL	D125/D210	LI	LI	LI	LI/IHC			
		OS		Aggr CRX^/tdTomato+^	4 million in 100 μL	D125/D210	LI	LI	LI	LI/IHC			
	2307 (F, 29)	OD	*Pde6β*^*−/−*^ (rcd1)	Aggr CRX^/tdTomato+^	2 million in 100 μL	D125/D188	LI	LI	LI/IHC				
		OS		Aggr CRX^/tdTomato+^	2 million in 100 μL	D125/D188	LI	LI	LI/IHC				
Mutant No IS	2306 (F, 29)	OD	*Pde6β*^*−/−*^ (rcd1)	Aggr CRX^/tdTomato+^	4 million in 100 μL	D125/D210	LI	LI	LI	LI/IHC			
		OS		Aggr CRX^/tdTomato+^	2 million in 100 μL	D125/D210	LI	LI	LI	LI/IHC			

Aggr, aggregated; D, day; Diss, dissociated; F, female; ID, identification number; IHC, immunohistochemical assessment; IS, immunosuppression; LI, life imaging; M, male; OD, right eye; OS, left eye; SRS, subretinal space.

a Weeks posttransplantation ±1 week.

Donor cells were evaluated *in vivo*, at different time points, using a multimodal imaging approach that included color fundus photography, a custom-modified Topcon retinal camera to image tdTomato fluorescence, autofluorescence (AF) confocal scanning laser ophthalmoscopy (cSLO) to image EGFP, near-infrared (NIR) cSLO, spectral-domain optical coherence tomography (OCT), and *en face* OCT. Importantly, we were also able to longitudinally follow individual cell clusters and evaluate subclinical signs of transplant rejection.

**Figure 1:**
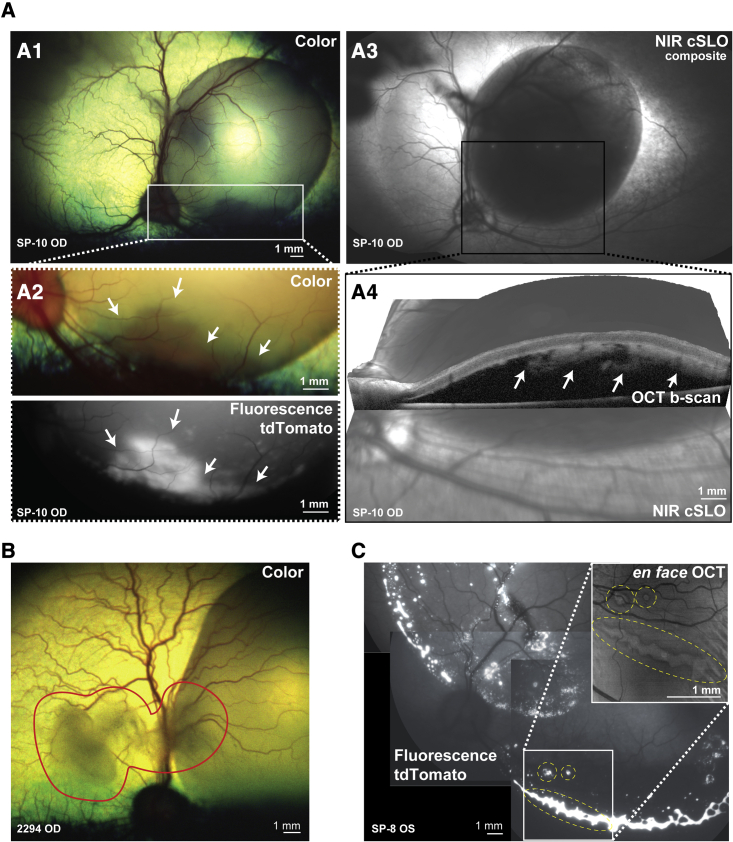
Multimodal retinal imaging following subretinal transplantation of hESC-CRX^+/tdTomato^-derived photoreceptor precursor cells (PRPCs) (A1) Immediate post-operative color fundus photograph (RetCam camera) showing the subretinal bleb. (A2) Color and fluorescent fundus photograph (modified Topcon retinal camera) showing a cluster of subretinally transplanted cells (white arrows) expressing tdTomato. (A3) NIR cSLO image (Spectralis HRA/OCT2) of the bleb. (A4) Three-dimensional view combining NIR cSLO and OCT b-scan confirming the presence of transplanted cells in the subretinal space. (B) Immediate post-operative color fundus photograph illustrating the presence of donor cells within the vitreous (area enclosed within red curved line). (C) Collage of fundus fluorescence photographs of tdTomato-expressing donor cells visualized 1 week post-injection along the border of the subretinally treated area. Inset: *en face* OCT image of the slab delimited by the photoreceptor inner and outer segments and the retinal pigment epithelium confirms the location of the donor cell cluster in the subretinal space. cSLO, confocal scanning laser ophthalmoscope; NIR, near infrared; OCT, optical coherence tomography; OD, right eye; OS, left eye.

Using this multimodal approach, we evaluated each recipient for the presence of tdTomato- or EGFP-positive cells in the surgically created bleb, confirming a successful injection (Figures 1A1 and 1A2). NIR cSLO and OCT b-scans were next used to verify the subretinal location of transplanted hPRPCs (Figures 1A3 and 1A4). The backflow of cells into the vitreous was confirmed and documented by fundus photography (Figure 1B). In all eyes, subretinally injected cells were mostly distributed in a major cluster, located in the inferior border of the bleb (Figure 1C), likely because of gravitational deposition as dogs were repositioned upright following surgery, acquiring the appearance of a subretinal pseudohypopyon when examined ophthalmoscopically (Figures 1A1 and 1A2). Additional smaller clusters of cells were seen throughout the bleb when using fluorescence retinal imaging and *en face* OCT (Figure 1C). Although the delivered cell dose was estimated prior to injection (Table 1), variation in the size of cell clusters prevented exact quantification of the number of subretinally delivered PRPCs in the SRS and vitreous.

### Systemic immunosuppression is necessary for long-term survival of xenotransplants in the canine SRS

A cohort of normal (n = 11 eyes) and rcd1/*PDE6B* mutant (n = 4 eyes) dogs received systemic IS and topical medication to prevent xenograft rejection (Figure S1). The IS regimen combined oral prednisolone, CsA, and MMF. CsA pharmacokinetic (PK) and pharmacodynamic (PD) analyses confirmed that overall, CsA levels were above the minimum recommended by the laboratory (Clinical Pharmacology Laboratory, Auburn University, AL, USA) (Figure S1C), and there was moderate interleukin-2 (IL-2) mRNA suppression (Figure S1D). This IS protocol was well tolerated in almost all the dogs and resulted in effective IS in seven out of eight animals. The one exception developed palpebral, interdigital, and oral papillomatous formations and viral plaques consistent with canine papillomavirus infection, likely facilitated by reduced immune competence (data not shown).

**Figure 2:**
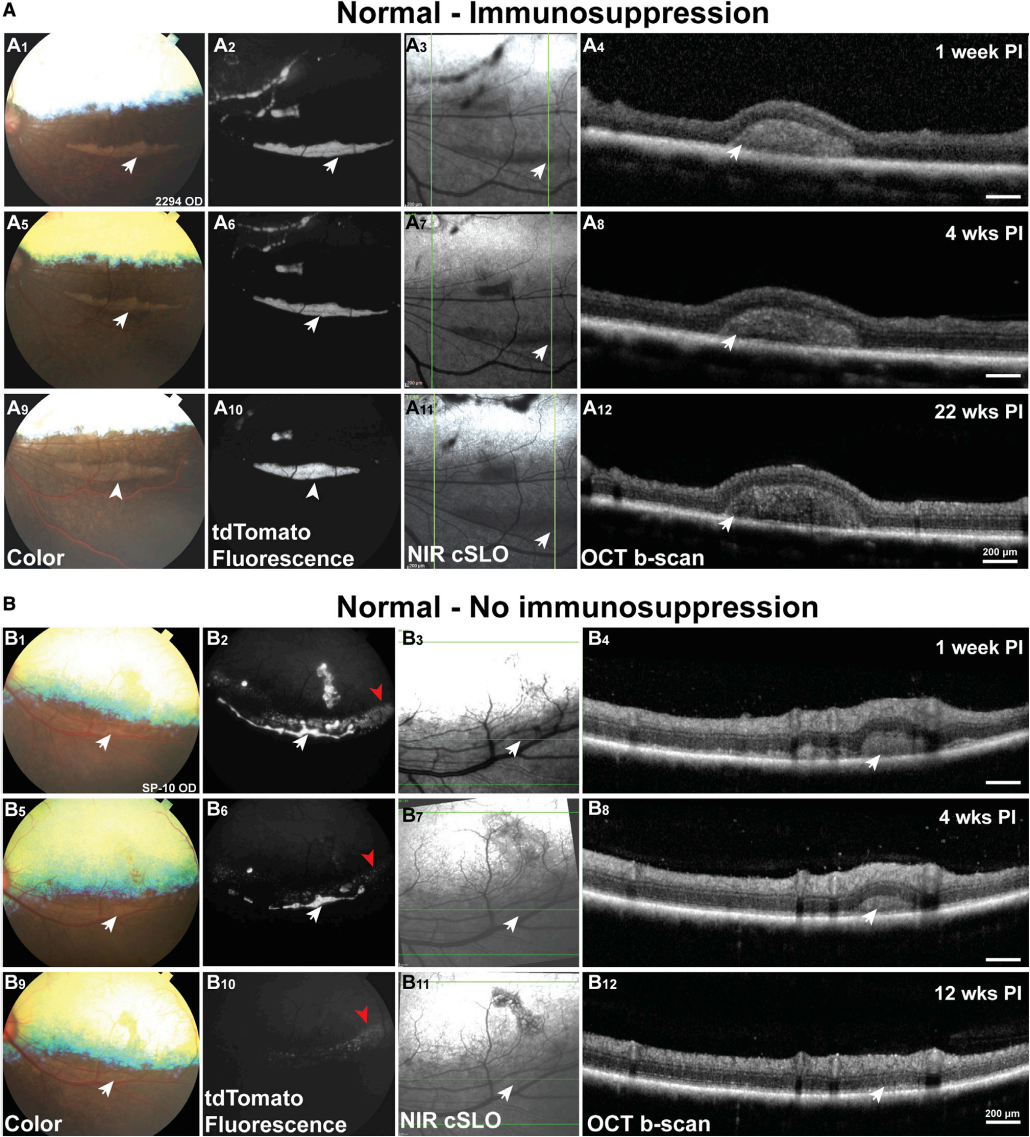
Longitudinal imaging of the subretinal cell mass in dogs with and without immunosuppression (IS) (A) Color (A1, A5, and A9), fluorescence (A2, A6, and A10), NIR cSLO (A3, A7, and A11), and OCT b-scan (A4, A8, and A12) retinal imaging acquired 1 (A1–A4), 4 (A5–A8), and 22 weeks (A9–A12) after subretinal cell delivery in one animal that was under systemic IS. (B) Color (B1, B5, and B9), fluorescence (B2, B6, and B10), NIR cSLO (B3, B7, and B11), and OCT b-scan (B4, B8, and B12) retinal imaging acquired 1 (B1–B4), 4 (B5–B8), and 12 weeks (B9–B12) after subretinal cell delivery in one animal that did not receive systemic IS treatment. White arrows point to the cell masses in the subretinal space. Red arrows point to low-intensity background autofluorescence, seen in only one animal not under IS, that was confirmed by histology to originate from hypertrophied host RPE cells filled with lipofuscin-like fluorescent material (data not shown).

Although the graft volume decreased between 3 days and 1 week postinjection (PI) in IS animals (Figures S2A1–S2A8), no further substantial cell loss was seen up to 22 weeks PI (Figures S2A9–S2A12). Most animals received aggregated cells (n = 17 eyes), and one animal received enzymatically dissociated cells in one eye. Notably, there was good hPRPC survival after the initial 1-week post-surgery loss in dogs under the triple oral IS protocol in both mutant and normal animals (Figure 2A). In contrast, transplanted cell survival was significantly impaired in both normal and mutant dogs not receiving IS treatment (Table S1), as revealed by a substantial and continuous decrease in fluorescence (Figures 2B2, 2B6, and 2B10) and in the size of the subretinal cell clusters (Figures 2B4, 2B8, and 2B12), that continued until the cells were no longer detectable (Figures 2B9–2B12).

Loss of PRPCs in non-IS animals was not accompanied by severe ocular signs of inflammation, but a mild vasculitis characterized ophthalmoscopically by vessel tortuosity and perivascular cuffing was observed as early as 1 week PI (Figure S3A). By OCT, generalized retinal swelling and a hyperreflective material in the vitreous was seen at 1 week PI, suggestive of transplant rejection (Figure S3B). These OCT features were also observed in one animal in which the IS regimen was intentionally halted (ID: SSA-3) 2 weeks after medication withdrawal. In this animal, there was an increase in the volume of the subretinal cell mass at this time (Table 2; Figures S2B5–S2B12) that was followed by a drastic decrease in both fluorescence and cell mass volume in the SRS by 10 weeks after IS withdrawal (Table 2; Figures S2B13–S2B16), possibly reflecting graft infiltration and clearance by inflammatory cells.

**Table 2:** Percentage of the cell mass volume compared with that at 1 week posttransplantation

Group	Dog ID	Eye	4 ± 1 week PI	8 ± 1 week PI	12 ± 1 week PI	22 ± 1 week PI	30 ± 1 week PI
Normal IS	SP-8	OS	−23	115	121		
	2294	OD	−24	−6	−9	15	
		OS	36	71	118	283	
	AS2-427	OD	70	115	241		
		OS	−9	97	102		
	N339	OD	−53	−36	285		
		OS	−43	−26	−53		
	SSA-3	OD	−43	13	58		
		OS	−19	42	243		
	Mean (SD)		−12 (40)	43 (60)	123 (116)	145 (190)	
Normal no IS	SSA-3	OD				718	−21
		OS				529	410
	SP-10	OD	61	46	20		
	Mean (SD)		61	46	20	623 (133)	195 (305)
Mutant IS	2299	OD	36	41	17		
		OS	19	−11	−17		
	2307	OD	−47	−35			
		OS	−5				
	Mean (SD)		1 (36)	−2 (39)	0.2 (24)		
Mutant no IS	2306	OD	−100	−100	−100		
		OS	−28	−45	−41		
	Mean (SD)		−64 (51)	−72 (39)	−71 (42)		

ID, identification number; IS, systemic immunosuppression; OD, right eye; OS, left eye; PI, postinjection.

Surprisingly, in the single animal injected bilaterally with WA09 NRL^+/EGFP^ hESCs (ID: N339), in which only rod precursors express EGFP, there were severe funduscopic signs of transplant rejection despite IS treatment (Figure S4), which occurred as the prednisolone regimen was tapered down according to the protocol (12 weeks PI). Notably, in this dog, the cell mass that reached the SRS was markedly larger than in other eyes (Figures S4A2–S4A5). By 12 weeks PI, clinical signs of vasculitis and vitreal haze were visible ophthalmoscopically (Figures S4A5, S4A7, and S4A9) and EGFP fluorescence was no longer detectable (Figure S4B2). OCT revealed retinal swelling with multifocal retinal detachments, pre-retinal vitreal condensation, and an increase in subretinal cell mass volume (Figure S4B4). The retinal inflammation was confirmed by immunohistochemistry (IHC) (Figure S4C).

### Longitudinal imaging of transplanted donor cells indicates different patterns of donor cell integration depending on host retinal status

Qualitative assessment of the cluster of donor cells that deposited in the ventral region of the bleb showed changes in shape over time that differed between normal and rcd1/*PDE6B* mutant dogs (Figure S5A). To further analyze these changes, we measured the area occupied by the main cell cluster and its mean height in all eyes using the HEYEX software (Figure S5B) to estimate its volume. In animals that received IS, two distinctive trends were seen based on the host’s retinal status. In normal animals, the cell mass area decreased (38% at 12 weeks PI), but its height increased over time (44% 12 weeks after transplantation) (Figures S5A1, S5A3, S5A5, and S5C1). In mutant animals, there were no changes to the area or height of the donor cell mass, likely reflecting less intercellular remodeling within the transplant (Figures S5A2, S5A4, S5A6, and S5C2).

In normal animals not treated with IS, there was an initial increase in height and volume with a decrease in area (59% at 12 weeks PI), possibly reflecting immune rejection and infiltration of inflammatory cells within the first 4 weeks PI (Figure S5C3). In two out of two eyes from the rcd1/*PDE6B* mutant dog that was not under IS, the graft area decreased progressively (60% at 12 weeks PI), indicating poor cell survival and clearance of donor cells from the SRS (Figure S5C4).

### Histological evaluation confirms that immunosuppression must be maintained to prevent xenotransplant rejection

**Figure 3:**
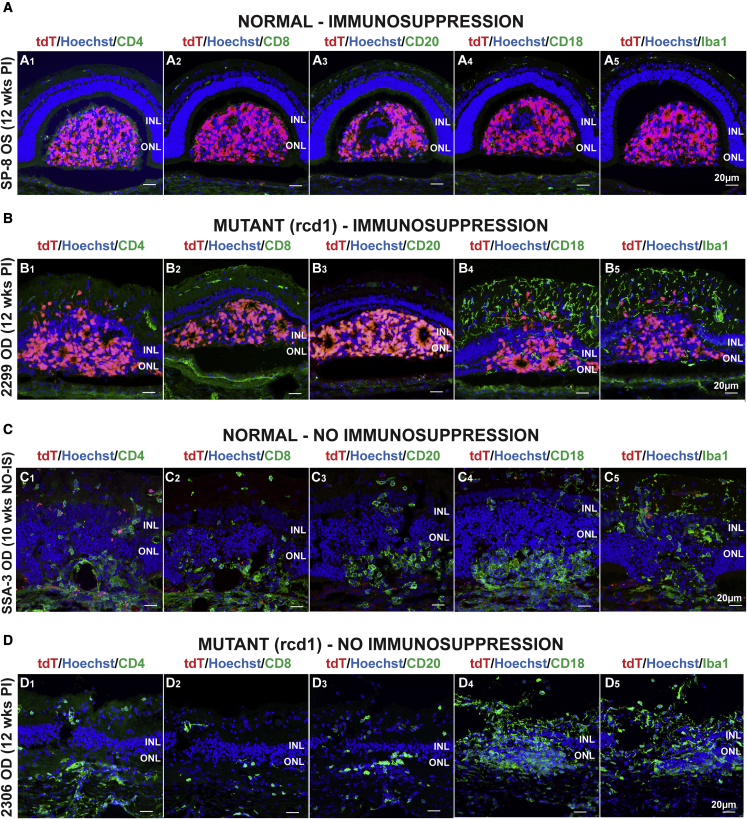
Immunohistochemical characterization of cell-mediated response toward transplanted cells in normal and mutant dogs under or without IS (A) Absence of immune cells and survival of transplanted donor tdTomato-positive cells in the subretinal space of a normal dog under IS (A1–A5). (B) Survival and migration of transplanted donor tdTomato-positive cells into the host retina of a mutant (rcd1/*PDE6B*) dog with no evidence of immune T (B1-2) or B (B3) cell infiltration into the graft. Rare macrophage (B4) and microglial (B5) cells were seen in the graft but were mostly found throughout the inner retina. (C) Graft rejection with infiltration of immune cells in a normal dog without IS. (D) Complete graft rejection with rare T and B cells and massive infiltration of macrophages and microglial cells in the retina of a mutant (rcd1/*PDE6B*) dog that was not under IS.

When evaluated histologically, the retinas of animals that received IS had no to very limited inflammatory cells infiltrating the transplant (Figures 3A and 3B), except for one normal animal injected with NRL^+/EGFP^ hESCs (Figure S4C). In this animal, perivascular inflammatory cells composed of a mixed inflammatory cell infiltrate confirmed the clinical diagnosis of retinal vasculitis (Figure S4C), and the absence of EGFP-positive donor cells in the SRS and host retina, coupled with severe disruption of retinal lamination, confirmed transplant rejection.

In both non-IS normal and mutant dogs, we found a mixed inflammatory infiltrate composed mostly of macrophages (CD18^+^; Figures 3C4 and 3D4) and resident microglia (Iba1^+^; Figures 3C5 and 3D5) and to a lesser extent by helper and cytotoxic T cells (CD4^+^ and CD8^+^, respectively; Figures 3C1, 3C2, 3D1, and 3D2) and B cells (CD20^+^; Figures 3C3 and 3D3), suggesting a robust activation of the innate immune response and to a lesser extent a cellular adaptive immune response. In addition, the number of donor cells (tdTomato- or EGFP- and Ku80-positive cells) was minimal to absent at this time, and there was an associated disruption of the normal host’s retinal architecture (Figures 3C and 3D).

In all cases, rcd1/*PDE6B* mutant dogs under IS (n = 4 eyes) exhibited a diffuse infiltration of microglia and blood-derived macrophages (Iba1^+^ and CD18^+^ cells, respectively; Figures 3B4 and 3B5) throughout the inner retina, in both treated and untreated areas (Figures S6A1, S6A2, S6A4, and S6A5). These findings were also observed in archival tissues from uninjected rcd1/*PDE6B* mutant dogs of 22, 26, and 39 weeks of age (Figures S6A3 and S6A6). Thus, we consider this cellular infiltration a normal feature of the neuroinflammation that is associated with the natural course of retinal degeneration rather than a consequence of the delivery of hESC-PRPCs.

Unexpectedly, several small, isolated clusters of PRPCs were detected in the ganglion cell layer (GCL) of two normal dogs that were under IS (Figures S6B1–S6B5). Because we did not see a subretinal cell mass nor structural retinal modification in this region, these cells were likely unintentionally delivered under the inner limiting membrane at the time of injection (Figures S6C1–S6C3, area enclosed within red dashed line). Indeed, OCT imaging performed immediately after transplantation showed a subgroup of cells deposited between the inner limiting membrane and the nerve fiber layer (NFL) in one of these animals (Figure S6C4, white arrow). Within the GCL, these cells triggered a moderate immune response dominated by blood-derived macrophages (CD18^+^) and microglia (Iba1^+^) (Figures S6B1–S6B5). In this same eye, PRPCs within the immune-privileged SRS did not trigger a similar inflammatory cell reaction (Figures S6B6–S6B10).

### Mature donor photoreceptors in the SRS were mostly M/L cones

**Figure 4:**
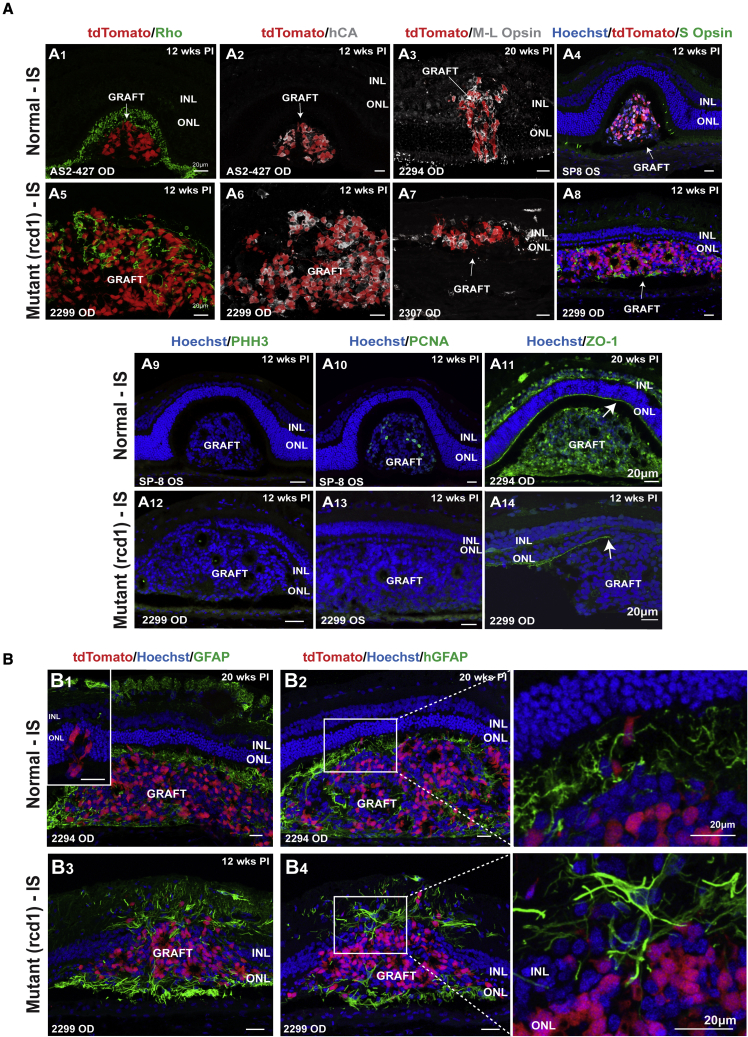
Characterization of the donor cell graft in the canine subretinal space (A) Transplanted tdTomato^+^ PRPCs mature to express rhodopsin (Rho; A1 and A5), human cone arrestin (hCA; A2 and A6), canine M/L opsin (A3 and A7), and S opsin (A4 and A8). This was seen in six of the eight normal and in all the mutant eyes (n = 4 eyes) under IS; representative images per group are illustrated. Rare cells positive for proliferating cell nuclear antigen (PCNA; A10 and A13) but negative for phospho-histone H3 (PPH3, A9 and A12) were noted in three out of eight normal eyes under IS, and integration into the host retina was seen at sites of OLM disruption stained with zonula occludens-1 (ZO-1) antibodies (A11 and A14 white arrows) only in mutant animals under IS. (B) Immunostaining with a glial fibrillary acid protein (GFAP) antibody (B1 and B3) that recognizes both canine and human GFAP, as well as with a human-specific GFAP (hGFAP) antibody (B2 and B4), illustrates the formation of a glial scar around the transplant in all the normal eyes, whereas this did not occur in mutant eyes.

We evaluated the fate of PRPCs by assessing the expression of markers of mature human and canine rods (rhodopsin [Rho]), mature human cones (human cone arrestin [hCA]), as well as canine and human cones (M/L opsin and S opsin). Rho expression suggested that human rods were present (Figures 4A1 and 4A5). However, we consistently saw that most donor photoreceptors were cones (Figures 4A2 and 4A6), particularly M/L cones (Figures 4A3 and A7) and, to a lesser extent, S cones (Figures 4A4 and 4A8).

To rule out any tumorigenic potential of the graft, we assessed proliferating cell nuclear antigen (PCNA), a marker of retinal proliferation, and DNA repair in dogs and phospho-histone H3 (PHH3), specific to cells undergoing mitosis. None of the donor cell clusters contained any PHH3-positive cells (Figures 4A9 and 4A12). Positive PCNA labeling within the donor cell mass was seen in only four normal dogs (with and without IS) and was limited to fewer than 10 cells per section inside the main cluster (Figures 4A10 and 4A13).

### Human PRPCs show anatomic integration into the canine retina and structural potential for synaptic connectivity

We next evaluated the integrity of the host OLM by staining for zonula occludens-1 (ZO-1), a tight junction protein present in the OLM. In normal retinas, the OLM was a thick, continuous structure (Figure 4A11, white arrow). On the contrary, in mutant animals, the OLM was focally disrupted and did not have a regular thickness (Figure 4A14, white arrow). This finding was confirmed in uninjected rcd1/*PDE6B* mutant retinas at 22, 26, and 39 weeks of age (data not shown).

Although donor PRPCs were originally thought to integrate into the recipient retina, recent reports in rodents indicate that donor-host cytoplasmic material exchange can also occur (Ortin-Martinez et al., 2017). To assess potential exchange of donor-host cytoplasmic material in dogs following transplantation of human PRPCs, we undertook IHC analysis in which the cell nuclei of donor human cells (Ku80^+^) was immunostained in parallel with the other markers of interest. Notably, we did not observe any evidence of cellular material exchange in any of the retinas at the different time points evaluated, because all the cells that expressed tdTomato or GFP also had Ku80-positive nuclei (Table S2).

**Figure 5:**
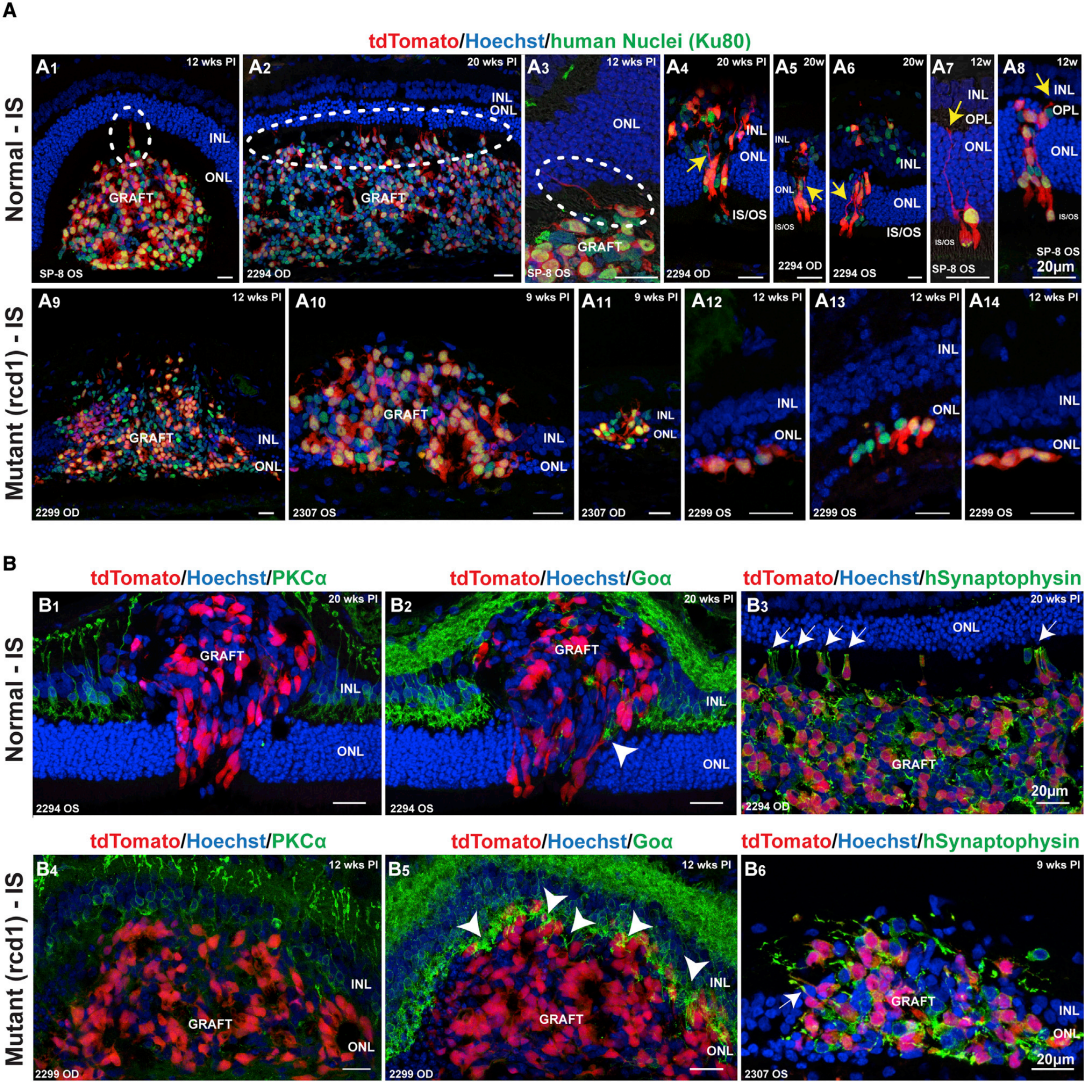
Donor cell integration in normal and mutant canine host retinas and development of synaptic structures (A) In three of eight eyes from normal animals under IS, some rare donor cells extended processes into the host retina from their subretinal location (A1–A3, white dashed oval). In these same eyes, smaller cell aggregates were able to translocate their nuclei into the donor ONL (A4–A8), with an elongated photoreceptor-like morphology, including inner segment formation and neurite extension toward the donor OPL (yellow arrows). In all the rcd1/*PDE6B* mutant animals under IS, there was increased integration of the donor cell cluster within the host retinal layers (A9–A11), with individual cells seen in the host ONL (A12–A14). (B) Rare anatomically integrated donor cells in three of eight eyes from normal animals under IS showed only minimal potential for structural synapses with few PKCα-positive rod bipolar cells (B1) and Goα-positive ON bipolar cells (B2, white arrowhead). Non-integrated donor photoreceptors that remained in the subretinal space extended neurites into the host retina ending in synaptophysin-positive pedicle-like structures that were unable to penetrate the host’s OLM (B3, white arrows). In all the rcd1/*PDE6B* mutant animals under IS, when the donor cell cluster was integrated into the host ONL, rod bipolar cells (B4) and ON bipolar cells (B5, white arrowheads) were seen extending dendrites toward the grafted cells. Donor photoreceptors extended axons into the host retina expressing synaptophysin (B6, white arrow).

In normal animals that received IS treatment, donor cells remained mostly in the SRS (Figures 5A1–5A3), with a few PRPCs from the main cluster sending cytoplasmic projections toward the outer nuclear layer (ONL) (Figures 5A1–5A3, white dashed circles). However, the somata of these PRPCs remained unable to penetrate the normal OLM. Such attempts to create structural synapses were seen as early as 12 and 20 weeks PI (but not at 2 weeks PI). In addition, sporadic smaller clusters of donor cells migrated into the ONL, outer plexiform layer (OPL), and inner nuclear layer (INL) in three out of eight normal retinas under IS (Figures 5A4–5A8; Video S1). Some of these cells exhibited photoreceptor morphology, with short outer segment (OS)-like structures and axon extension polarized toward the OPL (Figures 5A4–5A8, yellow arrows; Video S1). In the remaining five retinas, one animal (two eyes) evaluated at 2 weeks PI showed poor integration, another had a small graft and no migration (12 weeks PI), and another (two eyes) transplanted with NRL^+/EGFP^ cells showed signs of transplant rejection.

Video S1. 3D confocal imaging of donor human PRPCs integrated into a normal canine retina and adopting a cone-like morphology Two tdTomato-positive PRPCs expressing the human nuclear antigen (Ku80) have migrated their cell bodies into the host’s ONL. These cells show an elongated photoreceptor-like morphology that includes an inner segment, and an axon extending toward the host’s outer plexiform layer and ending with a pedicle-like structure. Images are shown with and without differential interference contrast (DIC)/Nomarski optics

Notably, a different pattern was seen in both eyes of the two mutant dogs with IS: in multiple areas of all four eyes, the main cell cluster was frequently seen translocated into the ONL, OPL, and INL (Figures 5A9–5A11). These integration events were observed in 0 of 10 sections evaluated in normal animals (n = 3 eyes) and between 4 of 10 and 10 of 10 sections in mutants (n = 3 eyes) (see Table S3). As with the normal retinas, smaller cell clusters were also able to migrate into the host ONL, OPL, and INL (Figures 5A11–5A14). Cells that migrated into the host ONL had extended axons and structural formations that resembled synaptic terminals. Host rod and cone ON bipolar cells extended Goα-positive dendrites toward donor cells within the ONL. This mainly occurred for PRPCs with cone-like morphology (Figures 5B2 and 5B5, white arrowheads). Occasionally protein kinase C alpha (PKCα)-positive rod bipolar cells also extended processes toward donor cells (Figures 5B1 and 5B4). In both mutant and normal retinas, the PRPCs consistently expressed the pre-synaptic protein human synaptophysin, suggesting their potential for forming synapses when properly integrated and polarized within the retina (Figures 5B3 and 5B6, white arrows).

### Survival and integration of the donor cells: role of donor Müller cells

Donor cells isolated from day 104 to 151 ROs used in this study were composed primarily of PRPCs but also contained retinal ganglion cells, horizontal cells, amacrine cells, bipolar cells, and Müller glia (Phillips et al., 2018b). Following injection, subretinal cell clusters from these donor cell populations included PRPCs (tdTomato^+^/Ku80^+^), as well as Müller cells (human glial fibrillary acid protein positive [GFAP^+^]) and bipolar cells (PKCα^+^/Ku80^+^ and Goα^+^/Ku80^+^). The donor Müller cells in normal animals under IS mainly surrounded the transplant, while endogenous canine GFAP expression was restricted to astrocytes within the NFL (Figures 4B1 and 4B2). While the glial cells within the donor tissue might promote cell survival, glial scarring around transplants in normal retinas has been observed (Aboualizadeh et al., 2020). Together, these findings suggest that formation of a glial scar encapsulating the graft likely impedes optimal cell migration and integration in animals with intact OLM. In support, smaller donor cell clusters that were not surrounded by GFAP-positive material more frequently integrated into the host retina (Figure 4B1, inset).

In rcd1/*PDE6B* mutant dogs, widespread mild GFAP upregulation in host Müller glial cells was consistent with ongoing neurodegeneration. However, in contrast with normal dogs, glial scars enclosing the graft were not observed at this stage (Figures 4B3 and 4B4). Moreover, reactive host Müller glia did not appear to prevent migration or anatomic integration of the grafted tissue in the mutant microenvironment. Interestingly, in mutant animals, donor Müller cells also extended processes and connected host and donor tissues (Figure 4B4), suggesting that they may serve as a conduit for donor cell migration or integration into the host retina.

### Validation of a new surgical approach to optimize delivery of cell suspensions to the SRS

Having observed in all dogs a variable amount of cells refluxing into the vitreous at the time of the bolus subretinal injection, we expanded this study to test a novel “five-step” subretinal delivery approach (Video S2; supplemental experimental procedures S1). Although the need for a three-port pars plana vitrectomy followed by removal of the hyaloid membrane extended the duration of the surgery, the preformation of a BSS subretinal bleb that was deflated before injecting the cell suspension drastically reduced the amount of cell suspension that refluxed into the vitreal cavity. This optimized approach provides a way of delivering to the SRS the intended dose of cell suspension and reduces the risk of inducing proliferative vitreoretinopathy.

Video S2. Five-step surgical procedure used to optimize in the canine eye the subretinal delivery of cell suspensions by avoiding reflux into the vitreal cavity

## Discussion

Transplantation of PSC-derived RPE cells for the treatment of AMD and Stargardt’s macular dystrophy has now moved into clinical trials (Wang et al., 2020). In contrast, progress with photoreceptor replacement still faces a number of challenges, including optimizing uniform delivery of a large number of donor cells, improving long-term survival of donor photoreceptors, promoting sufficient integration into the host retina, and establishing functional synaptogenesis to enable recovery of visual function (Ludwig and Gamm, 2021). In addition, the avoidance of immune rejection of allogeneic transplants needs further optimization before clinical trials can be initiated (Zhang et al., 2021). To address these issues, we have developed longitudinal multimodal imaging to evaluate the effect of a triple-drug IS regimen on cell survival and integration of hESC-PRPCs following surgical delivery to the SRS of both normal dogs and dogs with advanced IRD. This has enabled further evaluation of the impact of the host’s degenerating retinal environment on PRPC migration, differentiation, and structural integration.

### Surgical delivery of cells to the SRS

We successfully delivered cells in the canine SRS using a simple surgical procedure (bolus manual injection without prior vitrectomy). Notably, vitreal reflux is common when injecting cells in the SRS (Aboualizadeh et al., 2020) because a larger retinotomy is formed as a result of the need for a wider gauge subretinal canula for cell delivery. As suggested in previous work performed in non-human primates (NHPs), the cells remaining in the vitreous can potentially affect vision and post-operative retinal imaging (Aboualizadeh et al., 2020). Although such findings suggest that vitreal backflow of donor cells should be minimized to prevent formation of epiretinal membranes that can lead to secondary retinal detachment, the presence of refluxed cells in the vitreous of canine eyes in our studies was not associated with post-operative complications up to 22 weeks PI. However, to improve the translational value of using canine models to evaluate photoreceptor transplantation, we developed and validated a new five-step surgical delivery approach that considerably reduces vitreal reflux and thus increases the safety profile of this procedure.

Although the transvitreal approach for subretinal injection has been used in several human clinical trials of stem/progenitor cell transplantation (Wang et al., 2020), we found it was successful but suboptimal for hESC-PRPC delivery in the canine SRS. Specifically, we consistently observed an uneven distribution of cells within the SRS, thus limiting its potential therapeutic benefits. The heterogeneous distribution is most likely due to gravitational effects, because the animal’s head is positioned upright immediately after surgery (Aboualizadeh et al., 2020). In clinical trials, such effects could be mitigated by post-operative bedrest and/or administration of carbonic anhydrase inhibitors that accelerate subretinal fluid resorption (Wolfensberger, 1999). Alternatively, the use of biocompatible scaffolds or biomaterials that provide a structural matrix may optimize donor cell distribution and polarization (Lee et al., 2021).

### Multimodal imaging of donor cells and host retina is critical to monitoring graft survival and detecting early signs of transplant rejection

Visualization of fluorescent PRPCs in the live animal allowed us to differentiate them from host cells, thus improving on prior techniques in which non-fluorescent donor cells could be followed only by OCT or detected by histology after termination. Although this approach did not offer single-cell resolution such as that achieved with fluorescent adaptive optics scanning laser ophthalmoscopy (FAOSLO) (Aboualizadeh et al., 2020), this simple imaging technique still enabled longitudinal tracking of the same cell cluster to assess survival and integration into the host retina. In addition, we found that the use of *en face* OCT improved our ability to observe grafts in the SRS, further enhancing the detection of small clusters of donor cells that were not clearly seen on *en face* views with fundus photography or cSLO.

Consistent with previous studies (West et al., 2010), we observed two temporal patterns of donor cells loss: an early reduction in the number of grafted cells within the first week of transplantation that was independent of IS status and a delayed rejection of the graft, seen in those dogs not receiving IS. Although the cause of the initial cell loss is not known, it could potentially be driven by an early innate immune response (Kennelly et al., 2017), cell damage during processing of the ROs or surgical injection (Jager et al., 2016), as well as anoikis or apoptosis as a result of loss of cellular adhesion (Scruggs et al., 2019).

In the animals that were not under IS, there was a slight increase in graft volume in the normal dogs (20% at 12 weeks PI) and a marked decrease in the mutants (71% at 12 weeks PI). However, in all cases, this was accompanied by a drastic decrease in donor cells, as assessed by decreased donor cell fluorescence. In a previous study of RPE graft rejection in the SRS of NHP, extinction of donor cell fluorescence was a key finding indicating poor outcome, because a mononuclear inflammatory infiltrate in the SRS of these retinas could be mistaken for transplanted cells when imaging by OCT (McGill et al., 2018). Thus, multimodal retinal imaging that combines fundus photography (color and fluorescence), cSLO/OCT, and/or novel technologies such as FAOSLO provide the most accurate way of detecting early loss of donor cells (Aboualizadeh et al., 2020).

Interestingly, in normal dogs under IS, the graft volume in the SRS increased over time, but there was no evidence of inflammatory or proliferative cells. Reorganization of the graft, vacuolization, rosette formation, development of a glial barrier around the transplant (Aboualizadeh et al., 2020), or change in individual cell morphology/volume might have caused this increase.

Currently, there are no established clinical criteria to define transplant rejection in the SRS (Petrash et al., 2021), although some recognized signs of rejection are those associated with inflammation. These include hazy vitreous, vasculitis, retinal swelling, and retinal detachment (Petrash et al., 2021). Here we observed signs compatible with transplant rejection in animals that did not receive systemic IS, as well as in a single dog whose IS treatment was halted. The degree of clinical inflammation varied between animals, but retinal vasculitis, hazy vitreous, and retinal swelling were common in all the dogs with rejection of donor cells. These signs were first detected between 1 and 2–12 weeks posttransplantation, thus supporting the need for frequent monitoring of the treated retinas in the months that follow transplantation to detect potential early signs of rejection and provide an opportunity for adjusting the IS regimen.

### Systemic immunosuppression is required for survival and differentiation of xenotransplants

Prior studies in mice and rats showed that IS is required to prevent allograft rejection in the SRS (Seiler et al., 2014; Zhu et al., 2017), a site previously considered to be immune privileged (West et al., 2010). Immunosuppression is a common approach to manage a wide variety of immune-mediated and inflammatory diseases in dogs (Whitley and Day, 2011). To target both innate and adaptive immune responses, we combined oral prednisolone, CsA, and MMF, as well as topical anti-inflammatory medications. This protocol, which was initiated a week prior to transplantation and maintained throughout the study, was found to be both efficacious and well tolerated. The one exception was a dog that developed viral-induced papillomas, likely because its immunocompromised state rendered it susceptible to infection.

Consistent with prior studies in rodents and as previously reported in dogs (Xian and Huang, 2015), we found that systemic IS is critical for the survival of subretinally delivered hESC-PRPCs. Surprisingly, transplant rejection was still observed in one animal receiving systemic IS. This animal was injected with PRPCs where the rod precursors expressed EGFP and received a considerably larger transplant volume than achieved in other dogs because of a more efficient surgical delivery. Because EGFP is a known immunogen that can trigger an inflammatory response (Ansari et al., 2016), the increased EGFP expression in this dog may have triggered a more robust immune response when oral prednisolone was tapered down.

### No evidence of cytoplasmic material transfer following transplantation of human PRPCs into the canine SRS

Several groups have previously reported that donor cells transplanted into the SRS fail to integrate in the recipient murine retina but instead engage in a process of cytoplasmic material transfer of RNA and/or protein (including fluorescent reporter proteins) with the host photoreceptors (Ortin-Martinez et al., 2017). We critically evaluated whether a similar mechanism occurred in canine retinas but did not observe any evidence of cytoplasmic material transfer between donor cells and host photoreceptors. However, because previous studies suggest that cytoplasmic material exchange is less likely to occur in a xenograft scenario than with autologous or allogenic transplants (Gonzalez-Cordero et al., 2017), future studies will be needed to evaluate its impact on transplant of allogeneic hESC-derived PRPCs into patients.

### Grafted cells integrate more efficiently into degenerated retinas

The OLM, which is formed by tight junctions between photoreceptor inner segments and Müller glia (Omri et al., 2010), is a major barrier for migration of donor cells from the SRS into the host retina. Our results in normal dogs confirm findings in intact NHP and rodent retinas that show grafted cells persisting in the SRS in the absence of chemically, pharmacologically, or laser-induced ablation of the OLM integrity (Aboualizadeh et al., 2020; Pearson et al., 2010; West et al., 2008). However, consistent with enhanced donor cell integration by the progressive OLM disruption associated with photoreceptor degeneration (Waldron et al., 2018), we found that PRPC integration was enhanced in the rcd1/*PDE6B* mutant dog model.

Reactive gliosis and secondary retinal remodeling in IRDs are considered potential impediments to donor cell integration (Hippert et al., 2016). Although activation of Iba1^+^ microglia cells and GFAP immunoreactivity in canine Müller cells was seen throughout the retinas of degenerating rcd1/*PDE6B* mutant dogs, it did not prevent migration of donor cells. Although these dogs were transplanted at an advanced stage of degeneration (∼2 rows of nuclei left in the ONL), there was no evidence of a disease-associated host Müller glial seal in the SRS. However, because specific aspects of degeneration in the diseased retina may differentially impact integration, identifying non-invasive methods to assess the permissive state of a degenerated retina to cell migration prior to photoreceptor transplantation may be critical for optimal patient selection.

The donor cell suspension isolated from day 104 to 151 ROs was composed primarily of PRPCs but also contained other retinal cell populations, including Müller cells, which were detected using a human-specific GFAP antibody that did not cross-react with canine GFAP. Although these co-injected human Müller cells did not prevent PRPC migration in degenerating retinas, they formed a “mesh” of glial processes around the graft in normal retinas, likely contributing to graft retention in the SRS and morphological changes by OCT imaging. In support, rare events of PRPC migration and cone differentiation into the host ONL were seen in normal retinas, which in all cases involved single or few PRPCs that were not surrounded by donor Müller cells. This suggests that in intact retinas, the glial seal formed by the donor Müller cells around the transplanted graft may be as much or more of a barrier to cell migration as the presence of an intact OLM. The lack of formation of such a structure in the transplanted rcd1/*PDE6B* retinas and the loss of integrity of the OLM suggest that the diseased retina provides an enhanced microenvironment for PRPC graft integration and potentially differentiation.

This study now describes an optimized five-step surgical approach to improve delivery of the full intended dose of cells to the SRS, yet cell distribution throughout the treated area was still found to be limited to the ventral border of the bleb as a result of gravitational deposition. The use of biodegradable scaffolds (Jung et al., 2018; Lee et al., 2021) to implant PRPCs over a larger area may circumvent this limitation, improve radial orientation of the photoreceptor cells, and favor the establishment of xenosynapses that could provide functional recovery.

Although previous studies support the potential for PRPCs to establish structural synapses (Aboualizadeh et al., 2020), few studies have shown functional recovery after xenotransplantation of human cells in retinas with end-stage degeneration (Barnea-Cramer et al., 2016; Garita-Hernandez et al., 2019; Ribeiro et al., 2021). In the present study, enhanced integration of PRPCs within the degenerating retina was exemplified by development of pedicle-like structures, expression of the pre-synaptic protein synaptophysin, and establishment of contacts with host ON bipolar cells. These encouraging results now set the stage for functional evaluation of these xenosynapses in canine models of retinal degeneration to establish the translational potential of this therapeutic strategy.

## Experimental procedures

### Study animals

The dogs were part of a research colony kept at the University of Pennsylvania, Retinal Diseases Studies Facility. All procedures were carried out in strict accordance with the Association for Research in Vision and Ophthalmology (ARVO) Statement for the Use of Animals in Ophthalmic and Vision Research and approved by the Institutional Animal Care and Use Committee of the University of Pennsylvania (IACUC number: 803254).

A total of 10 dogs (6 males and 4 females; 5 months to 3 years of age) were used (Table 1), including 7 dogs (12 eyes) with normal retinas and 3 dogs (6 eyes) that were affected with a form of rod cone degeneration caused by a nonsense mutation in the *PDE6B* gene (rcd1/*PDE6B*). These dogs serve as a large-animal model of retinitis pigmentosa and by 29 weeks of age exhibit an advanced stage of retinal degeneration, defined by an ONL thickness (∼2 rows of nuclei) that is less than 75% of that of a normal dog at the same age (Genini et al., 2013).

### Preparation and transplantation of PRPCs

Three-dimensional (3D) ROs were generated from two established hESC reporter lines (WA09 *CRX*-tdTomato or WA09 *NRL*-EGFP; WiCell, Madison, WI, USA) (Phillips et al., 2018a, 2018b) following previously established protocols (Capowski et al., 2019) to produce fluorescently labeled PRPCs (WA09 *CRX*-tdTomato) or rod precursors (WA09 *NRL*-EGFP). As previously characterized (Capowski et al., 2019; Phillips et al., 2018a, 2018b), about 70% of the cells in the ROs are CRX-tdTomato PRPCs. Cell preparation and subretinal delivery details are fully described in supplemental experimental procedures S1.

### Pharmacological treatment

The animals were divided into two study groups: dogs that received triple anti-inflammatory/IS drugs (IS group; Figure S1A) and animals that were not under this medication (no-IS group). In those animals receiving the IS medical regimen, CsA (5–10 mg/kg twice a day; Atopica, Elanco US, Greenfield, IN, USA) and MMF (10 mg/kg twice a day; compounded at Wedgewood Village Pharmacy, Swedesboro, NJ, USA) were initiated 1 week before cell transplantation and given for the entire duration of the study. The dogs also received oral prednisolone (1 to 0.1 mg/kg once daily; Lannett Company, Philadelphia, PA, USA), initiated on the day of cell transplantation and progressively tapered down during a 12-week period PI. In those dogs that did not receive IS, no systemic anti-inflammatory or IS medication was given in the post-operative period (Figure S1B). In addition, the dogs received a perioperative regimen that differed in the IS and no-IS group (see supplemental experimental procedures S2).

Throughout the evaluation period, physical examinations and blood and urine collection were performed to assess whether the IS protocol was well tolerated by the dogs and to ensure that proper IS levels were achieved (see supplemental experimental procedures S2).

### *In vivo* longitudinal monitoring of transplanted PRPCs using multimodal retinal imaging

On a weekly basis, the animals underwent an ophthalmic examination that included biomicroscopic slit-lamp evaluation, intraocular pressure measurement, and observation of the transplant by indirect ophthalmoscopy. At the end of the evaluation, retinal photographs were acquired with a RetCam retinal camera.

At different time points (Table 1), the animals were anesthetized as previously described for cell delivery, and a detailed analysis of the grafted cells was performed by using retinal color and fluorescence photographs, cSLO (NIR and AF modes), spectral-domain OCT, and *en face* OCT. Initially, several images were acquired with a Topcon retinal camera (Topcon Medical Systems, Paramus, NJ, USA) that was custom modified to incorporate a set of excitation (FF01-531/40-25; Semrock, Rochester, NY, USA) and emission (FF01-586/20; Semrock, Rochester, NY, USA) filters that enable the visualization of tdTomato fluorescence. Once the donor cells were localized, a Spectralis HRA/OCT2 unit (Heidelberg Engineering, Heidelberg, Germany) was used to evaluate the donor cell changes by cSLO (55-degree lens), OCT, and *en face* OCT (30-degree lens). In the animal injected with NRL^+/EGFP^ hESCs, the Spectralis AF mode of the cSLO was used to identify the EGFP^+^ cells, followed by OCT and *en face* OCT evaluation. The retinal blood vessels were used as a landmark to compare the same region between the different imaging modalities used. In addition, the Spectralis unit follow-up function was used to ensure longitudinal evaluation of the same region. The OCT raster scans consisted of 30 × 25 degrees (10 × 10 degrees for *en face* OCT) volumetric scans containing 61 (512 for *en face* OCT) horizontal b-scans spaced every 122 μm (11 μm for *en face* OCT).

### *In vivo* quantification of the subretinal cell clusters over time

For further investigation of the transplanted cells features and evolution *in vivo*, the major cell cluster in each eye was longitudinally quantified. This was performed in all animals using the Spectralis designated software (HEYEX, Heidelberg Eye Explorer; Heidelberg Engineering, Franklin, MA, USA; see supplemental experimental procedures S3).

### Immunohistochemical evaluation

At the end of the study, the animals were humanely euthanized with an intravenous overdose of pentobarbital sodium and phenytoin sodium (Euthasol; Virbac, Westlake, TX, USA). Immediately following death confirmation, the eyes were enucleated, fixed, and processed as previously described (Beltran et al., 2017). The sections were stained with H&E and different IHC markers (Table S4) following protocols previously established (Beltran et al., 2017). Immunolabeled sections were examined by confocal microscopy (Leica TCS SP5; Leica Microsystems, Buffalo Grove, IL, USA), and digital images were acquired and processed using the Leica Application suite program. Additional details can be found in supplemental experimental procedures S4.

Immunohistochemical quantification of the integration events was performed in a subset of normal and mutant animals (see supplemental experimental procedures S5).

## Author contributions

A.R.-G. assisted with surgery and animal care, conducted all the *in vivo* imaging of transplanted cells in the animals, performed immunohistochemical examination, and wrote the original draft. J.H.W. and D.M.G. conceived the project, acquired funding, and reviewed the manuscript. N.D. acquired and analyzed confocal microscopy images. M.J.P. and A.L.L. produced and coordinated shipment of cells for transplantation and reviewed the manuscript. S.A.S. and U.N. produced cells for transplantation. S.S. performed cryo-sectioning and immunolabeling. O.A.G. provided guidance with the immunosuppression protocol and analyzed results. G.D.A. conceived the project, provided animal resources, and reviewed and edited the manuscript. W.A.B. conceived the project, acquired funding, provided animal resources, performed transplantation surgery, supervised the project, and reviewed and edited the original draft.

## Supplemental information

Document S1. Figures S1–S6, Tables S1–S4, and supplemental experimental procedures S1–S5

Document S2. Article plus supplemental information
