# Supplementary material for: Systemic immunosuppression promotes survival and integration of subretinally implanted human ESC-derived photoreceptor precursors in dogs
Source: Stem Cell Reports. 2022 Jul 28;17(8):1824–41. doi: 10.1016/j.stemcr.2022.06.009 (PMC9391525; doi:10.1016/j.stemcr.2022.06.009)
Supplement: Document S1. Figures S1–S6, Tables S1–S4, and supplemental experimental procedures S1–S5 [file mmc1.pdf]

Stem Cell Reports, Volume *17*

Supplemental Information

Systemic immunosuppression promotes survival and integration of subretinally implanted human ESC-derived photoreceptor precursors in dogs

Ana Ripolles-Garcia, Natalia Dolgova, M. Joseph Phillips, Svetlana Savina, Allison L. Ludwig, Sara A. Stuedemann, Uchenna Nlebedum, John H. Wolfe, Oliver A. Garden, Arvydas Maminishkis, Juan Amaral, Kapil Bharti, David M. Gamm, Gustavo D. Aguirre, and William A. Beltran

SUPPLEMENTAL FIGURES

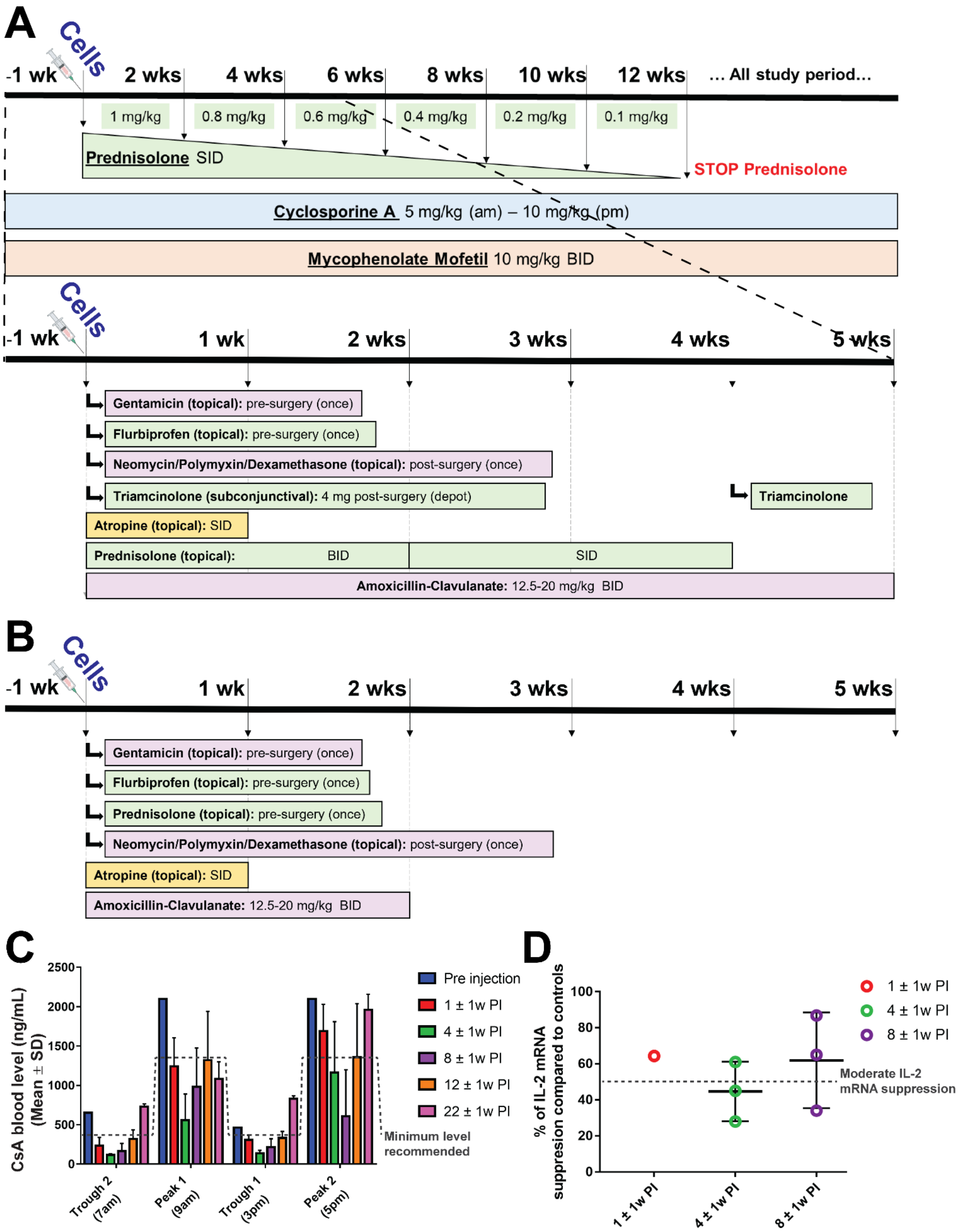
A
Cells
-1 wk
2 wks
4 wks
6 wks
8 wks
10 wks
12 wks
... All study period...
1 mg/kg
0.8 mg/kg
0.6 mg/kg
0.4 mg/kg
0.2 mg/kg
0.1 mg/kg
Prednisolone SID
STOP Prednisolone
Cyclosporine A 5 mg/kg (am) – 10 mg/kg (pm)
Mycophenolate Mofetil 10 mg/kg BID
Cells
-1 wk
1 wk
2 wks
3 wks
4 wks
5 wks
Gentamicin (topical): pre-surgery (once)
Flurbiprofen (topical): pre-surgery (once)
Neomycin/Polymyxin/Dexamethasone (topical): post-surgery (once)
Triamcinolone (subconjunctival): 4 mg post-surgery (depot)
Triamcinolone
Atropine (topical): SID
Prednisolone (topical): BID SID
Amoxicillin-Clavulanate: 12.5-20 mg/kg BID
B
Cells
-1 wk
1 wk
2 wks
3 wks
4 wks
5 wks
Gentamicin (topical): pre-surgery (once)
Flurbiprofen (topical): pre-surgery (once)
Prednisolone (topical): pre-surgery (once)
Neomycin/Polymyxin/Dexamethasone (topical): post-surgery (once)
Atropine (topical): SID
Amoxicillin-Clavulanate: 12.5-20 mg/kg BID
C
CsA blood level (ng/mL) (Mean ± SD)
2500
2000
1500
1000
500
0
Trough 2 (7am)
Peak 1 (9am)
Trough 1 (3pm)
Peak 2 (5pm)
Minimum level recommended
Pre injection
1 ± 1w PI
4 ± 1w PI
8 ± 1w PI
12 ± 1w PI
22 ± 1w PI
D
% of IL-2 mRNA suppresion compared to controls
100
80
60
40
20
0
1 ± 1w PI
4 ± 1w PI
8 ± 1w PI
Moderate IL-2 mRNA suppression
1 ± 1w PI
4 ± 1w PI
8 ± 1w PI

Supplemental Figure S1. Medication regimen given to dogs that received a xenotransplant of hESC-PRPCs. A) Triple-drug immunosuppression (IS) protocol regimen (upper timeline). These animals also received a complementary antibiotic and anti-inflammatory regimen in the initial weeks after surgery (lower timeline). **B**) Antibiotic and anti-inflammatory treatment given in the weeks following surgical cell delivery, in the dogs that did not receive systemic IS. **Pharmacokinetic and pharmacodynamic monitoring of Cyclosporine A (CsA) throughout the study. C**) Pharmacokinetic evaluation of CsA blood levels. The black dashed line marks the minimum recommended by the laboratory to control immune-mediated diseases. **D**) Pharmacodynamic analysis of IL-2 mRNA inhibition, expressed as a percentage of inhibition in comparison with a cohort of normal dogs used by the laboratory. The grey dotted line illustrates 50% of mRNA inhibition, which is considered moderate immune-suppression. Sample collection was not possible at all the time-points due to laboratory intermittent closure. Data are represented as mean ± SD.

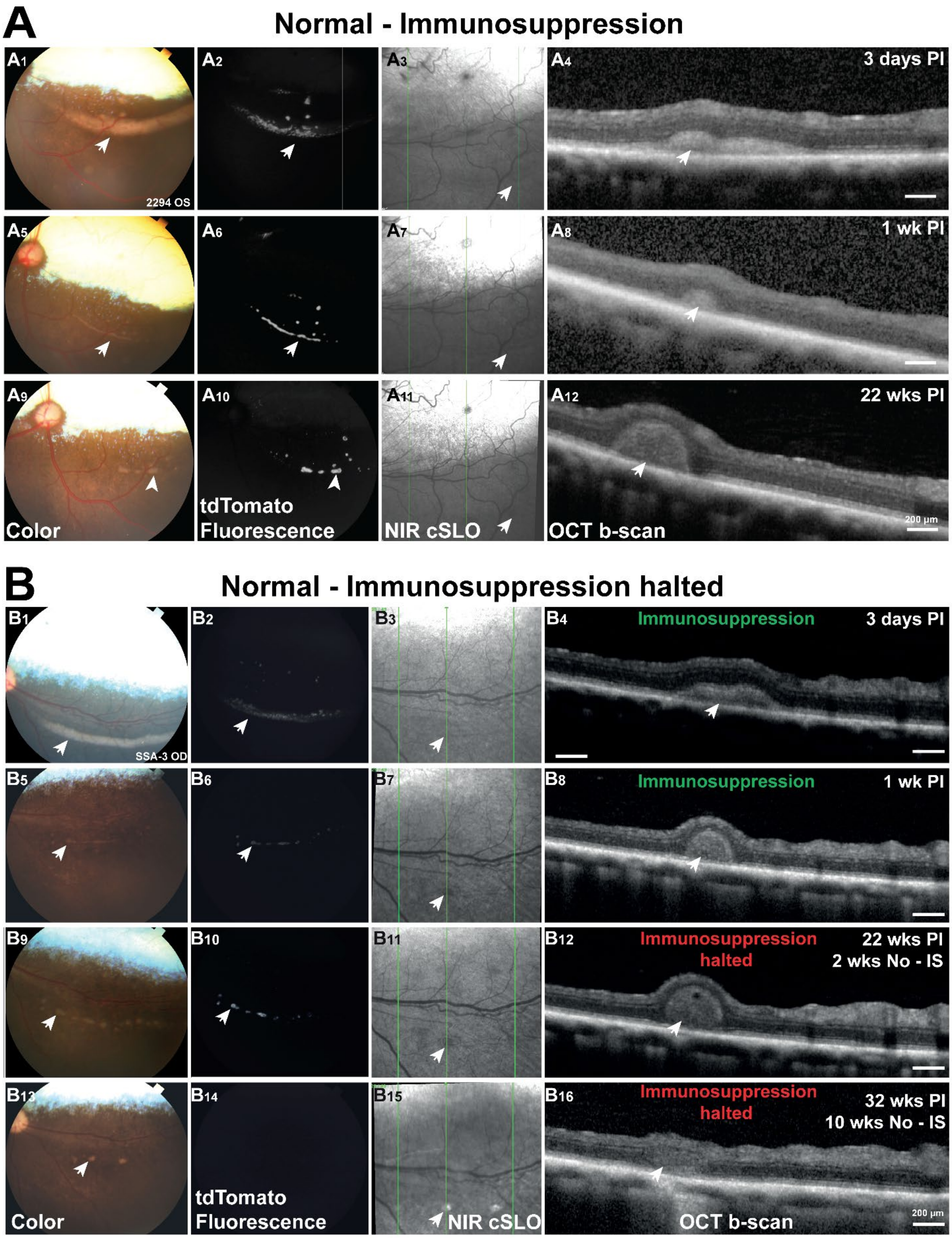

Supplemental Figure S2. Related to Figure 2. Significant donor cell graft loss occurs in the initial week after cell transplantation in the canine subretinal space. Color (A_1, A_5, A_9), fluorescence (A_2,

A_6, A_{10}), NIR cSLO (A_3, A_7, A_{11}), and OCT b-scan (A_4, A_8, A_{12}) retinal imaging acquired 3 days (A_{1-4}), 1 week (A_{5-8}), and 22 weeks (A_{9-12}) post injection (PI). Note that there was an initial cell loss seen between 3 days (A_1, A_4) and 1 week (A_5, A_8) PI, but then there was no major loss up to 22 weeks (A_5, A_9); instead, a reorganization of the graft structure is observed by OCT (A12). As a result of this graft remodeling, the graft area was smaller (A_{10}) but its height is markedly increased (A12). OS: left eye. **Transplant rejection after halting systemic immunosuppression.** Color (B_1, B_5, B_9 and B_{13}), fluorescence (B_2, B_6, B_{10} and B_{14}), NIR cSLO (B_3, B_7, B_{11} and B_{15}), and OCT b-scan (B_4, B_8, B_{12} and B_{16}) retinal imaging acquired three days (B_{1-4}), 1 week (B_{5-8}), 22 weeks (B_{9-12}) and 32 weeks (B_{13-16}) after subretinal cell delivery. In this animal, systemic immunosuppression was halted at 20 weeks after cell transplantation. Two weeks following medication withdrawal, there was an increase in cell mass (B_{12}). Both fluorescence and cells visible by OCT were lost by 10 weeks after medication was halted (B_{14} and B_{16}). OD: right eye.

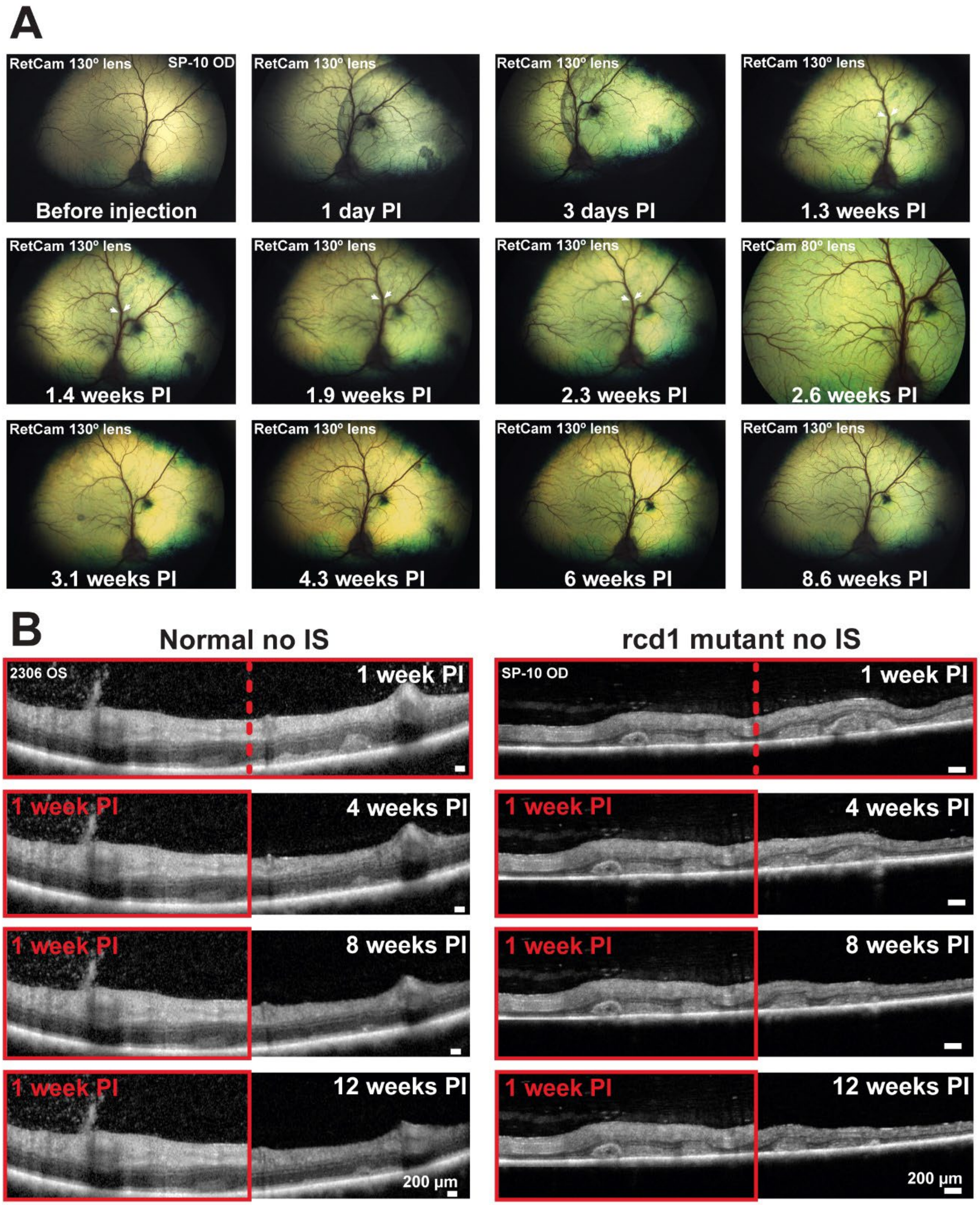

Supplemental Figure S3. Ophthalmoscopic and OCT signs of transplant rejection. Related to figure 2. A) Funduscopic signs of retinal vasculitis (vessel tortuosity, vascular enlargement, and perivascular cuffing) in a normal dog that was not under immunosuppression (IS) regimen. White arrows point at a representative vessel with perivascular cuffing. **B**) OCT alterations (mild retinal swelling,

punctate hyperreflective vitreal foci) in a normal and a mutant dog that were not under IS. Signs were visible at 1 week after transplantation and progressively decreased over the 12 weeks follow-up period. Left half of each panel shows a portion (red box) of the OCT b-scan at 1-week post-injection. OD: right eye; OS: left eye.

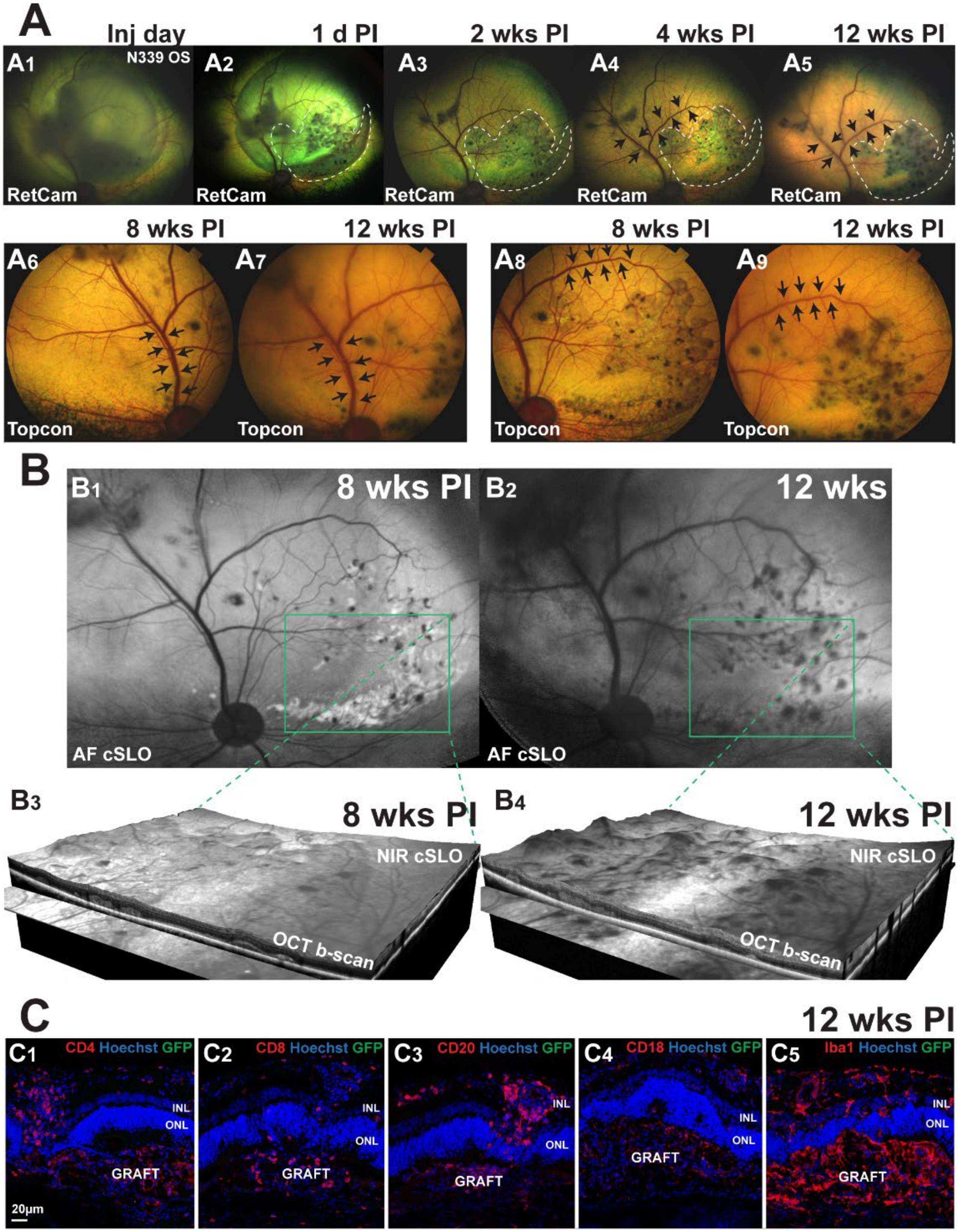
A
Inj day
1 d PI
2 wks PI
4 wks PI
12 wks PI
A1
N339 OS
A2
A3
A4
A5
RetCam
RetCam
RetCam
RetCam
RetCam
8 wks PI
12 wks PI
8 wks PI
12 wks PI
A6
A7
A8
A9
Topcon
Topcon
Topcon
Topcon
B
B1
8 wks PI
B2
12 wks
AF cSLO
AF cSLO
B3
8 wks PI
B4
12 wks PI
NIR cSLO
NIR cSLO
OCT b-scan
OCT b-scan
C
12 wks PI
C1 CD4 Hoechst GFP
C2 CD8 Hoechst GFP
C3 CD20 Hoechst GFP
C4 CD18 Hoechst GFP
C5 Iba1 Hoechst GFP
INL
ONL
GRAFT
20µm

Supplemental Figure S4. Transplant rejection in one normal dog that received systemic immunosuppression regimen. A) RetCam (A_1-A_5) and Topcon color (A_6-A_9) fundus photographs illustrating the subretinal cell mass (white dashed line). The retinal vessels were enlarged and their borders poorly delineated indicating vasculitis at 12 weeks post transplantation (A_5, A_7 and A_9, black arrows) in comparison with the previous time-point evaluated, 8 weeks post injection (A_4, A_6 and A_8, black arrows). The vitreous haziness prevented sharp visualization of the fundus, indicative of vitritis (A_5, A_7 and A_9). **B**) Autofluorescence cSLO imaging confirmed the presence of GFP^+ cells at 8 weeks post-delivery (B_1) but these were no longer visible by 12 weeks post injection (B_2). Generalized retinal swelling was also detected at 12 weeks (B_3) in comparison to 8 weeks post injection (B_4). **C**) A mixed inflammatory cell infiltrate was seen infiltrating the graft and was dominated by microglial cells (C_5). Helper and cytotoxic T-cells (C_{1-2}), B-cells (C_3), and macrophages (C_4) were also present. No donor cells were seen in the subretinal space, and a severe disruption of retinal structure was present. OS: left eye.

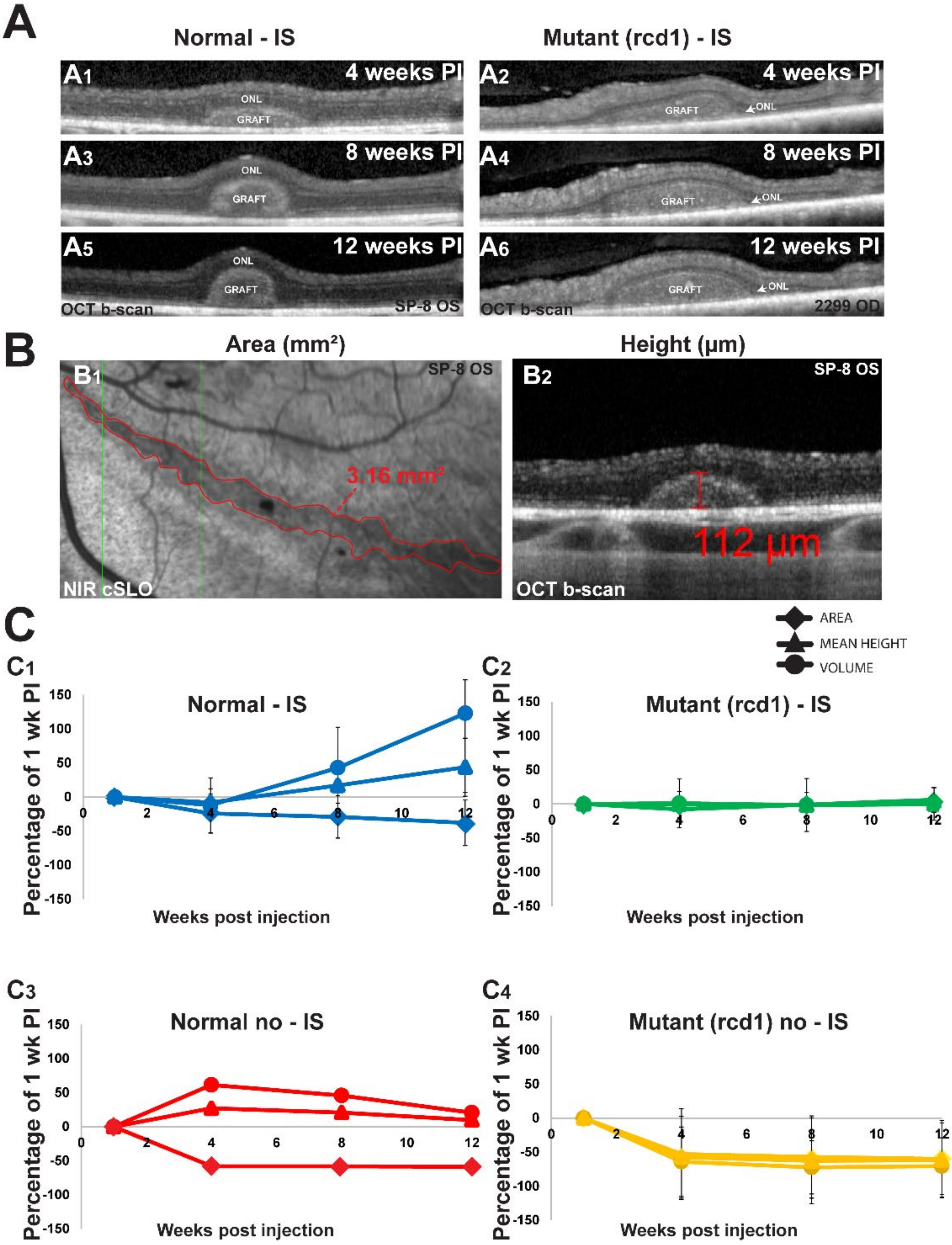
A
Normal - IS
Mutant (rcd1) - IS
A1 4 weeks PI
ONL
GRAFT
A2 4 weeks PI
GRAFT
ONL
A3 8 weeks PI
A4 8 weeks PI
A5 12 weeks PI
A6 12 weeks PI
OCT b-scan
SP-8 OS
2299 OD
B
Area (mm²)
Height (µm)
B1
B2
SP-8 OS
3.16 mm²
112 µm
NIR cSLO
OCT b-scan
C
AREA
MEAN HEIGHT
VOLUME
C1 Normal - IS
C2 Mutant (rcd1) - IS
C3 Normal no - IS
C4 Mutant (rcd1) no - IS
Percentage of 1 wk PI
Weeks post injection

Supplemental Figure S5. Longitudinal characterization of the subretinal graft in normal and mutant animals with and without systemic immunosuppression. A) Representative OCT b scans from normal and mutant dogs under systemic immunosuppression (IS) illustrate morphological changes of the subretinal mass at 4 weeks (A_{1-2}), 8 weeks (A_{3-4}) and 12 weeks post injection (A_{5-6}). **B**) Area (B_1) and height (B_2) of the transplant measured respectively on the NIR cSLO image and OCT b scans. **C**) Changes in cell mass area, height, and volume as a function of time post injection (results are compared to values at 1 week PI). Data are represented as mean ± SD for each group: (C_1) Normal IS (n = 9 eyes, 5 dogs), (C_2) mutant IS (n = 4 eyes, 2 dogs at 4 weeks; n = 3 eyes, 2 dogs at 8 weeks; and n = 2 eyes, 1 dog at 12 weeks post injection), (C_3) normal no-IS (n = 1 eye) and (C_4) mutant no-IS (n = 2 eyes, 1 dog). OD: right eye; OS: left eye.

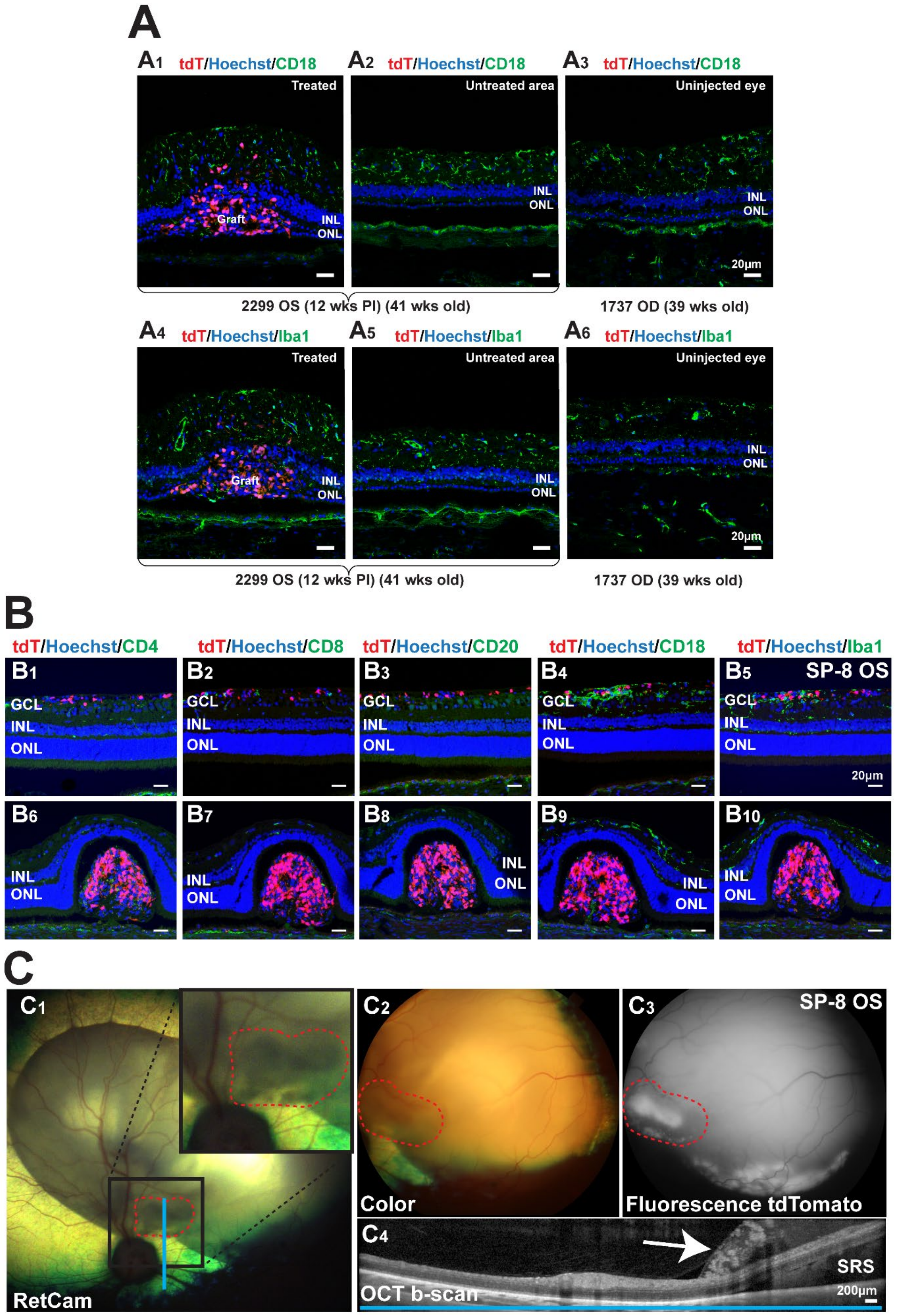
A
A1 tdT/Hoechst/CD18
Treated
Graft
INL
ONL
A2 tdT/Hoechst/CD18
Untreated area
INL
ONL
A3 tdT/Hoechst/CD18
Uninjected eye
INL
ONL
20µm
2299 OS (12 wks PI) (41 wks old)
1737 OD (39 wks old)
A4 tdT/Hoechst/Iba1
Treated
Graft
INL
ONL
A5 tdT/Hoechst/Iba1
Untreated area
INL
ONL
A6 tdT/Hoechst/Iba1
Uninjected eye
INL
ONL
20µm
2299 OS (12 wks PI) (41 wks old)
1737 OD (39 wks old)
B
tdT/Hoechst/CD4
tdT/Hoechst/CD8
tdT/Hoechst/CD20
tdT/Hoechst/CD18
tdT/Hoechst/Iba1
B1 GCL INL ONL
B2 GCL INL ONL
B3 GCL INL ONL
B4 GCL INL ONL
B5 SP-8 OS GCL INL ONL 20µm
B6 INL ONL
B7 INL ONL
B8 INL ONL
B9 INL ONL
B10 INL ONL
C
C1
RetCam
C2
Color
C3 SP-8 OS
Fluorescence tdTomato
C4
OCT b-scan
SRS
200µm

Supplemental Figure S6. Immunohistochemical characterization of CD18+ and Iba1+ cell mediated activation in the inner retina of rcd1/*PDE6B* mutant dogs. Related to figure 3. A) CD18+ cells infiltrating the inner retina of treated (A1) and untreated (A2) areas of the same eye, as well as in age-matched uninjected rcd1/*PDE6B* mutant eye (A3). Iba+ cells in the inner retina of treated (A4) and untreated (A5) areas of the same eye, as well as in an age-matched uninjected rcd1/*PDE6B* mutant eye (A6). Donor cells expressing tdTomato are labeled with red in all panels. OD: right eye; OS: left eye. **Donor cells unintentionally delivered under the inner limiting membrane trigger a more robust inflammatory response than those injected in the subretinal space in the same eye. B**) Located in the inner retina, numerous inflammatory cells consisting primarily of macrophages (CD18) and microglial cells (Iba1) were seen surrounding the donor cells (B1-5). In the same retinal section, inflammatory markers did not show evidence of infiltration of cells directed towards the subretinal transplant (B6-10). **C**) Cell mass (C1-3, red dotted line) found to have been injected under the inner limiting membrane (C4). The OCT b-scan in (C4) is extracted from the area marked with a blue line in (C1). Donor cells expressing tdTomato are labeled with red in all panels. OS: left eye; GCL: ganglion cell layer; INL: inner nuclear layer; ONL: outer nuclear layer.

SUPPLEMENTAL TABLES

Supplemental Table S1. Summary of the longitudinal monitoring by non-invasive imaging of donor cell survival following transplantation.

	Dog ID	Eye	4 ± 1 weeks PI		8 ± 1 weeks PI		12 ± 1 weeks PI		22 ± 1 weeks PI		32 ± 1 weeks PI	
			Fluo	OCT	Fluo	OCT	Fluo	OCT	Fluo	OCT	Fluo	OCT
**Normal IS**	**SP8**	OS	=	=	=	=	=	=				
	**AS2-427**	OD	=	=	=	↓	=	=				
		OS	=	↓	=	↓	=	=				
	**N339**	OD	=	↓	=	=	↓↓↓	↑↑↑				
		OS	=	↓	=	=	↓↓↓	↑↑↑				
	**2294**	OD	=	=	=	=	=	=	=	=		
		OS	=	=	=	=	=	=	=	=		
	**SSA-3**	OD	=	=	=	=	=	=				
		OS	=	=	=	=	=	=				
**Normal No-IS**	**SSA-3** *IS stopped at 20 wks PI*	OD							↓	↑↑↑	↓↓↓	↓↓↓
		OS							↓	↑↑↑	↓↓↓	↓↓↓
	**SP-10**	OD	↓↓↓	↓↓↓	↓↓↓	↓↓↓	↓↓↓	↓↓↓				
**Mutant (rcd1/*PDE6B*) IS**	**2299**	OD	=	=	=	=	=	=				
		OS	=	=	=	=	=	=				
	**2307**	OD	=	↓	=	=						
		OS	=	↓								
**Mutant (rcd1/*PDE6B*) No-IS**	**2306**	OD	↓↓↓	↓↓↓	↓↓↓	↓↓↓	↓↓↓	↓↓↓				
		OS	↓↓↓	↓↓↓	↓↓↓	↓↓↓	↓↓↓	↓↓↓				

IS: immunosuppression; OD: right eye; OS: left eye; Fluo: fluorescence observed by fundus photography and compared to the previous time-point; OCT: volume of cells observed by Optical Coherence Tomography compared to the previous time-point; =: stable; ↓: minimal decrease; ↓↓↓: severe decrease; ↑↑↑: severe increase.

Supplemental Table S2. Summary of immunohistochemistry findings in all processed eyes.

Finding on IHC				Cellular inflammation	Grafted cells displaced into host retina	Potential for synapses	Predominant cone differentiation	Graft proliferation		Cytoplasmic exchange
	**Dog ID**	**Eye**	**Weeks PI**	**CD4, CD8, CD20, CD18, Iba1**	**Ku80**	**PKCα, Goα, Synaptophysin**	**hArrestin3**	**PCNA**	**PHH3**	**$Ku80^-$ / tdTomato or GFP^+**
**Normal IS**	**SP8**	OS	11.6	-	+	+	+	+	-	-
	**SSA-1**	OD	1.6	-	-	-	+	+	NP	-
	**SSA-1**	OS	1.6	-	-	-	+	+	-	-
	**AS2-427**	OD	11.7	-	-	-	+	-	-	-
	**N339**	OD	12.1	+++	-	-	NP	-	-	-
	**N339**	OS	12.1	+++	-	-	NP	-	-	-
	**2294**	OD	20.6	-	+	+	+	-	-	-
	**2294**	OS	20.6	-	+	+	+	-	-	-
**Normal NO-IS**	**SSA-3**	OD	31.6	+++	-	-	NP	+	-	-
	**SP-10**	OD	11.6	+++	-	-	NP	NP	NP	-
**Mutant IS**	**2299**	OD	12.1	-	+++	+	+	-	NP	-
	**2299**	OS	12.1	-	+++	+	+	-	-	-
	**2307**	OD	9	-	+++	+	+	NP	NP	-
	**2307**	OS	9	-	+++	+	+	NP	NP	-
**Mutant NO-IS**	**2306**	OD	12.1	+++	-	-	NP	NP	NP	-
	**2306**	OS	12.1	+++	-	-	NP	-	-	-

IS: immunosuppression; NO-IS: no immunosuppression; OD: right eye; OS: left eye; PI: post injection; NP: not performed; +: minor; +++: severe; -: not detected.

Supplemental Table S3. Quantification of integration events in a cohort of animals.

Group	Eye (dog ID)	Weeks post injection	Frequency of integration	Number of cells injected (Aggr CRX$^{/tdTomato+}$)
Normal IS	Left eye (SP8)	11.6	0/10	4 million in 150 μL
Normal IS	Right eye (AS2-427)	11.7	0/10	2.7 million in 100 μL
Normal IS	Left eye (2294)	20.6	0/10	4 million in 150 μL
Mutant IS	Right eye (2299)	12.1	10/10	4 million in 100 μL
Mutant IS	Left eye (2299)	12.1	4/10	4 million in 100 μL
Mutant IS	Right eye (2307)	9	7/10	2 million in 100 μL

IS: immunosuppression; NO-IS: no immunosuppression.

Supplemental Table S4. List of primary antibodies used for immunohistochemistry.

Antigen	Host	Source, Catalog #	Working concentration	Normal Location
Ku80	Mouse monoclonal IgG1	(STEM101) Takara Bio, Y40400	1:500	Human nuclei
CD4	Rat	Bio-Rad Antibodies, MCA1038GA	1:50	Helper T cells
CD8	Rat monoclonal IgG1	Bio-Rad Antibodies, MCA1039GA	1:50	Cytotoxic T cells
CD18	Mouse monoclonal IgG1	Leukocyte Antigen Laboratory, UC Davis, Sacramento, CA	1:50	Macrophages (blood/monocyte-derived)
CD20	Rabbit	Thermo Fisher scientific, PA5-16701	1:400	B cells
Iba1	Rabbit	FUJIFILM Wako Pure Chemical Corporation, 019-19741	1:500	Microglia
Rod opsin (Rho)	Mouse monoclonal IgG1	Millipore Sigma, MAB5316	1:200	Outer segment of rods
Arrestin 3 (human cone arrestin, hCA)	Goat	Novus Biologicals, NBP1-37003	1:300	Cones (human)
M/L opsin	Rabbit	Millipore Sigma, AB5405	1:200	Red/Green cones
S opsin	Rabbit	Millipore Sigma, AB5407	1:200	Blue cones
PCNA	Mouse monoclonal IgG2a	Santa Cruz Biotechnology, sc-56	1:50	Proliferating cells or cells undergoing DNA repair
Phospho-Histone H3	Rabbit	Cell Signalling Technology, 9701	1:50	Proliferating cells
ZO-1	Rabbit	Thermo Fisher scientific, 40-2200	1:100	Outer limiting membrane
Protein kinase C (PKCα)	Mouse monoclonal IgG2b	BD Bioscience, 610108	1:50	Rod bipolar cells
G Protein Goα	Mouse monoclonal IgG1	Millipore Sigma, MAB3073	1:500	ON (rod and cone) bipolar cells
hSynaptophysin	Mouse	Invitrogen, 14-6525-82	1:100	Photoreceptor presynaptic vesicles (human)
GFAP	Rabbit	DAKO, Z0334	1:300	Müller glia
hGFAP	Mouse monoclonal IgG1	(STEM123) Takara Bio, Y40420	1:500	Human Müller glia

SUPPLEMENTAL EXPERIMENTAL PROCEDURES

Supplemental Experimental Procedures S1.

Details of hESC-PRPCs preparation and subretinal delivery.

Variation between organoids is common, as described by the Gamm lab in their 2019 Development paper (Capowski et al). To mitigate organoid to organoid variability, ~100-200 organoids were pooled for each transplantation, and the same cell line was used for each differentiation.

Stage 2 retinal organoids (ROs) (day 104-151 of differentiation, see Capowksi et al 2019 for description of organoid stages) were shipped overnight from the University of Wisconsin to the University of Pennsylvania in transport medium (Hibernate™ CTS media with 2% FBS, 2% B27, 1% PSA) at 4ºC where they were immediately transferred to retinal differentiation medium (RDM+RA+2% FBS) and maintained at 37ºC in a 5% CO2 controlled incubator (Model SCO6AD, Shel Lab, Cornelius, OR) with media changes performed until processing for transplantation. Following several rounds of washes with Hank's Balanced Salt Solution (HBSS) without calcium and magnesium (HBSS -/-), ROs were diluted in BSS and processed by mechanical fragmentation or enzymatic papain digestion, producing PRPC-rich aggregated or dissociated cell suspensions, respectively (Table 1). Stage 2 ROs were dissociated to single cells with papain (Worthington Biochemical) at 37°C with periodic trituration until organoids were fully dissociated. Following enzymatic deactivation with ovomucoid, cells were passed through a 40 µm filter and resuspended in sterile HBSS -/- to reach the desired cell concentration. Cell viability of 90-99% was routinely obtained following papain dissociation, and cell suspension was maintained on ice until transplantation.

The surgical procedures in dogs were conducted under general anesthesia achieved by propofol induction (4 mg/kg, PropoFlo™ Zoetis, Kalamazoo, MI) and isoflurane maintenance (2–3%; Akorn, Inc. Lake Forest, IL).

<u>Subretinal bolus injection technqiue</u>: The cell suspension containing PRPCs was delivered in the SRS of 10 dogs (18 eyes), using a subretinal injector (RetinaJect, SurModics, CA) (Komaromy et al., 2006) that was custom-modified to replace the 39 gauge polyimide cannula with either a 31 or 33 gauge cannula. In brief, the needle of the subretinal injector was inserted into the vitreal cavity via a pars plana approach and visualized with a Machemer magnifying lens (OMVI; Ocular Instruments Inc., Bellevue, WA, USA) and a surgical microscope (Zeiss Universal S2B Operating Microscope, Oberkochen, Germany). Once the needle was in the posterior vitreous, the inner polyimide cannula (33 or 31 gauge) was extended to contact the retina at the desired location, at which point manual bolus injection of the cell suspension created a retinotomy and a subretinal bleb began to form. The surgical outcome was documented immediately post-injection with a fundus camera (RetCam Shuttle, Clarity Medical Systems, Inc. Pleasanton, CA, USA). Except for one of the normal dogs that had a bilateral vitrectomy performed two years prior to the enrollment in this study, all other animals underwent this subretinal injection without prior vitrectomy.

<u>5-step subretinal injection technique</u>: The entre surgical procedure was performed under direct visualization of the fundus with a digital operating microscope (Zeiss OPMI Lumera 700, Carl Zeiss Meditec AG, Jena, Germany) equipped with intraoperative OCT (Zeiss Rescan 700) and a 3D visualization system (Ngenuity®, Alcon, Fort Worth , TX). Following a complete 3 port pars plana vitrectomy (step 1) (Stellaris, Bausch & Lomb, 25 G instruments), after removal of the vehicle triamcinolone acetonide (Kenalog, 40 mg/mL, Bristol-Myers-Squibb) was resuspended in BSS and a 0.1-0.2 mL (4-8 mg) solution was injected into the vitreal cavity to help with visualization and removal of the posterior hyaloid membrane (step 2). A pneumatic-assisted subretinal injection of BSS (~ 100 µL) was performed using a 38 gauge cannula (PolyTip® cannula 25g/38g, MedOne, Sarasota, FL) with a syringe (MicroDose™ Injector, MedOne) connected to the VFI tubing set of the Stellaris system with a maximal pressure set at 16 PSI (step 3). Following formation of the subretinal bleb, the cannula was maintained in place and slow reaspiration (max aspiration pressure set at 600 mmHg) of ~ 50 µL of BSS was performed (step 4). Delivery of the cells into the same bleb trhough the intial or a second retinotomy site

was perfomed by using a 31 gauge cannula (PolyTip® cannula 25g/31g, MedOne) connected to another MicroDose™ Injector under pressure-controlled assistance (step 5). Intraoperative OCT was used to confirm the formation of the bleb and delivery of cells into the SRS. Video recordings of the surgery were used to document absence of any significant vitreal reflux after retraction of the subretinal cannula.

Supplemental Experimental Procedures S2.

Perioperative pharmacological treatment

On the morning of surgery, all the dogs received a single application of topical antibiotic (Gentamicine sulfate solution 0.3%, Allergan USA, Inc. Madison, NJ) and topical non-steroidal anti-inflammatory (Flubiprofen 0.03%, Bausch+Lomb, Tampa, FL). The animals from the no-IS (Fig. S1 B) group also received a single application of topical corticosteroids (Prednisolone acetate 1% suspension, Allergan, Irvine, CA) preoperatively. A triple topical mydriatic protocol was used in all the dogs before the surgical intervention, that included: tropicamide 1% (Akorn, Inc. Lake Forest, IL), atropine sulfate 1% solution (Akorn, Inc. Lake Forest, IL) and phenylephrine hydrochloride 10% (Paragon Bioteck, Portland, OR), with all three drugs given three times 30 minutes apart. Immediately after the surgery, topical application of antibiotic and steroid ointment (Neomycin Sulfate, Polymyxin B Sulfate and Dexamethasone, Bausch and Lomb, Bridgewater, NJ) was given together with atropine sulfate 1% ointment (Bausch+Lomb, Bridgewater, NJ). This atropine ointment was continued twice a day for the first week PI to all the dogs. Immediately after the surgical procedure, a subconjunctival injection of 4 mg of triamcinolone acetonide 40 mg/mL (Bristol-Myers Squibb, Montreal, Canada) was given to dogs in the IS group (Fig. S1 A); this was repeated at 4 weeks PI. A broad-spectrum oral antibiotic therapy was administered (Amoxicillin trihydrate/clavulanate potassium 12.5-20 mg/kg, twice a day, Dechra Veterinary Prodcuts, Overland Park, KS) during the first two weeks (no IS group) or five weeks (IS group). Prednisolone acetate 1% was also given postoperatively in the IS animals (Fig. S1 A), twice a day for 2 weeks and once a day for another 2 weeks.

Immunosuppression monitoring

Throughout the evaluation period, physical examinations, blood and urine collection were performed to assess if the IS protocol was well tolerated by the dogs and to ensure that proper IS levels were achieved. Complete blood count, biochemical analysis of hematologic and urine samples, and urine culture were carried out before injection and at 1-, 4-, 8-, 12-, and 20-weeks PI (data not shown). PK and PD assays were used to ensure that the CsA blood levels and the IS achieved were adequate (Fig. S1 C-D). CsA therapeutic range was considered optimal when the levels were between 800-1400 ng/ml, 2h post drug administration, or 400-600 ng/ml, before the next dose (Fig. S1 C). For PD evaluation, a molecular PCR-based assay of activated T-cell mRNA IL-2 expression was performed. We considered sufficient IS when the suppression of T-cells, measured indirectly by IL-2 mRNA inhibition, was within 50 to 100% to that of control dogs (moderate to marked IS), as recommended by the laboratory (Pharmacodynamic Laboratory, Mississippi State University, MS) (Fig. S1 D). These assays were carried out at weeks 1, 4, 8, 12, and 20 PI. Since no such analyses are developed for measuring MMF- or prednisolone-related IS in dogs, we evaluated the results of CsA PK/PD assays together with the clinical signs of transplant rejection in order to assess if modifications to the IS regimen were needed.

Supplemental Experimental Procedures S3.

In vivo quantification of the subretinal cell clusters over time

The initial time-point of measurement was one week PI, and subsequent measurements of the same region (using the follow-up tool of the Spectralis unit) at 4-, 8- and 12-weeks PI were compared to the initial value. Two types of measurements were combined to provide information of the transplant modifications: transplant area (mm2) and mean transplant height (µm). The transplant area was measured on the near infrared cSLO image, by delineating the region that co-located with the subretinal cell cluster on OCT to define the donor-host boundary. HEYEX software was used to outline the transplant and estimate the area (Fig. S5 A1). The mean transplant height was measured in all the animals by manually placing the caliper bar (HEYEX software) from the base of the transplant, perpendicular to the RPE, to the innermost detectable limit of the subretinal cells (Fig. S5 A2); this was repeated every 10 scans from the raster scan (every 1.2 mm). The percentage of the cell mass or height compared to that obtained at 1 week PI, was averaged for the animals within each group (normal IS, normal no IS, mutant IS and mutant no IS).

A final combined analysis of the graft volume was estimated. To this end, we assumed that the area and mean height of the transplant formed a geometrical cone shape. Then, we calculated the volume of the "grafted cone" by multiplying the cone area by 1/3 of the cone height. This volume was normalized to that found at 1 week PI and the results were sorted by group.

Supplemental Experimental Procedures S4.

Preparation of immunostained sections.

The eyes were fixed in 4% paraformaldehyde for 3 hours, followed by 2% paraformaldehyde for 24 hours, trimmed, cryoprotected in 15%–30% sucrose/PBS solution, and embedded in optimal cutting temperature media. Ten microns-thick cryosections were obtained (Microm HM550; Thermo Fisher Scientific, Waltham, MA) from the areas where the photoreceptor precursors were previously identified by OCT; the region was localized by landmarks of the retinal vascular pattern in comparison with infrared cSLO images and OCT. As a complementary analysis, archival retinal sections from three uninjected rcd1/*PDE6B* mutant animals of 22, 26 and 39 weeks with both genders represented, were studied by IHC. These were used to accurately interpret the histological findings in the hESC-PRPCs treated mutant dogs.

The sections were stained with different IHC markers (Table S2). Antigen retrieval was performed prior to immunolabelling against ZO-1, PCNA and PHH3 antibodies by two cycles of 125 ºC/1.5 min and 90 ºC/10s in the presence of Antigen Unmasking Solution (Vector Laboratories, Burlingame, CA) using a decloaking chamber (Biocare medical, Concord, CA). The antigen-antibody complexes were visualized with fluorochrome-labeled secondary antibodies (Alexa Fluor, 1:200; Molecular Probes), and Hoechst 33342 nuclear stain (Molecular Probes) was used to label cell nuclei.

Supplemental Experimental Procedures S5.

Immunohistochemical quantification of the integration events in a cohort of normal and mutant animals

The frequency of integration of the major cell cluster was investigated in a cohort of normal and mutant animals. We selected three eyes from the mutant-IS group and three eyes from normal-IS animals for further quantification of the frequency of integration events of the main donor cell cluster into the host retina. In each eye, we identified the main cell cluster located in the pseudohypopyon area based on fundus photography and selected 10 consecutive retinal cryosections, each section being 10 μm thick. In each slide a yes/no answer was recorded according to the presence or absence of migration of the major cell cluster into the host retina.

SUPPLEMENTAL REFERENCES

Capowski, E.E., Samimi, K., Mayerl, S.J., Phillips, M.J., Pinilla, I., Howden, S.E., Saha, J., Jansen, A.D., Edwards, K.L., Jager, L.D., et al. (2019). Reproducibility and staging of 3D human retinal organoids across multiple pluripotent stem cell lines. Development (Cambridge, England) 146. 10.1242/dev.171686.

Komaromy, A.M., Varner, S.E., de Juan, E., Acland, G.M., and Aguirre, G.D. (2006). Application of a new subretinal injection device in the dog. Cell Transplant 15, 511-519. 10.3727/000000006783981701.

Supplemental Video S1. 3D confocal imaging of donor human PRPCs integrated into a normal canine retina and adopting a cone-like morphology. Two tdTomato-positive PRPCs expressing the human nuclear antigen (Ku80) have migrated their cell bodies into the host's ONL. These cells show an elongated photoreceptor-like morphology that includes an inner segment, and an axon extending towards the host's outer plexiform layer and ending with a pedicle-like structure. Images are shown with and without differential interference contrast (DIC)/Nomarski optics.

Supplemental Video S2. 5 step surgical procedure used to optimize in the canine eye the subretinal delivery of cell suspensions by avoiding reflux into the vitreal cavity.
