# Supplementary material for: Systemic immunosuppression promotes survival and integration of subretinally implanted human ESC-derived photoreceptor precursors in dogs
Source: Stem Cell Reports. 2022 Jul 28;17(8):1824–41. doi: 10.1016/j.stemcr.2022.06.009 (PMC9391525; doi:10.1016/j.stemcr.2022.06.009)
Supplement: Document S2. Article plus supplemental information [file mmc4.pdf]

Stem Cell Reports
Article
OPEN ACCESS

Systemic immunosuppression promotes survival and integration of subretinally implanted human ESC-derived photoreceptor precursors in dogs

Ana Ripolles-Garcia,[1] Natalia Dolgova,[1] M. Joseph Phillips,[2,3] Svetlana Savina,[1] Allison L. Ludwig,[2,3] Sara A. Stuedemann,[2] Uchenna Nlebedum,[2] John H. Wolfe,[5,6] Oliver A. Garden,[1,10] Arvydas Maminishkis,[7] Juan Amaral,[8] Kapil Bharti,[9] David M. Gamm,[2,3,4] Gustavo D. Aguirre,[1] and William A. Beltran[1,*]

[1]Division of Experimental Retinal Therapies, Department of Clinical Sciences and Advanced Medicine, School of Veterinary Medicine, University of Pennsylvania, Philadelphia, PA 19104, USA
[2]Waisman Center, University of Wisconsin-Madison, Madison, WI 53705, USA
[3]McPherson Eye Research Institute, University of Wisconsin-Madison, Madison, WI 53705, USA
[4]Department of Ophthalmology and Visual Sciences, University of Wisconsin-Madison, Madison, WI 53705, USA
[5]Walter Flato Goodman Center for Comparative Medical Genetics, School of Veterinary Medicine, University of Pennsylvania, Philadelphia, PA 19104, USA
[6]Children's Hospital of Philadelphia, Philadelphia, PA 19104, USA
[7]Section on Epithelial and Retinal Physiology and Disease, National Eye Institute, NIH, Bethesda, MD 20892, USA
[8]Office of Scientific Director, National Eye Institute, NIH, Bethesda, MD 20892, USA
[9]Unit on Ocular and Stem Cell Translational Research, National Eye Institute, NIH, Bethesda, MD 20892, USA
[10]Present address: School of Veterinary Medicine, Louisiana State University, Baton Rouge, LA 70803, USA
*Correspondence: wbeltran@vet.upenn.edu

https://doi.org/10.1016/j.stemcr.2022.06.009

SUMMARY

Regenerative therapies aimed at replacing photoreceptors are a promising approach for the treatment of otherwise incurable causes of blindness. However, such therapies still face significant hurdles, including the need to improve subretinal delivery and long-term survival rate of transplanted cells, and promote sufficient integration into the host retina. Here, we successfully delivered *in vitro*-derived human photoreceptor precursor cells (PRPCs; also known as immature photoreceptors) to the subretinal space of seven normal and three rcd1/*PDE6B* mutant dogs with advanced inherited retinal degeneration. Notably, while these xenografts were rejected in dogs that were not immunosuppressed, transplants in most dogs receiving systemic immunosuppression survived up to 3–5 months postinjection. Moreover, differentiation of donor PRPCs into photoreceptors with synaptic pedicle-like structures that established contact with second-order neurons was enhanced in rcd1/*PDE6B* mutant dogs. Together, our findings set the stage for evaluating functional vision restoration following photoreceptor replacement in canine models of inherited retinal degeneration.

INTRODUCTION

Mammalian photoreceptors, the light-sensing outer retinal cells, lack self-regenerative capacity, and their degeneration in inherited retinal disease (IRD) is a major cause of blindness. However, while photoreceptors are lost in human IRDs and the dry form of age-related macular degeneration (AMD), the inner retinal structure is retained, even in advanced disease (Aghaizu et al., 2017). As such, regenerative therapies aimed at replacing photoreceptors and establishing functional synapses with the remaining viable inner retinal neurons is a promising approach for restoring vision recovery in otherwise blind patients.

Human embryonic stem cells (hESCs) or induced pluripotent stem cell (iPSC)-derived photoreceptor precursor cells (PRPCs) that develop into retinal organoids (ROs) are a promising source for regenerative experimental therapies to restore photoreceptor function (Ludwig and Gamm, 2021). Cell replacement studies in rodents have shown restoration of visual function using either ESC- or iPSC-PRPCs (Ribeiro et al., 2021; Tu et al., 2019), and several clinical trials in humans are exploring the use of allogenic and autologous cell therapies for photoreceptor replacement (Wang et al., 2020). However, exogenous photoreceptor cell replacement therapies face critical challenges (Ludwig and Gamm, 2021), including the need to (1) successfully deliver large numbers of cells subretinally to cover a large area, (2) ensure long-term photoreceptor survival rate by preventing immune rejection, and (3) promote sufficient integration into the host retina to recover visual function (Singh et al., 2018).

The dog offers unique advantages for advancing experimental retinal therapies (Ludwig and Gamm, 2021). Multiple models of dogs with naturally occurring mutations in some of the genes responsible for several forms of human IRDs are available and exhibit similar biological, pathological, and functional deficits as those reported for the human disease (Bunel et al., 2019). In addition, the size of the canine eye allows development and optimization of surgical and therapeutic approaches that can then be used in patients, and outcomes can be monitored using instruments commonly used in human clinics.

Here, we compared the ability of hPRPCs to survive and integrate in normal and in rcd1/*PDE6B* mutant dogs, an IRD model that undergoes early-onset progressive retinal degeneration primarily affecting rods. These mutant dogs undergo an acute phase of rods loss at 4–6 weeks of age

This is an open access article under the CC BY license (http://creativecommons.org/licenses/by/4.0/).

Table 1. Details of cell transplantation and monitoring time points in all study animals

	Dog ID (sex, age in weeks at injection)	Eye	Genotype (disease)	Cell suspension type	Estimation of cells delivered in the SRS	Donor cells age at transplant/ euthanasia (days)	1 Week[a]	4 Weeks[a]	8 Weeks[a]	12 Weeks[a]	16 Weeks[a]	22 Weeks[a]	32 Weeks[a]
Normal IS	SP-8 (M, 45)	OS	Wild type (normal)	Aggr CRX$^{/tdTomato+}$	4 million in 150 μL	D107/D188	LI	LI	LI	LI/IHC			
	AS2-427 (F, 161)	OD	Wild type (normal)	Aggr CRX$^{/tdTomato+}$	2.7 million in 100 μL	D151/D233	LI	LI	LI	LI/IHC			
		OS		Aggr CRX$^{/tdTomato+}$	2.7 million in 100 μL	D151/D233	LI	LI	LI	LI/IHC			
	N339 (M, 19)	OD	Wild type (normal)	Aggr NRL$^{+/EGFP}$	4 million in 100 μL	D130/D215	LI	LI	LI	LI/IHC			
		OS		Aggr NRL$^{+/EGFP}$	4 million in 100 μL	D130/D215	LI	LI	LI	LI/IHC			
	2294 (F, 80)	OD	Wild type (normal)	Diss CRX$^{/tdTomato+}$	4 million in 150 μL	D133/D277	LI	LI	LI	LI	LI	LI/IHC	
		OS		Aggr CRX$^{/tdTomato+}$	4 million in 150 μL	D133/D277	LI	LI	LI	LI	LI	LI/IHC	
	SSA-1 (M, 131)	OD	Wild type (normal)	Aggr CRX$^{/tdTomato+}$	2 million in 150 μL	D109/D120	LI/IHC						
		OS		Aggr CRX$^{/tdTomato+}$	4 million in 150 μL	D110/D120	LI/IHC						
	SSA-3 (M, 76)	OD	Wild type (normal)	Aggr CRX$^{/tdTomato+}$	4 million in 150 μL	D133/D354	LI	LI	LI	LI	LI		
		OS		Aggr CRX$^{/tdTomato+}$	4 million in 150 μL	D133/D354	LI	LI	LI	LI	LI		
Normal No-IS	SSA-3 (M, 76)	OD	Wild type (normal)									LI	LI/IHC
		OS										LI	LI/IHC
	SP-10 (M, 45)	OD	Wild type (normal)	Aggr CRX$^{/tdTomato+}$	4 million in 150 μL	D104/D185	LI	LI	LI	LI/IHC			

(*Continued on next page*)

Table 1. *Continued*

	Dog ID (sex, age in weeks at injection)	Eye	Genotype (disease)	Cell suspension type	Estimation of cells delivered in the SRS	Donor cells age at transplant/ euthanasia (days)	1 Week[a]	4 Weeks[a]	8 Weeks[a]	12 Weeks[a]	16 Weeks[a]	22 Weeks[a]	32 Weeks[a]
Mutant IS	2299 (M, 29)	OD	*Pde6β*$^{-/-}$ (rcd1)	Aggr CRX$^{tdTomato+}$	4 million in 100 μL	D125/D210	LI	LI	LI	LI/IHC			
		OS		Aggr CRX$^{tdTomato+}$	4 million in 100 μL	D125/D210	LI	LI	LI	LI/IHC			
	2307 (F, 29)	OD	*Pde6β*$^{-/-}$ (rcd1)	Aggr CRX$^{tdTomato+}$	2 million in 100 μL	D125/D188	LI	LI	LI/IHC				
		OS		Aggr CRX$^{tdTomato+}$	2 million in 100 μL	D125/D188	LI	LI	LI/IHC				
Mutant No IS	2306 (F, 29)	OD	*Pde6β*$^{-/-}$ (rcd1)	Aggr CRX$^{tdTomato+}$	4 million in 100 μL	D125/D210	LI	LI	LI	LI/IHC			
		OS		Aggr CRX$^{tdTomato+}$	2 million in 100 μL	D125/D210	LI	LI	LI	LI/IHC			

Aggr, aggregated; D, day; Diss, dissociated; F, female; ID, identification number; IHC, immunohistochemical assessment; IS, immunosuppression; LI, life imaging; M, male; OD, right eye; OS, left eye; SRS, subretinal space.

[a]Weeks posttransplantation ±1 week.

that is followed by a progressive degeneration of rods and cones over the course of several months (Genini et al., 2013). Using a subretinal injector that was modified to accommodate hPRPC aggregates derived from ROs, we successfully delivered fluorescently tagged hPRPCs into the canine subretinal space (SRS) using a simple surgical procedure. A non-invasive multimodal imaging approach was used to assess cell viability and distribution and also enabled longitudinal analysis of individual cell clusters. A systemic immunosuppressive (IS) protocol of oral prednisolone, cyclosporine A (CsA), and mycophenolate mofetil (MMF) preserved-long term survival of hPRPC-derived cells. Donor cells in normal dogs receiving IS remained mostly in the SRS and attempted to create structural synapses but were largely unsuccessful, likely because of their inability to penetrate the intact outer limiting membrane (OLM). In contrast, the OLM of mutant dogs receiving IS was focally disrupted, and hPRPCs had extended axons and structural formations that resembled synaptic terminals, suggesting that disease-related retinal changes enhance the potential of hPRPCs to form synapses when properly integrated and polarized within the retina.

RESULTS

Multimodal retinal imaging enables non-invasive monitoring of hESC-PRPCs injected into the SRS of normal and degenerated canine retinas

To accommodate the larger size of hPRPC aggregates obtained from ROs, we performed the subretinal delivery of donor cells using a custom-modified subretinal injector with a 31G or 33G cannula. With this approach, a prior vitrectomy was not required, and the cells could be delivered by bolus injection controlled by manual pressure. Human ESC-PRPCs derived from one of two photoreceptor reporter lines (either WA09 *CRX*-tdTomato or WA09 *NRL*-EGFP) (Phillips et al., 2018a, 2018b) were successfully injected into the SRS of normal (12 eyes) and rcd1/*PDE6B* mutant (6 eyes) dogs without surgical complications. Details are listed in Table 1. In all dogs, some of the cell suspension was seen backflowing into the vitreous at the time of the bolus injection before the retinotomy was formed and the bleb began to expand. In non-vitrectomized eyes (n = 16), retinal blebs reattached within 24–48 h, while in the two vitrectomized eyes, 6–7 days were needed for full reattachment.

Donor cells were evaluated *in vivo*, at different time points, using a multimodal imaging approach that included color fundus photography, a custom-modified Topcon retinal camera to image tdTomato fluorescence, autofluorescence (AF) confocal scanning laser ophthalmoscopy (cSLO) to image EGFP, near-infrared (NIR) cSLO,

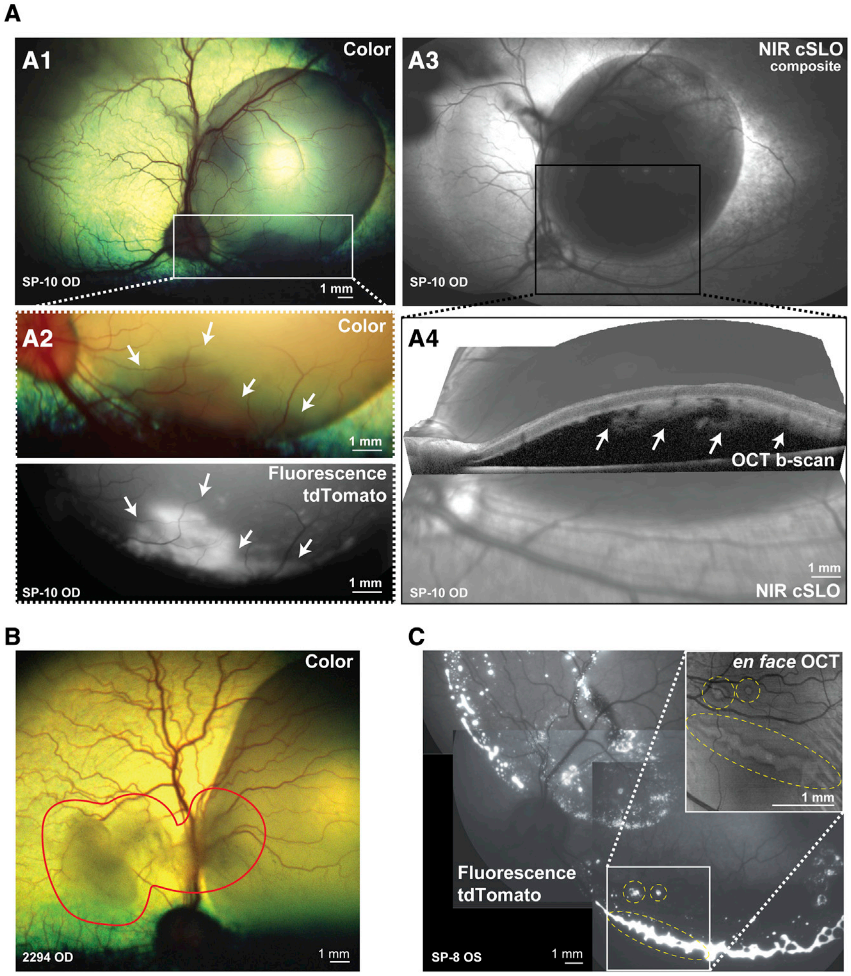

Figure 1. Multimodal retinal imaging following subretinal transplantation of hESC-CRX$^{+/tdTomato}$-derived photoreceptor precursor cells (PRPCs)

(A1) Immediate post-operative color fundus photograph (RetCam camera) showing the subretinal bleb. (A2) Color and fluorescent fundus photograph (modified Topcon retinal camera) showing a cluster of subretinally transplanted cells (white arrows) expressing tdTomato. (A3) NIR cSLO image (Spectralis HRA/OCT2) of the bleb. (A4) Three-dimensional view combining NIR cSLO and OCT b-scan confirming the presence of transplanted cells in the subretinal space.

(B) Immediate post-operative color fundus photograph illustrating the presence of donor cells within the vitreous (area enclosed within red curved line).

(C) Collage of fundus fluorescence photographs of tdTomato-expressing donor cells visualized 1 week post-injection along the border of the subretinally treated area. Inset: *en face* OCT image of the slab delimited by the photoreceptor inner and outer segments and the retinal pigment epithelium confirms the location of the donor cell cluster in the subretinal space.

cSLO, confocal scanning laser ophthalmoscope; NIR, near infrared; OCT, optical coherence tomography; OD, right eye; OS, left eye.

spectral-domain optical coherence tomography (OCT), and *en face* OCT. Importantly, we were also able to longitudinally follow individual cell clusters and evaluate subclinical signs of transplant rejection.

Using this multimodal approach, we evaluated each recipient for the presence of tdTomato- or EGFP-positive cells in the surgically created bleb, confirming a successful injection (Figures 1A1 and 1A2). NIR cSLO and OCT b-scans were next used to verify the subretinal location of transplanted hPRPCs (Figures 1A3 and 1A4). The backflow of cells into the vitreous was confirmed and documented by fundus photography (Figure 1B). In all eyes, subretinally injected cells were mostly distributed in a major cluster, located in the inferior border of the bleb (Figure 1C), likely because of gravitational deposition as dogs were repositioned upright following surgery, acquiring the appearance of a subretinal pseudohypopyon when examined ophthalmoscopically (Figures 1A1 and 1A2). Additional smaller clusters of cells were seen throughout the bleb when using fluorescence retinal imaging and *en face* OCT (Figure 1C). Although the delivered cell dose was estimated prior to injection (Table 1), variation in the size of cell clusters prevented exact quantification of the number of subretinally delivered PRPCs in the SRS and vitreous.

Systemic immunosuppression is necessary for long-term survival of xenotransplants in the canine SRS

A cohort of normal (n = 11 eyes) and rcd1/*PDE6B* mutant (n = 4 eyes) dogs received systemic IS and topical medication to prevent xenograft rejection (Figure S1). The IS regimen combined oral prednisolone, CsA, and MMF. CsA pharmacokinetic (PK) and pharmacodynamic (PD) analyses confirmed that overall, CsA levels were above the minimum recommended by the laboratory (Clinical Pharmacology Laboratory, Auburn University, AL, USA) (Figure S1C), and there was moderate interleukin-2 (IL-2) mRNA suppression (Figure S1D). This IS protocol was well tolerated in almost all the dogs and resulted in effective IS in seven out of eight animals. The one exception

developed palpebral, interdigital, and oral papillomatous formations and viral plaques consistent with canine papillomavirus infection, likely facilitated by reduced immune competence (data not shown).

Although the graft volume decreased between 3 days and 1 week postinjection (PI) in IS animals (Figures S2A1–S2A8), no further substantial cell loss was seen up to 22 weeks PI (Figures S2A9–S2A12). Most animals received aggregated cells (n = 17 eyes), and one animal received enzymatically dissociated cells in one eye. Notably, there was good hPRPC survival after the initial 1-week post-surgery loss in dogs under the triple oral IS protocol in both mutant and normal animals (Figure 2A). In contrast, transplanted cell survival was significantly impaired in both normal and mutant dogs not receiving IS treatment (Table S1), as revealed by a substantial and continuous decrease in fluorescence (Figures 2B2, 2B6, and 2B10) and in the size of the subretinal cell clusters (Figures 2B4, 2B8, and 2B12), that continued until the cells were no longer detectable (Figures 2B9–2B12).

Loss of PRPCs in non-IS animals was not accompanied by severe ocular signs of inflammation, but a mild vasculitis characterized ophthalmoscopically by vessel tortuosity and perivascular cuffing was observed as early as 1 week PI (Figure S3A). By OCT, generalized retinal swelling and a hyperreflective material in the vitreous was seen at 1 week PI, suggestive of transplant rejection (Figure S3B). These OCT features were also observed in one animal in which the IS regimen was intentionally halted (ID: SSA-3) 2 weeks after medication withdrawal. In this animal, there was an increase in the volume of the subretinal cell mass at this time (Table 2; Figures S2B5–S2B12) that was followed by a drastic decrease in both fluorescence and cell mass volume in the SRS by 10 weeks after IS withdrawal (Table 2; Figures S2B13–S2B16), possibly reflecting graft infiltration and clearance by inflammatory cells.

Surprisingly, in the single animal injected bilaterally with WA09 NRL$^{+/EGFP}$ hESCs (ID: N339), in which only rod precursors express EGFP, there were severe funduscopic signs of transplant rejection despite IS treatment (Figure S4), which occurred as the prednisolone regimen was tapered down according to the protocol (12 weeks PI). Notably, in this dog, the cell mass that reached the SRS was markedly larger than in other eyes (Figures S4A2–S4A5). By 12 weeks PI, clinical signs of vasculitis and vitreal haze were visible ophthalmoscopically (Figures S4A5, S4A7, and S4A9) and EGFP fluorescence was no longer detectable (Figure S4B2). OCT revealed retinal swelling with multifocal retinal detachments, pre-retinal vitreal condensation, and an increase in subretinal cell mass volume (Figure S4B4). The retinal inflammation was confirmed by immunohistochemistry (IHC) (Figure S4C).

Longitudinal imaging of transplanted donor cells indicates different patterns of donor cell integration depending on host retinal status

Qualitative assessment of the cluster of donor cells that deposited in the ventral region of the bleb showed changes in shape over time that differed between normal and rcd1/*PDE6B* mutant dogs (Figure S5A). To further analyze these changes, we measured the area occupied by the main cell cluster and its mean height in all eyes using the HEYEX software (Figure S5B) to estimate its volume. In animals that received IS, two distinctive trends were seen based on the host's retinal status. In normal animals, the cell mass area decreased (38% at 12 weeks PI), but its height increased over time (44% 12 weeks after transplantation) (Figures S5A1, S5A3, S5A5, and S5C1). In mutant animals, there were no changes to the area or height of the donor cell mass, likely reflecting less intercellular remodeling within the transplant (Figures S5A2, S5A4, S5A6, and S5C2).

In normal animals not treated with IS, there was an initial increase in height and volume with a decrease in area (59% at 12 weeks PI), possibly reflecting immune rejection and infiltration of inflammatory cells within the first 4 weeks PI (Figure S5C3). In two out of two eyes from the rcd1/*PDE6B* mutant dog that was not under IS, the graft area decreased progressively (60% at 12 weeks PI), indicating poor cell survival and clearance of donor cells from the SRS (Figure S5C4).

Histological evaluation confirms that immunosuppression must be maintained to prevent xenotransplant rejection

When evaluated histologically, the retinas of animals that received IS had no to very limited inflammatory cells infiltrating the transplant (Figures 3A and 3B), except for one normal animal injected with NRL$^{+/EGFP}$ hESCs (Figure S4C). In this animal, perivascular inflammatory cells composed of a mixed inflammatory cell infiltrate confirmed the clinical diagnosis of retinal vasculitis (Figure S4C), and the absence of EGFP-positive donor cells in the SRS and host retina, coupled with severe disruption of retinal lamination, confirmed transplant rejection.

In both non-IS normal and mutant dogs, we found a mixed inflammatory infiltrate composed mostly of macrophages (CD18$^+$; Figures 3C4 and 3D4) and resident microglia (Iba1$^+$; Figures 3C5 and 3D5) and to a lesser extent by helper and cytotoxic T cells (CD4$^+$ and CD8$^+$, respectively; Figures 3C1, 3C2, 3D1, and 3D2) and B cells (CD20$^+$; Figures 3C3 and 3D3), suggesting a robust activation of the innate immune response and to a lesser extent a cellular adaptive immune response. In addition, the number of donor cells (tdTomato- or EGFP- and Ku80-positive cells) was minimal to absent at this time, and there was

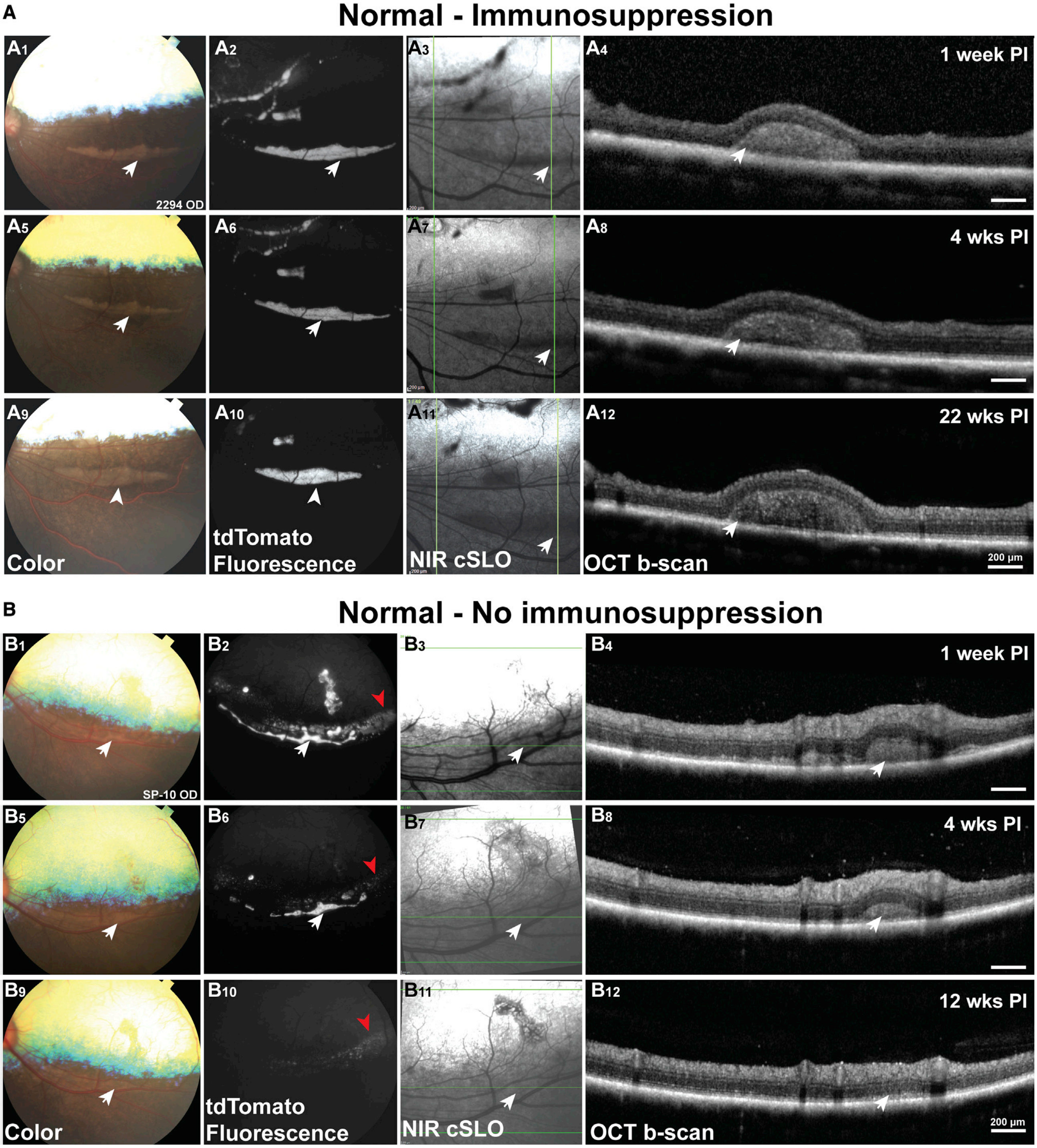

Figure 2. Longitudinal imaging of the subretinal cell mass in dogs with and without immunosuppression (IS)

(A) Color (A1, A5, and A9), fluorescence (A2, A6, and A10), NIR cSLO (A3, A7, and A11), and OCT b-scan (A4, A8, and A12) retinal imaging acquired 1 (A1–A4), 4 (A5–A8), and 22 weeks (A9–A12) after subretinal cell delivery in one animal that was under systemic IS.

(B) Color (B1, B5, and B9), fluorescence (B2, B6, and B10), NIR cSLO (B3, B7, and B11), and OCT b-scan (B4, B8, and B12) retinal imaging acquired 1 (B1–B4), 4 (B5–B8), and 12 weeks (B9–B12) after subretinal cell delivery in one animal that did not receive systemic IS treatment. White arrows point to the cell masses in the subretinal space. Red arrows point to low-intensity background autofluorescence, seen in only one animal not under IS, that was confirmed by histology to originate from hypertrophied host RPE cells filled with lipofuscin-like fluorescent material (data not shown).

Table 2. Percentage of the cell mass volume compared with that at 1 week posttransplantation

Group	Dog ID	Eye	4 ± 1 week PI	8 ± 1 week PI	12 ± 1 week PI	22 ± 1 week PI	30 ± 1 week PI
Normal IS	SP-8	OS	−23	115	121		
	2294	OD	−24	−6	−9	15	
		OS	36	71	118	283	
	AS2-427	OD	70	115	241		
		OS	−9	97	102		
	N339	OD	−53	−36	285		
		OS	−43	−26	−53		
	SSA-3	OD	−43	13	58		
		OS	−19	42	243		
	Mean (SD)		−12 (40)	43 (60)	123 (116)	145 (190)	
Normal no IS	SSA-3	OD				718	−21
		OS				529	410
	SP-10	OD	61	46	20		
	Mean (SD)		61	46	20	623 (133)	195 (305)
Mutant IS	2299	OD	36	41	17		
		OS	19	−11	−17		
	2307	OD	−47	−35			
		OS	−5				
	Mean (SD)		1 (36)	−2 (39)	0.2 (24)		
Mutant no IS	2306	OD	−100	−100	−100		
		OS	−28	−45	−41		
	Mean (SD)		−64 (51)	−72 (39)	−71 (42)		

ID, identification number; IS, systemic immunosuppression; OD, right eye; OS, left eye; PI, postinjection.

an associated disruption of the normal host's retinal architecture (Figures 3C and 3D).

In all cases, rcd1/*PDE6B* mutant dogs under IS (n = 4 eyes) exhibited a diffuse infiltration of microglia and blood-derived macrophages (Iba1$^+$ and CD18$^+$ cells, respectively; Figures 3B4 and 3B5) throughout the inner retina, in both treated and untreated areas (Figures S6A1, S6A2, S6A4, and S6A5). These findings were also observed in archival tissues from uninjected rcd1/*PDE6B* mutant dogs of 22, 26, and 39 weeks of age (Figures S6A3 and S6A6). Thus, we consider this cellular infiltration a normal feature of the neuroinflammation that is associated with the natural course of retinal degeneration rather than a consequence of the delivery of hESC-PRPCs.

Unexpectedly, several small, isolated clusters of PRPCs were detected in the ganglion cell layer (GCL) of two normal dogs that were under IS (Figures S6B1–S6B5). Because we did not see a subretinal cell mass nor structural retinal modification in this region, these cells were likely unintentionally delivered under the inner limiting membrane at the time of injection (Figures S6C1–S6C3, area enclosed within red dashed line). Indeed, OCT imaging performed immediately after transplantation showed a subgroup of cells deposited between the inner limiting membrane and the nerve fiber layer (NFL) in one of these animals (Figure S6C4, white arrow). Within the GCL, these cells triggered a moderate immune response dominated by blood-derived macrophages (CD18$^+$) and microglia (Iba1$^+$) (Figures S6B1–S6B5). In this same eye, PRPCs within the immune-privileged SRS did not trigger a similar inflammatory cell reaction (Figures S6B6–S6B10).

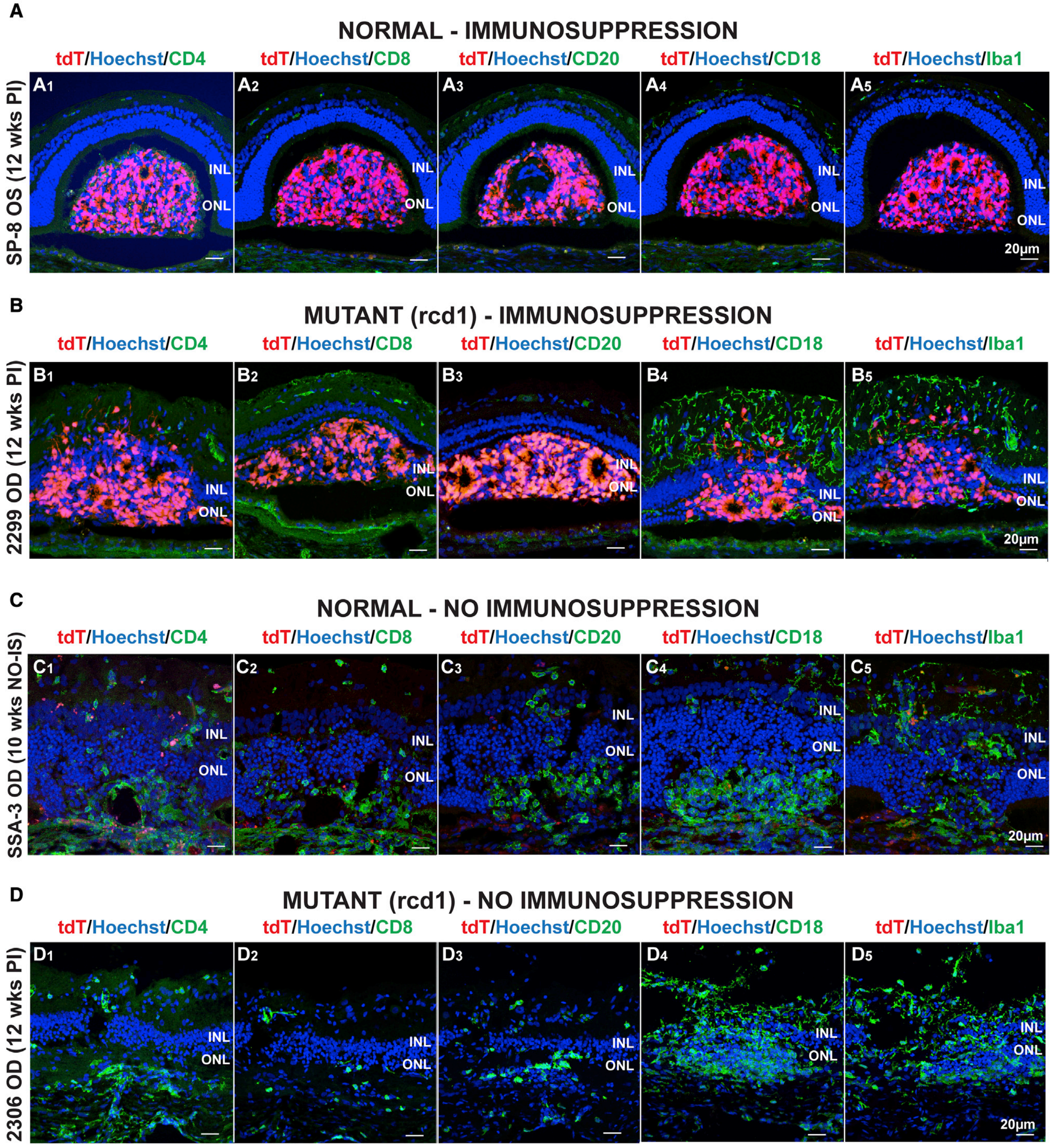

Figure 3. Immunohistochemical characterization of cell-mediated response toward transplanted cells in normal and mutant dogs under or without IS

(A) Absence of immune cells and survival of transplanted donor tdTomato-positive cells in the subretinal space of a normal dog under IS (A1–A5).

(B) Survival and migration of transplanted donor tdTomato-positive cells into the host retina of a mutant (rcd1/*PDE6B*) dog with no evidence of immune T (B1-2) or B (B3) cell infiltration into the graft. Rare macrophage (B4) and microglial (B5) cells were seen in the graft but were mostly found throughout the inner retina.

(legend continued on next page)

Mature donor photoreceptors in the SRS were mostly M/L cones

We evaluated the fate of PRPCs by assessing the expression of markers of mature human and canine rods (rhodopsin [Rho]), mature human cones (human cone arrestin [hCA]), as well as canine and human cones (M/L opsin and S opsin). Rho expression suggested that human rods were present (Figures 4A1 and 4A5). However, we consistently saw that most donor photoreceptors were cones (Figures 4A2 and 4A6), particularly M/L cones (Figures 4A3 and A7) and, to a lesser extent, S cones (Figures 4A4 and 4A8).

To rule out any tumorigenic potential of the graft, we assessed proliferating cell nuclear antigen (PCNA), a marker of retinal proliferation, and DNA repair in dogs and phospho-histone H3 (PHH3), specific to cells undergoing mitosis. None of the donor cell clusters contained any PHH3-positive cells (Figures 4A9 and 4A12). Positive PCNA labeling within the donor cell mass was seen in only four normal dogs (with and without IS) and was limited to fewer than 10 cells per section inside the main cluster (Figures 4A10 and 4A13).

Human PRPCs show anatomic integration into the canine retina and structural potential for synaptic connectivity

We next evaluated the integrity of the host OLM by staining for zonula occludens-1 (ZO-1), a tight junction protein present in the OLM. In normal retinas, the OLM was a thick, continuous structure (Figure 4A11, white arrow). On the contrary, in mutant animals, the OLM was focally disrupted and did not have a regular thickness (Figure 4A14, white arrow). This finding was confirmed in uninjected rcd1/*PDE6B* mutant retinas at 22, 26, and 39 weeks of age (data not shown).

Although donor PRPCs were originally thought to integrate into the recipient retina, recent reports in rodents indicate that donor-host cytoplasmic material exchange can also occur (Ortin-Martinez et al., 2017). To assess potential exchange of donor-host cytoplasmic material in dogs following transplantation of human PRPCs, we undertook IHC analysis in which the cell nuclei of donor human cells (Ku80$^+$) was immunostained in parallel with the other markers of interest. Notably, we did not observe any evidence of cellular material exchange in any of the retinas at the different time points evaluated, because all the cells that expressed tdTomato or GFP also had Ku80-positive nuclei (Table S2).

In normal animals that received IS treatment, donor cells remained mostly in the SRS (Figures 5A1–5A3), with a few PRPCs from the main cluster sending cytoplasmic projections toward the outer nuclear layer (ONL) (Figures 5A1–5A3, white dashed circles). However, the somata of these PRPCs remained unable to penetrate the normal OLM. Such attempts to create structural synapses were seen as early as 12 and 20 weeks PI (but not at 2 weeks PI). In addition, sporadic smaller clusters of donor cells migrated into the ONL, outer plexiform layer (OPL), and inner nuclear layer (INL) in three out of eight normal retinas under IS (Figures 5A4–5A8; Video S1). Some of these cells exhibited photoreceptor morphology, with short outer segment (OS)-like structures and axon extension polarized toward the OPL (Figures 5A4–5A8, yellow arrows; Video S1). In the remaining five retinas, one animal (two eyes) evaluated at 2 weeks PI showed poor integration, another had a small graft and no migration (12 weeks PI), and another (two eyes) transplanted with NRL$^{+/EGFP}$ cells showed signs of transplant rejection.

Notably, a different pattern was seen in both eyes of the two mutant dogs with IS: in multiple areas of all four eyes, the main cell cluster was frequently seen translocated into the ONL, OPL, and INL (Figures 5A9–5A11). These integration events were observed in 0 of 10 sections evaluated in normal animals (n = 3 eyes) and between 4 of 10 and 10 of 10 sections in mutants (n = 3 eyes) (see Table S3). As with the normal retinas, smaller cell clusters were also able to migrate into the host ONL, OPL, and INL (Figures 5A11–5A14). Cells that migrated into the host ONL had extended axons and structural formations that resembled synaptic terminals. Host rod and cone ON bipolar cells extended Goα-positive dendrites toward donor cells within the ONL. This mainly occurred for PRPCs with cone-like morphology (Figures 5B2 and 5B5, white arrowheads). Occasionally protein kinase C alpha (PKCα)-positive rod bipolar cells also extended processes toward donor cells (Figures 5B1 and 5B4). In both mutant and normal retinas, the PRPCs consistently expressed the presynaptic protein human synaptophysin, suggesting their potential for forming synapses when properly integrated and polarized within the retina (Figures 5B3 and 5B6, white arrows).

Survival and integration of the donor cells: role of donor Müller cells

Donor cells isolated from day 104 to 151 ROs used in this study were composed primarily of PRPCs but also

(C) Graft rejection with infiltration of immune cells in a normal dog without IS.
(D) Complete graft rejection with rare T and B cells and massive infiltration of macrophages and microglial cells in the retina of a mutant (rcd1/*PDE6B*) dog that was not under IS.

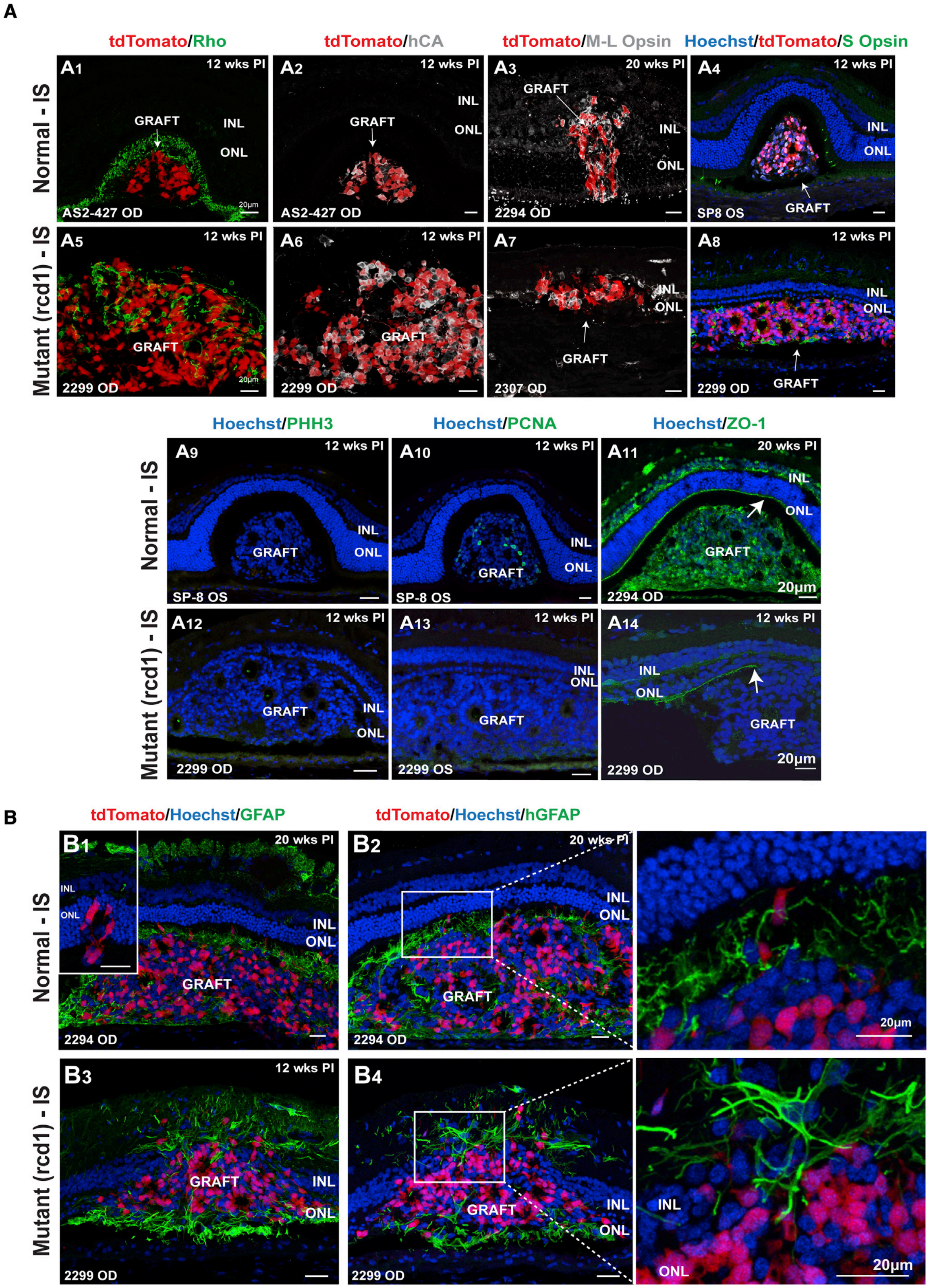

(legend on next page)

contained retinal ganglion cells, horizontal cells, amacrine cells, bipolar cells, and Müller glia (Phillips et al., 2018b). Following injection, subretinal cell clusters from these donor cell populations included PRPCs (tdTomato$^+$/Ku80$^+$), as well as Müller cells (human glial fibrillary acid protein positive [GFAP$^+$]) and bipolar cells (PKCα$^+$/Ku80$^+$ and Goα$^+$/Ku80$^+$). The donor Müller cells in normal animals under IS mainly surrounded the transplant, while endogenous canine GFAP expression was restricted to astrocytes within the NFL (Figures 4B1 and 4B2). While the glial cells within the donor tissue might promote cell survival, glial scarring around transplants in normal retinas has been observed (Aboualizadeh et al., 2020). Together, these findings suggest that formation of a glial scar encapsulating the graft likely impedes optimal cell migration and integration in animals with intact OLM. In support, smaller donor cell clusters that were not surrounded by GFAP-positive material more frequently integrated into the host retina (Figure 4B1, inset).

In rcd1/*PDE6B* mutant dogs, widespread mild GFAP upregulation in host Müller glial cells was consistent with ongoing neurodegeneration. However, in contrast with normal dogs, glial scars enclosing the graft were not observed at this stage (Figures 4B3 and 4B4). Moreover, reactive host Müller glia did not appear to prevent migration or anatomic integration of the grafted tissue in the mutant microenvironment. Interestingly, in mutant animals, donor Müller cells also extended processes and connected host and donor tissues (Figure 4B4), suggesting that they may serve as a conduit for donor cell migration or integration into the host retina.

Validation of a new surgical approach to optimize delivery of cell suspensions to the SRS

Having observed in all dogs a variable amount of cells refluxing into the vitreous at the time of the bolus subretinal injection, we expanded this study to test a novel "five-step" subretinal delivery approach (Video S2; supplemental experimental procedures S1). Although the need for a three-port pars plana vitrectomy followed by removal of the hyaloid membrane extended the duration of the surgery, the preformation of a BSS subretinal bleb that was deflated before injecting the cell suspension drastically reduced the amount of cell suspension that refluxed into the vitreal cavity. This optimized approach provides a way of delivering to the SRS the intended dose of cell suspension and reduces the risk of inducing proliferative vitreoretinopathy.

DISCUSSION

Transplantation of PSC-derived RPE cells for the treatment of AMD and Stargardt's macular dystrophy has now moved into clinical trials (Wang et al., 2020). In contrast, progress with photoreceptor replacement still faces a number of challenges, including optimizing uniform delivery of a large number of donor cells, improving long-term survival of donor photoreceptors, promoting sufficient integration into the host retina, and establishing functional synaptogenesis to enable recovery of visual function (Ludwig and Gamm, 2021). In addition, the avoidance of immune rejection of allogeneic transplants needs further optimization before clinical trials can be initiated (Zhang et al., 2021). To address these issues, we have developed longitudinal multimodal imaging to evaluate the effect of a triple-drug IS regimen on cell survival and integration of hESC-PRPCs following surgical delivery to the SRS of both normal dogs and dogs with advanced IRD. This has enabled further evaluation of the impact of the host's degenerating retinal environment on PRPC migration, differentiation, and structural integration.

Surgical delivery of cells to the SRS

We successfully delivered cells in the canine SRS using a simple surgical procedure (bolus manual injection without prior vitrectomy). Notably, vitreal reflux is common when injecting cells in the SRS (Aboualizadeh et al., 2020) because a larger retinotomy is formed as a result of the need for a wider gauge subretinal canula for cell delivery. As suggested in previous work performed in non-human primates (NHPs), the cells remaining in the vitreous can potentially affect vision and post-operative retinal imaging (Aboualizadeh et al., 2020). Although such findings suggest that vitreal backflow of donor cells should be minimized to

Figure 4. Characterization of the donor cell graft in the canine subretinal space

(A) Transplanted tdTomato$^+$ PRPCs mature to express rhodopsin (Rho; A1 and A5), human cone arrestin (hCA; A2 and A6), canine M/L opsin (A3 and A7), and S opsin (A4 and A8). This was seen in six of the eight normal and in all the mutant eyes (n = 4 eyes) under IS; representative images per group are illustrated. Rare cells positive for proliferating cell nuclear antigen (PCNA; A10 and A13) but negative for phospho-histone H3 (PPH3, A9 and A12) were noted in three out of eight normal eyes under IS, and integration into the host retina was seen at sites of OLM disruption stained with zonula occludens-1 (ZO-1) antibodies (A11 and A14 white arrows) only in mutant animals under IS.

(B) Immunostaining with a glial fibrillary acid protein (GFAP) antibody (B1 and B3) that recognizes both canine and human GFAP, as well as with a human-specific GFAP (hGFAP) antibody (B2 and B4), illustrates the formation of a glial scar around the transplant in all the normal eyes, whereas this did not occur in mutant eyes.

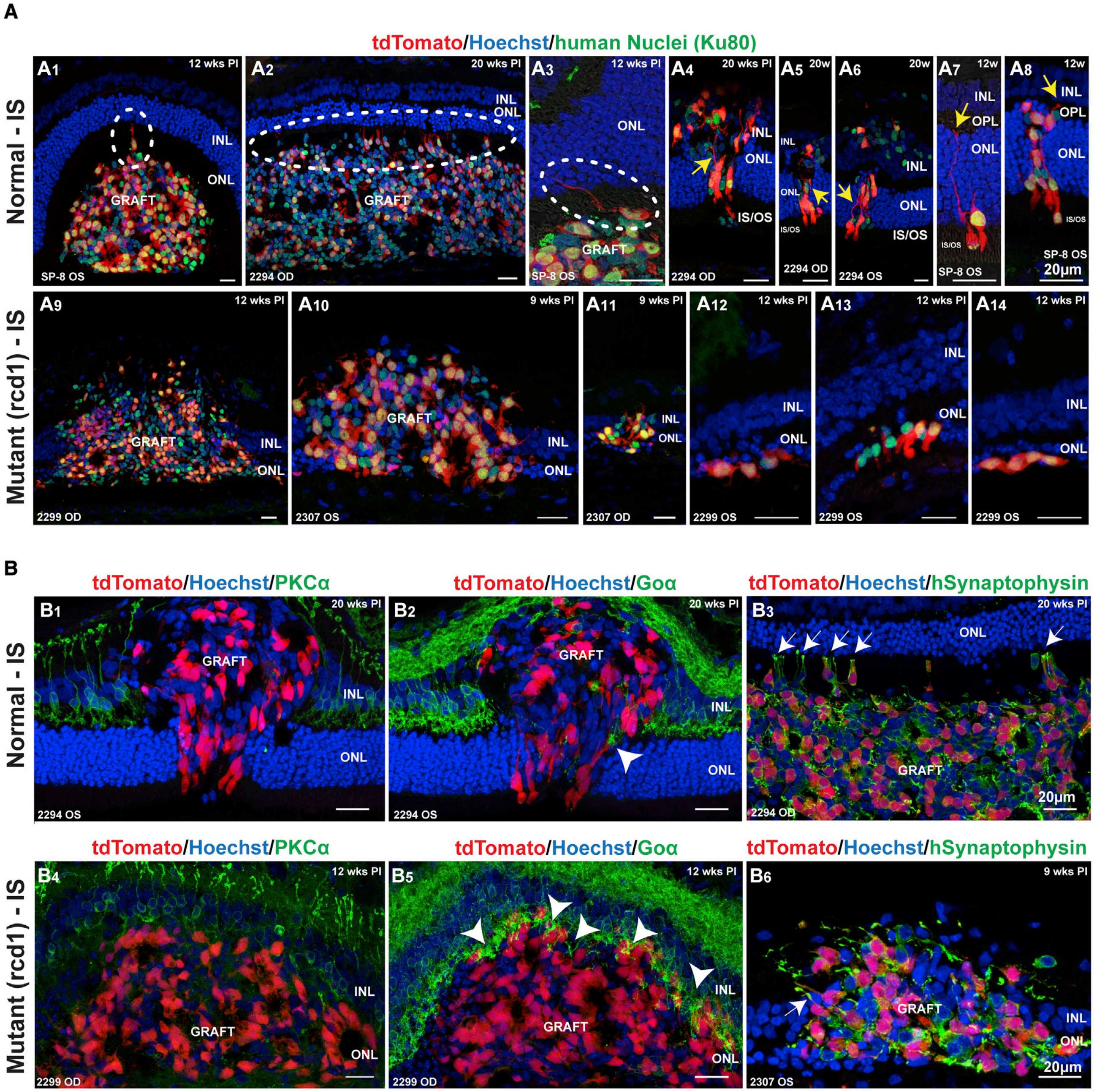

Figure 5. Donor cell integration in normal and mutant canine host retinas and development of synaptic structures

(A) In three of eight eyes from normal animals under IS, some rare donor cells extended processes into the host retina from their subretinal location (A1–A3, white dashed oval). In these same eyes, smaller cell aggregates were able to translocate their nuclei into the donor ONL (A4–A8), with an elongated photoreceptor-like morphology, including inner segment formation and neurite extension toward the donor OPL (yellow arrows). In all the rcd1/*PDE6B* mutant animals under IS, there was increased integration of the donor cell cluster within the host retinal layers (A9–A11), with individual cells seen in the host ONL (A12–A14).

(B) Rare anatomically integrated donor cells in three of eight eyes from normal animals under IS showed only minimal potential for structural synapses with few PKCα-positive rod bipolar cells (B1) and Goα-positive ON bipolar cells (B2, white arrowhead). Non-integrated donor photoreceptors that remained in the subretinal space extended neurites into the host retina ending in synaptophysin-positive pedicle-like structures that were unable to penetrate the host's OLM (B3, white arrows). In all the rcd1/*PDE6B* mutant animals under IS, when the donor cell cluster was integrated into the host ONL, rod bipolar cells (B4) and ON bipolar cells (B5, white arrowheads) were seen extending dendrites toward the grafted cells. Donor photoreceptors extended axons into the host retina expressing synaptophysin (B6, white arrow).

prevent formation of epiretinal membranes that can lead to secondary retinal detachment, the presence of refluxed cells in the vitreous of canine eyes in our studies was not associated with post-operative complications up to 22 weeks PI. However, to improve the translational value of using canine models to evaluate photoreceptor transplantation, we developed and validated a new five-step surgical delivery approach that considerably reduces vitreal reflux and thus increases the safety profile of this procedure.

Although the transvitreal approach for subretinal injection has been used in several human clinical trials of stem/progenitor cell transplantation (Wang et al., 2020), we found it was successful but suboptimal for hESC-PRPC delivery in the canine SRS. Specifically, we consistently observed an uneven distribution of cells within the SRS, thus limiting its potential therapeutic benefits. The heterogeneous distribution is most likely due to gravitational effects, because the animal's head is positioned upright immediately after surgery (Aboualizadeh et al., 2020). In clinical trials, such effects could be mitigated by post-operative bedrest and/or administration of carbonic anhydrase inhibitors that accelerate subretinal fluid resorption (Wolfensberger, 1999). Alternatively, the use of biocompatible scaffolds or biomaterials that provide a structural matrix may optimize donor cell distribution and polarization (Lee et al., 2021).

Multimodal imaging of donor cells and host retina is critical to monitoring graft survival and detecting early signs of transplant rejection

Visualization of fluorescent PRPCs in the live animal allowed us to differentiate them from host cells, thus improving on prior techniques in which non-fluorescent donor cells could be followed only by OCT or detected by histology after termination. Although this approach did not offer single-cell resolution such as that achieved with fluorescent adaptive optics scanning laser ophthalmoscopy (FAOSLO) (Aboualizadeh et al., 2020), this simple imaging technique still enabled longitudinal tracking of the same cell cluster to assess survival and integration into the host retina. In addition, we found that the use of *en face* OCT improved our ability to observe grafts in the SRS, further enhancing the detection of small clusters of donor cells that were not clearly seen on *en face* views with fundus photography or cSLO.

Consistent with previous studies (West et al., 2010), we observed two temporal patterns of donor cells loss: an early reduction in the number of grafted cells within the first week of transplantation that was independent of IS status and a delayed rejection of the graft, seen in those dogs not receiving IS. Although the cause of the initial cell loss is not known, it could potentially be driven by an early innate immune response (Kennelly et al., 2017), cell damage during processing of the ROs or surgical injection (Jager et al., 2016), as well as anoikis or apoptosis as a result of loss of cellular adhesion (Scruggs et al., 2019).

In the animals that were not under IS, there was a slight increase in graft volume in the normal dogs (20% at 12 weeks PI) and a marked decrease in the mutants (71% at 12 weeks PI). However, in all cases, this was accompanied by a drastic decrease in donor cells, as assessed by decreased donor cell fluorescence. In a previous study of RPE graft rejection in the SRS of NHP, extinction of donor cell fluorescence was a key finding indicating poor outcome, because a mononuclear inflammatory infiltrate in the SRS of these retinas could be mistaken for transplanted cells when imaging by OCT (McGill et al., 2018). Thus, multimodal retinal imaging that combines fundus photography (color and fluorescence), cSLO/OCT, and/or novel technologies such as FAOSLO provide the most accurate way of detecting early loss of donor cells (Aboualizadeh et al., 2020).

Interestingly, in normal dogs under IS, the graft volume in the SRS increased over time, but there was no evidence of inflammatory or proliferative cells. Reorganization of the graft, vacuolization, rosette formation, development of a glial barrier around the transplant (Aboualizadeh et al., 2020), or change in individual cell morphology/volume might have caused this increase.

Currently, there are no established clinical criteria to define transplant rejection in the SRS (Petrash et al., 2021), although some recognized signs of rejection are those associated with inflammation. These include hazy vitreous, vasculitis, retinal swelling, and retinal detachment (Petrash et al., 2021). Here we observed signs compatible with transplant rejection in animals that did not receive systemic IS, as well as in a single dog whose IS treatment was halted. The degree of clinical inflammation varied between animals, but retinal vasculitis, hazy vitreous, and retinal swelling were common in all the dogs with rejection of donor cells. These signs were first detected between 1 and 2–12 weeks posttransplantation, thus supporting the need for frequent monitoring of the treated retinas in the months that follow transplantation to detect potential early signs of rejection and provide an opportunity for adjusting the IS regimen.

Systemic immunosuppression is required for survival and differentiation of xenotransplants

Prior studies in mice and rats showed that IS is required to prevent allograft rejection in the SRS (Seiler et al., 2014; Zhu et al., 2017), a site previously considered to be immune privileged (West et al., 2010). Immunosuppression is a common approach to manage a wide variety of immune-mediated and inflammatory diseases in dogs (Whitley and Day, 2011). To target both innate and adaptive immune responses, we combined oral prednisolone, CsA,

and MMF, as well as topical anti-inflammatory medications. This protocol, which was initiated a week prior to transplantation and maintained throughout the study, was found to be both efficacious and well tolerated. The one exception was a dog that developed viral-induced papillomas, likely because its immunocompromised state rendered it susceptible to infection.

Consistent with prior studies in rodents and as previously reported in dogs (Xian and Huang, 2015), we found that systemic IS is critical for the survival of subretinally delivered hESC-PRPCs. Surprisingly, transplant rejection was still observed in one animal receiving systemic IS. This animal was injected with PRPCs where the rod precursors expressed EGFP and received a considerably larger transplant volume than achieved in other dogs because of a more efficient surgical delivery. Because EGFP is a known immunogen that can trigger an inflammatory response (Ansari et al., 2016), the increased EGFP expression in this dog may have triggered a more robust immune response when oral prednisolone was tapered down.

No evidence of cytoplasmic material transfer following transplantation of human PRPCs into the canine SRS

Several groups have previously reported that donor cells transplanted into the SRS fail to integrate in the recipient murine retina but instead engage in a process of cytoplasmic material transfer of RNA and/or protein (including fluorescent reporter proteins) with the host photoreceptors (Ortin-Martinez et al., 2017). We critically evaluated whether a similar mechanism occurred in canine retinas but did not observe any evidence of cytoplasmic material transfer between donor cells and host photoreceptors. However, because previous studies suggest that cytoplasmic material exchange is less likely to occur in a xenograft scenario than with autologous or allogenic transplants (Gonzalez-Cordero et al., 2017), future studies will be needed to evaluate its impact on transplant of allogeneic hESC-derived PRPCs into patients.

Grafted cells integrate more efficiently into degenerated retinas

The OLM, which is formed by tight junctions between photoreceptor inner segments and Müller glia (Omri et al., 2010), is a major barrier for migration of donor cells from the SRS into the host retina. Our results in normal dogs confirm findings in intact NHP and rodent retinas that show grafted cells persisting in the SRS in the absence of chemically, pharmacologically, or laser-induced ablation of the OLM integrity (Aboualizadeh et al., 2020; Pearson et al., 2010; West et al., 2008). However, consistent with enhanced donor cell integration by the progressive OLM disruption associated with photoreceptor degeneration (Waldron et al., 2018), we found that PRPC integration was enhanced in the rcd1/*PDE6B* mutant dog model.

Reactive gliosis and secondary retinal remodeling in IRDs are considered potential impediments to donor cell integration (Hippert et al., 2016). Although activation of Iba1^{+} microglia cells and GFAP immunoreactivity in canine Müller cells was seen throughout the retinas of degenerating rcd1/*PDE6B* mutant dogs, it did not prevent migration of donor cells. Although these dogs were transplanted at an advanced stage of degeneration (~2 rows of nuclei left in the ONL), there was no evidence of a disease-associated host Müller glial seal in the SRS. However, because specific aspects of degeneration in the diseased retina may differentially impact integration, identifying non-invasive methods to assess the permissive state of a degenerated retina to cell migration prior to photoreceptor transplantation may be critical for optimal patient selection.

The donor cell suspension isolated from day 104 to 151 ROs was composed primarily of PRPCs but also contained other retinal cell populations, including Müller cells, which were detected using a human-specific GFAP antibody that did not cross-react with canine GFAP. Although these co-injected human Müller cells did not prevent PRPC migration in degenerating retinas, they formed a "mesh" of glial processes around the graft in normal retinas, likely contributing to graft retention in the SRS and morphological changes by OCT imaging. In support, rare events of PRPC migration and cone differentiation into the host ONL were seen in normal retinas, which in all cases involved single or few PRPCs that were not surrounded by donor Müller cells. This suggests that in intact retinas, the glial seal formed by the donor Müller cells around the transplanted graft may be as much or more of a barrier to cell migration as the presence of an intact OLM. The lack of formation of such a structure in the transplanted rcd1/*PDE6B* retinas and the loss of integrity of the OLM suggest that the diseased retina provides an enhanced microenvironment for PRPC graft integration and potentially differentiation.

This study now describes an optimized five-step surgical approach to improve delivery of the full intended dose of cells to the SRS, yet cell distribution throughout the treated area was still found to be limited to the ventral border of the bleb as a result of gravitational deposition. The use of biodegradable scaffolds (Jung et al., 2018; Lee et al., 2021) to implant PRPCs over a larger area may circumvent this limitation, improve radial orientation of the photoreceptor cells, and favor the establishment of xenosynapses that could provide functional recovery.

Although previous studies support the potential for PRPCs to establish structural synapses (Aboualizadeh et al., 2020), few studies have shown functional recovery after xenotransplantation of human cells in retinas with

end-stage degeneration (Barnea-Cramer et al., 2016; Garita-Hernandez et al., 2019; Ribeiro et al., 2021). In the present study, enhanced integration of PRPCs within the degenerating retina was exemplified by development of pedicle-like structures, expression of the pre-synaptic protein synaptophysin, and establishment of contacts with host ON bipolar cells. These encouraging results now set the stage for functional evaluation of these xenosynapses in canine models of retinal degeneration to establish the translational potential of this therapeutic strategy.

EXPERIMENTAL PROCEDURES

Study animals

The dogs were part of a research colony kept at the University of Pennsylvania, Retinal Diseases Studies Facility. All procedures were carried out in strict accordance with the Association for Research in Vision and Ophthalmology (ARVO) Statement for the Use of Animals in Ophthalmic and Vision Research and approved by the Institutional Animal Care and Use Committee of the University of Pennsylvania (IACUC number: 803254).

A total of 10 dogs (6 males and 4 females; 5 months to 3 years of age) were used (Table 1), including 7 dogs (12 eyes) with normal retinas and 3 dogs (6 eyes) that were affected with a form of rod cone degeneration caused by a nonsense mutation in the *PDE6B* gene (rcd1/*PDE6B*). These dogs serve as a large-animal model of retinitis pigmentosa and by 29 weeks of age exhibit an advanced stage of retinal degeneration, defined by an ONL thickness (~2 rows of nuclei) that is less than 75% of that of a normal dog at the same age (Genini et al., 2013).

Preparation and transplantation of PRPCs

Three-dimensional (3D) ROs were generated from two established hESC reporter lines (WA09 *CRX*-tdTomato or WA09 *NRL*-EGFP; Wi-Cell, Madison, WI, USA) (Phillips et al., 2018a, 2018b) following previously established protocols (Capowski et al., 2019) to produce fluorescently labeled PRPCs (WA09 *CRX*-tdTomato) or rod precursors (WA09 *NRL*-EGFP). As previously characterized (Capowski et al., 2019; Phillips et al., 2018a, 2018b), about 70% of the cells in the ROs are CRX-tdTomato PRPCs. Cell preparation and subretinal delivery details are fully described in supplemental experimental procedures S1.

Pharmacological treatment

The animals were divided into two study groups: dogs that received triple anti-inflammatory/IS drugs (IS group; Figure S1A) and animals that were not under this medication (no-IS group). In those animals receiving the IS medical regimen, CsA (5–10 mg/kg twice a day; Atopica, Elanco US, Greenfield, IN, USA) and MMF (10 mg/kg twice a day; compounded at Wedgewood Village Pharmacy, Swedesboro, NJ, USA) were initiated 1 week before cell transplantation and given for the entire duration of the study. The dogs also received oral prednisolone (1 to 0.1 mg/kg once daily; Lannett Company, Philadelphia, PA, USA), initiated on the day of cell transplantation and progressively tapered down during a 12-week period PI. In those dogs that did not receive IS, no systemic anti-inflammatory or IS medication was given in the post-operative period (Figure S1B). In addition, the dogs received a perioperative regimen that differed in the IS and no-IS group (see supplemental experimental procedures S2).

Throughout the evaluation period, physical examinations and blood and urine collection were performed to assess whether the IS protocol was well tolerated by the dogs and to ensure that proper IS levels were achieved (see supplemental experimental procedures S2).

In vivo longitudinal monitoring of transplanted PRPCs using multimodal retinal imaging

On a weekly basis, the animals underwent an ophthalmic examination that included biomicroscopic slit-lamp evaluation, intraocular pressure measurement, and observation of the transplant by indirect ophthalmoscopy. At the end of the evaluation, retinal photographs were acquired with a RetCam retinal camera.

At different time points (Table 1), the animals were anesthetized as previously described for cell delivery, and a detailed analysis of the grafted cells was performed by using retinal color and fluorescence photographs, cSLO (NIR and AF modes), spectral-domain OCT, and *en face* OCT. Initially, several images were acquired with a Topcon retinal camera (Topcon Medical Systems, Paramus, NJ, USA) that was custom modified to incorporate a set of excitation (FF01-531/40-25; Semrock, Rochester, NY, USA) and emission (FF01-586/20; Semrock, Rochester, NY, USA) filters that enable the visualization of tdTomato fluorescence. Once the donor cells were localized, a Spectralis HRA/OCT2 unit (Heidelberg Engineering, Heidelberg, Germany) was used to evaluate the donor cell changes by cSLO (55-degree lens), OCT, and *en face* OCT (30-degree lens). In the animal injected with $NRL^{+/EGFP}$ hESCs, the Spectralis AF mode of the cSLO was used to identify the $EGFP^{+}$ cells, followed by OCT and *en face* OCT evaluation. The retinal blood vessels were used as a landmark to compare the same region between the different imaging modalities used. In addition, the Spectralis unit follow-up function was used to ensure longitudinal evaluation of the same region. The OCT raster scans consisted of 30 × 25 degrees (10 × 10 degrees for *en face* OCT) volumetric scans containing 61 (512 for *en face* OCT) horizontal b-scans spaced every 122 μm (11 μm for *en face* OCT).

In vivo quantification of the subretinal cell clusters over time

For further investigation of the transplanted cells features and evolution *in vivo*, the major cell cluster in each eye was longitudinally quantified. This was performed in all animals using the Spectralis designated software (HEYEX, Heidelberg Eye Explorer; Heidelberg Engineering, Franklin, MA, USA; see supplemental experimental procedures S3).

Immunohistochemical evaluation

At the end of the study, the animals were humanely euthanized with an intravenous overdose of pentobarbital sodium and phenytoin sodium (Euthasol; Virbac, Westlake, TX, USA). Immediately following death confirmation, the eyes were enucleated, fixed, and processed as previously described (Beltran et al., 2017).

The sections were stained with H&E and different IHC markers (Table S4) following protocols previously established (Beltran et al., 2017). Immunolabeled sections were examined by confocal microscopy (Leica TCS SP5; Leica Microsystems, Buffalo Grove, IL, USA), and digital images were acquired and processed using the Leica Application suite program. Additional details can be found in supplemental experimental procedures S4.

Immunohistochemical quantification of the integration events was performed in a subset of normal and mutant animals (see supplemental experimental procedures S5).

SUPPLEMENTAL INFORMATION

Supplemental information can be found online at https://doi.org/10.1016/j.stemcr.2022.06.009.

AUTHOR CONTRIBUTIONS

A.R.-G. assisted with surgery and animal care, conducted all the *in vivo* imaging of transplanted cells in the animals, performed immunohistochemical examination, and wrote the original draft. J.H.W. and D.M.G. conceived the project, acquired funding, and reviewed the manuscript. N.D. acquired and analyzed confocal microscopy images. M.J.P. and A.L.L. produced and coordinated shipment of cells for transplantation and reviewed the manuscript. S.A.S. and U.N. produced cells for transplantation. S.S. performed cryo-sectioning and immunolabeling. O.A.G. provided guidance with the immunosuppression protocol and analyzed results. G.D.A. conceived the project, provided animal resources, and reviewed and edited the manuscript. W.A.B. conceived the project, acquired funding, provided animal resources, performed transplantation surgery, supervised the project, and reviewed and edited the original draft.

ACKNOWLEDGMENTS

The authors wish to thank Dr. W. Merigan, Dr. J. McGregor, and W. Fisher (University of Rochester) for their help with modifications of the Nikon Topcon fundus camera; Lydia Melnyk for research coordination; Terry Jordan, Jacqueline Wivel, Nanci Newsom, Mayra Quiroz, Dr. Alexa Gray, Dr. Yu Sato, and the staff of the RDSF for anesthesia, surgical assistance, and animal care support; Dr. L. Jager for assistance with RO shipment coordination, and Dr. Leslie King and Tahira Akala for manuscript edits. This work was supported by NIH grants U24EY029890, R01EY06855, and F30EY031230; Fighting Blindness Canada, including philanthropic support from Donna Green and Goldie Feldman; the Foundation Fighting Blindness; Research to Prevent Blindness; the Van Sloun Fund for Canine Genetic Research; Retina Research Foundation Emmett A. Humble Distinguished Directorship of the McPherson Eye Research Institute; the Sanford and Susan Greenberg End Blindness Outstanding Achievement Prize; and the Sandra Lemke Trout Chair in Eye Research.

CONFLICTS OF INTEREST

D.M.G. is an inventor on patents related to generation of 3D ROs (US PTO no. 9,328,328) filed by the Wisconsin Alumni Research Foundation (Madison, WI, USA). D.M.G. and M.J.P. have ownership interest in and receive grant funding from Opsis Therapeutics, LLC, which has licensed the technology to generate 3D ROs. The terms of this arrangement have been reviewed and approved by the University of Wisconsin-Madison in accordance with its conflict-of-interest policies. M.J.P. and D.M.G. (Opsis Therapeutics, LLC) hold US Patent No. US9752119B2 (OV patent).

Received: February 23, 2022
Revised: June 27, 2022
Accepted: June 27, 2022
Published: July 28, 2022

REFERENCES

Aboualizadeh, E., Phillips, M.J., McGregor, J.E., DiLoreto, D.A., Jr., Strazzeri, J.M., Dhakal, K.R., Bateman, B., Jager, L.D., Nilles, K.L., Stuedemann, S.A., et al. (2020). Imaging transplanted photoreceptors in living nonhuman primates with single-cell resolution. Stem Cell Rep. *15*, 482–497. https://doi.org/10.1016/j.stemcr.2020.06.019.

Aghaizu, N.D., Kruczek, K., Gonzalez-Cordero, A., Ali, R.R., and Pearson, R.A. (2017). Pluripotent stem cells and their utility in treating photoreceptor degenerations. Prog. Brain Res. *231*, 191–223. https://doi.org/10.1016/bs.pbr.2017.01.001.

Ansari, A.M., Ahmed, A.K., Matsangos, A.E., Lay, F., Born, L.J., Marti, G., Harmon, J.W., and Sun, Z. (2016). Cellular GFP toxicity and immunogenicity: potential confounders in in vivo cell tracking experiments. Stem Cell Rev. Rep. *12*, 553–559. https://doi.org/10.1007/s12015-016-9670-8.

Barnea-Cramer, A.O., Wang, W., Lu, S.J., Singh, M.S., Luo, C., Huo, H., McClements, M.E., Barnard, A.R., MacLaren, R.E., and Lanza, R. (2016). Function of human pluripotent stem cell-derived photoreceptor progenitors in blind mice. Sci. Rep. *6*, 29784. https://doi.org/10.1038/srep29784.

Beltran, W.A., Cideciyan, A.V., Boye, S.E., Ye, G.J., Iwabe, S., Dufour, V.L., Marinho, L.F., Swider, M., Kosyk, M.S., Sha, J., et al. (2017). Optimization of retinal gene therapy for X-linked retinitis pigmentosa due to RPGR mutations. Mol. Ther. *25*, 1866–1880. https://doi.org/10.1016/j.ymthe.2017.05.004.

Bunel, M., Chaudieu, G., Hamel, C., Lagoutte, L., Manes, G., Botherel, N., Brabet, P., Pilorge, P., André, C., and Quignon, P. (2019). Natural models for retinitis pigmentosa: progressive retinal atrophy in dog breeds. Hum. Genet. *138*, 441–453. https://doi.org/10.1007/s00439-019-01999-6.

Capowski, E.E., Samimi, K., Mayerl, S.J., Phillips, M.J., Pinilla, I., Howden, S.E., Saha, J., Jansen, A.D., Edwards, K.L., Jager, L.D., et al. (2019). Reproducibility and staging of 3D human retinal organoids across multiple pluripotent stem cell lines. Development *146*, 171686. Cambridge, England. https://doi.org/10.1242/dev.171686.

Garita-Hernandez, M., Lampič, M., Chaffiol, A., Guibbal, L., Routet, F., Santos-Ferreira, T., Gasparini, S., Borsch, O., Gagliardi, G., Reichman, S., et al. (2019). Restoration of visual function by transplantation of optogenetically engineered photoreceptors. Nat. Commun. *10*, 4524. https://doi.org/10.1038/s41467-019-12330-2.

Genini, S., Beltran, W.A., and Aguirre, G.D. (2013). Up-regulation of tumor necrosis factor superfamily genes in early phases of photoreceptor degeneration. PLoS One *8*, e85408. https://doi.org/10.1371/journal.pone.0085408.

Gonzalez-Cordero, A., Kruczek, K., Naeem, A., Fernando, M., Kloc, M., Ribeiro, J., Goh, D., Duran, Y., Blackford, S.J., Abelleira-Hervas, L., et al. (2017). Recapitulation of human retinal development from human pluripotent stem cells generates transplantable populations of cone photoreceptors. Stem Cell Rep. *9*, 820–837. https://doi.org/10.1016/j.stemcr.2017.07.022.

Hippert, C., Graca, A.B., and Pearson, R.A. (2016). Gliosis can impede integration following photoreceptor transplantation into the diseased retina. Adv. Exp. Med. Biol. *854*, 579–585.

Jager, L.D., Canda, C.M.A., Hall, C.A., Heilingoetter, C.L., Huynh, J., Kwok, S.S., Kwon, J.H., Richie, J.R., and Jensen, M.B. (2016). Effect of enzymatic and mechanical methods of dissociation on neural progenitor cells derived from induced pluripotent stem cells. Adv. Med. Sci. *61*, 78–84. https://doi.org/10.1016/j.advms.2015.09.005.

Jung, Y.H., Phillips, M.J., Lee, J., Xie, R., Ludwig, A.L., Chen, G., Zheng, Q., Kim, T.J., Zhang, H., Barney, P., et al. (2018). 3D microstructured scaffolds to support photoreceptor polarization and maturation. Adv. Mater. *30*, e1803550. Deerfield Beach, Fla. https://doi.org/10.1002/adma.201803550.

Kennelly, K.P., Holmes, T.M., Wallace, D.M., O'Farrelly, C., and Keegan, D.J. (2017). Early subretinal allograft rejection is characterized by innate immune activity. Cell Transpl. *26*, 983–1000. https://doi.org/10.3727/096368917x694697.

Lee, I.K., Ludwig, A.L., Phillips, M.J., Lee, J., Xie, R., Sajdak, B.S., Jager, L.D., Gong, S., Gamm, D.M., and Ma, Z. (2021). Ultrathin micromolded 3D scaffolds for high-density photoreceptor layer reconstruction. Sci. Adv. *7*, eabf0344. https://doi.org/10.1126/sciadv.abf0344.

Ludwig, A.L., and Gamm, D.M. (2021). Outer retinal cell replacement: putting the pieces together. Transl Vis. Sci. Technol. *10*, 15. https://doi.org/10.1167/tvst.10.10.15.

McGill, T.J., Stoddard, J., Renner, L.M., Messaoudi, I., Bharti, K., Mitalipov, S., Lauer, A., Wilson, D.J., and Neuringer, M. (2018). Allogeneic iPSC-derived RPE cell graft failure following transplantation into the subretinal space in nonhuman primates. Invest Ophthalmol. Vis. Sci. *59*, 1374. https://doi.org/10.1167/iovs.17-22467.

Omri, S., Omri, B., Savoldelli, M., Jonet, L., Thillaye-Goldenberg, B., Thuret, G., Gain, P., Jeanny, J.C., Crisanti, P., and Behar-Cohen, F. (2010). The outer limiting membrane (OLM) revisited: clinical implications. Clin. Ophthalmol. *4*, 183–195. https://doi.org/10.2147/opth.s5901.

Ortin-Martinez, A., Tsai, E.L.S., Nickerson, P.E., Bergeret, M., Lu, Y., Smiley, S., Comanita, L., and Wallace, V.A. (2017). A reinterpretation of cell transplantation: GFP transfer from donor to host photoreceptors. Stem Cells *35*, 932–939. https://doi.org/10.1002/stem.2552.

Pearson, R.A., Barber, A.C., West, E.L., MacLaren, R.E., Duran, Y., Bainbridge, J.W., Sowden, J.C., and Ali, R.R. (2010). Targeted disruption of outer limiting membrane junctional proteins (Crb1 and ZO-1) increases integration of transplanted photoreceptor precursors into the adult wild-type and degenerating retina. Cell Transpl. *19*, 487–503. https://doi.org/10.3727/096368909X486057.

Petrash, C.C., Palestine, A.G., and Canto-Soler, M.V. (2021). Immunologic rejection of transplanted retinal pigmented epithelium: mechanisms and strategies for prevention. Front. Immunol. *12*, 621007. https://doi.org/10.3389/fimmu.2021.621007.

Phillips, M.J., Capowski, E.E., Petersen, A., Jansen, A.D., Barlow, K., Edwards, K.L., and Gamm, D.M. (2018a). Generation of a rod-specific NRL reporter line in human pluripotent stem cells. Sci. Rep. *8*, 2370. https://doi.org/10.1038/s41598-018-20813-3.

Phillips, M.J., Jiang, P., Howden, S., Barney, P., Min, J., York, N.W., Chu, L.F., Capowski, E.E., Cash, A., Jain, S., et al. (2018b). A novel approach to single cell RNA-sequence analysis facilitates in silico gene reporting of human pluripotent stem cell-derived retinal cell types. Stem Cells *36*, 313–324. https://doi.org/10.1002/stem.2755.

Ribeiro, J., Procyk, C.A., West, E.L., O'Hara-Wright, M., Martins, M.F., Khorasani, M.M., Hare, A., Basche, M., Fernando, M., Goh, D., et al. (2021). Restoration of visual function in advanced disease after transplantation of purified human pluripotent stem cell-derived cone photoreceptors. Cell Rep. *35*, 109022. https://doi.org/10.1016/j.celrep.2021.109022.

Scruggs, B.A., Jiao, C., Cranston, C.M., Kaalberg, E., Wang, K., Russell, S.R., Wiley, L.A., Mullins, R.F., Stone, E.M., Tucker, B.A., and Sohn, E.H. (2019). Optimizing donor cellular dissociation and subretinal injection parameters for stem cell-based treatments. Stem Cell translational Med. *8*, 797–809. https://doi.org/10.1002/sctm.18-0210.

Seiler, M.J., Aramant, R.B., Jones, M.K., Ferguson, D.L., Bryda, E.C., and Keirstead, H.S. (2014). A new immunodeficient pigmented retinal degenerate rat strain to study transplantation of human cells without immunosuppression. Graefes Arch. Clin. Exp. Ophthalmol. *252*, 1079–1092. https://doi.org/10.1007/s00417-014-2638-y.

Singh, R., Cuzzani, O., Binette, F., Sternberg, H., West, M.D., and Nasonkin, I.O. (2018). Pluripotent stem cells for retinal tissue engineering: current status and future prospects. Stem Cell Rev. Rep. *14*, 463–483. https://doi.org/10.1007/s12015-018-9802-4.

Tu, H.Y., Watanabe, T., Shirai, H., Yamasaki, S., Kinoshita, M., Matsushita, K., Hashiguchi, T., Onoe, H., Matsuyama, T., Kuwahara, A., et al. (2019). Medium- to long-term survival and functional examination of human iPSC-derived retinas in rat and primate models of retinal degeneration. EBioMedicine *39*, 562–574. https://doi.org/10.1016/j.ebiom.2018.11.028.

Waldron, P.V., Di Marco, F., Kruczek, K., Ribeiro, J., Graca, A.B., Hippert, C., Aghaizu, N.D., Kalargyrou, A.A., Barber, A.C., Grimaldi, G., et al. (2018). Transplanted donor- or stem cell-derived cone photoreceptors can both integrate and undergo material transfer in an environment-dependent manner. Stem Cell Rep. *10*, 406–421. https://doi.org/10.1016/j.stemcr.2017.12.008.

Wang, Y., Tang, Z., and Gu, P. (2020). Stem/progenitor cell-based transplantation for retinal degeneration: a review of clinical trials. Cell Death Dis. *11*, 793. https://doi.org/10.1038/s41419-020-02955-3.

West, E.L., Pearson, R.A., Barker, S.E., Luhmann, U.F., Maclaren, R.E., Barber, A.C., Duran, Y., Smith, A.J., Sowden, J.C., and Ali, R.R. (2010). Long-term survival of photoreceptors transplanted into the adult murine neural retina requires immune modulation. Stem Cells *28*, 1997–2007. https://doi.org/10.1002/stem.520.

West, E.L., Pearson, R.A., Tschernutter, M., Sowden, J.C., MacLaren, R.E., and Ali, R.R. (2008). Pharmacological disruption of the outer limiting membrane leads to increased retinal integration of transplanted photoreceptor precursors. Exp. Eye Res. *86*, 601–611. https://doi.org/10.1016/j.exer.2008.01.004.

Whitley, N.T., and Day, M.J. (2011). Immunomodulatory drugs and their application to the management of canine immune-mediated disease. J. Small Anim. Pract. *52*, 70–85. https://doi.org/10.1111/j.1748-5827.2011.01024.x.

Wolfensberger, T.J. (1999). The role of carbonic anhydrase inhibitors in the management of macular edema. Doc Ophthalmol. *97*, 387–397. https://doi.org/10.1023/a:1002143802926.

Xian, B., and Huang, B. (2015). The immune response of stem cells in subretinal transplantation. Stem Cell Res. Ther. *6*, 1–7. https://doi.org/10.1186/s13287-015-0167-1.

Zhang, C.J., Ma, Y., and Jin, Z.B. (2021). The road to restore vision with photoreceptor regeneration. Exp. Eye Res. *202*, 108283. https://doi.org/10.1016/j.exer.2020.108283.

Zhu, J., Cifuentes, H., Reynolds, J., and Lamba, D.A. (2017). Immunosuppression via loss of IL2rγ enhances long-term functional integration of hESC-derived photoreceptors in the mouse retina. Cell Stem Cell *20*. 374–384.e375. https://doi.org/10.1016/j.stem.2016.11.019.

Stem Cell Reports, Volume *17*

Supplemental Information

Systemic immunosuppression promotes survival and integration of subretinally implanted human ESC-derived photoreceptor precursors in dogs

Ana Ripolles-Garcia, Natalia Dolgova, M. Joseph Phillips, Svetlana Savina, Allison L. Ludwig, Sara A. Stuedemann, Uchenna Nlebedum, John H. Wolfe, Oliver A. Garden, Arvydas Maminishkis, Juan Amaral, Kapil Bharti, David M. Gamm, Gustavo D. Aguirre, and William A. Beltran

SUPPLEMENTAL FIGURES

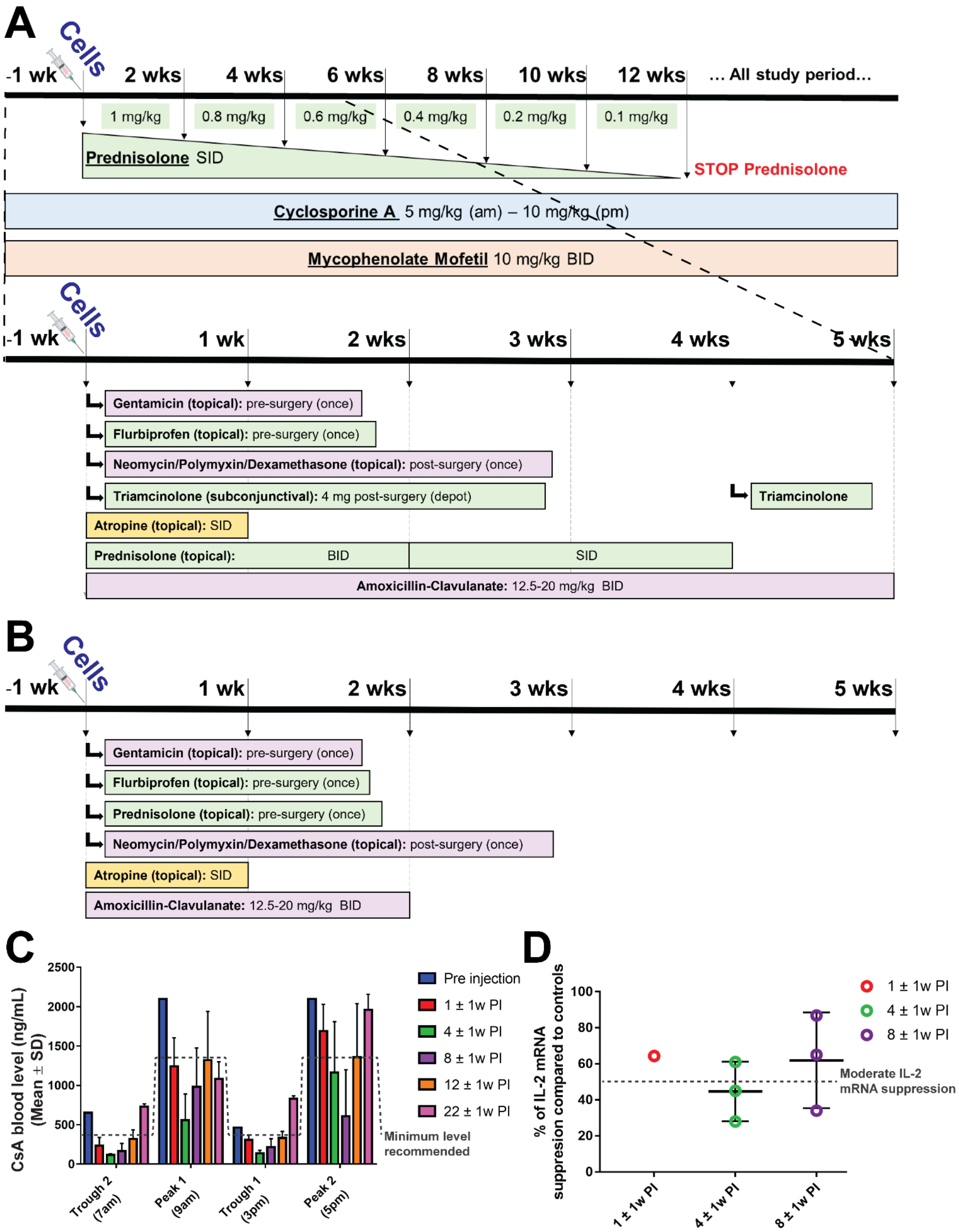
A
Cells
-1 wk
2 wks
4 wks
6 wks
8 wks
10 wks
12 wks
... All study period...
1 mg/kg
0.8 mg/kg
0.6 mg/kg
0.4 mg/kg
0.2 mg/kg
0.1 mg/kg
Prednisolone SID
STOP Prednisolone
Cyclosporine A 5 mg/kg (am) – 10 mg/kg (pm)
Mycophenolate Mofetil 10 mg/kg BID
1 wk
2 wks
3 wks
4 wks
5 wks
Gentamicin (topical): pre-surgery (once)
Flurbiprofen (topical): pre-surgery (once)
Neomycin/Polymyxin/Dexamethasone (topical): post-surgery (once)
Triamcinolone (subconjunctival): 4 mg post-surgery (depot)
Triamcinolone
Atropine (topical): SID
Prednisolone (topical):
BID
SID
Amoxicillin-Clavulanate: 12.5-20 mg/kg BID
B
Prednisolone (topical): pre-surgery (once)
C
CsA blood level (ng/mL) (Mean ± SD)
Trough 2 (7am)
Peak 1 (9am)
Trough 1 (3pm)
Peak 2 (5pm)
Pre injection
1 ± 1w PI
4 ± 1w PI
8 ± 1w PI
12 ± 1w PI
22 ± 1w PI
Minimum level recommended
D
% of IL-2 mRNA suppresion compared to controls
Moderate IL-2 mRNA suppression
1 ± 1w PI
4 ± 1w PI
8 ± 1w PI

Supplemental Figure S1. Medication regimen given to dogs that received a xenotransplant of hESC-PRPCs. A) Triple-drug immunosuppression (IS) protocol regimen (upper timeline). These animals also received a complementary antibiotic and anti-inflammatory regimen in the initial weeks after surgery (lower timeline). **B**) Antibiotic and anti-inflammatory treatment given in the weeks following surgical cell delivery, in the dogs that did not receive systemic IS. **Pharmacokinetic and pharmacodynamic monitoring of Cyclosporine A (CsA) throughout the study. C**) Pharmacokinetic evaluation of CsA blood levels. The black dashed line marks the minimum recommended by the laboratory to control immune-mediated diseases. **D**) Pharmacodynamic analysis of IL-2 mRNA inhibition, expressed as a percentage of inhibition in comparison with a cohort of normal dogs used by the laboratory. The grey dotted line illustrates 50% of mRNA inhibition, which is considered moderate immune-suppression. Sample collection was not possible at all the time-points due to laboratory intermittent closure. Data are represented as mean ± SD.

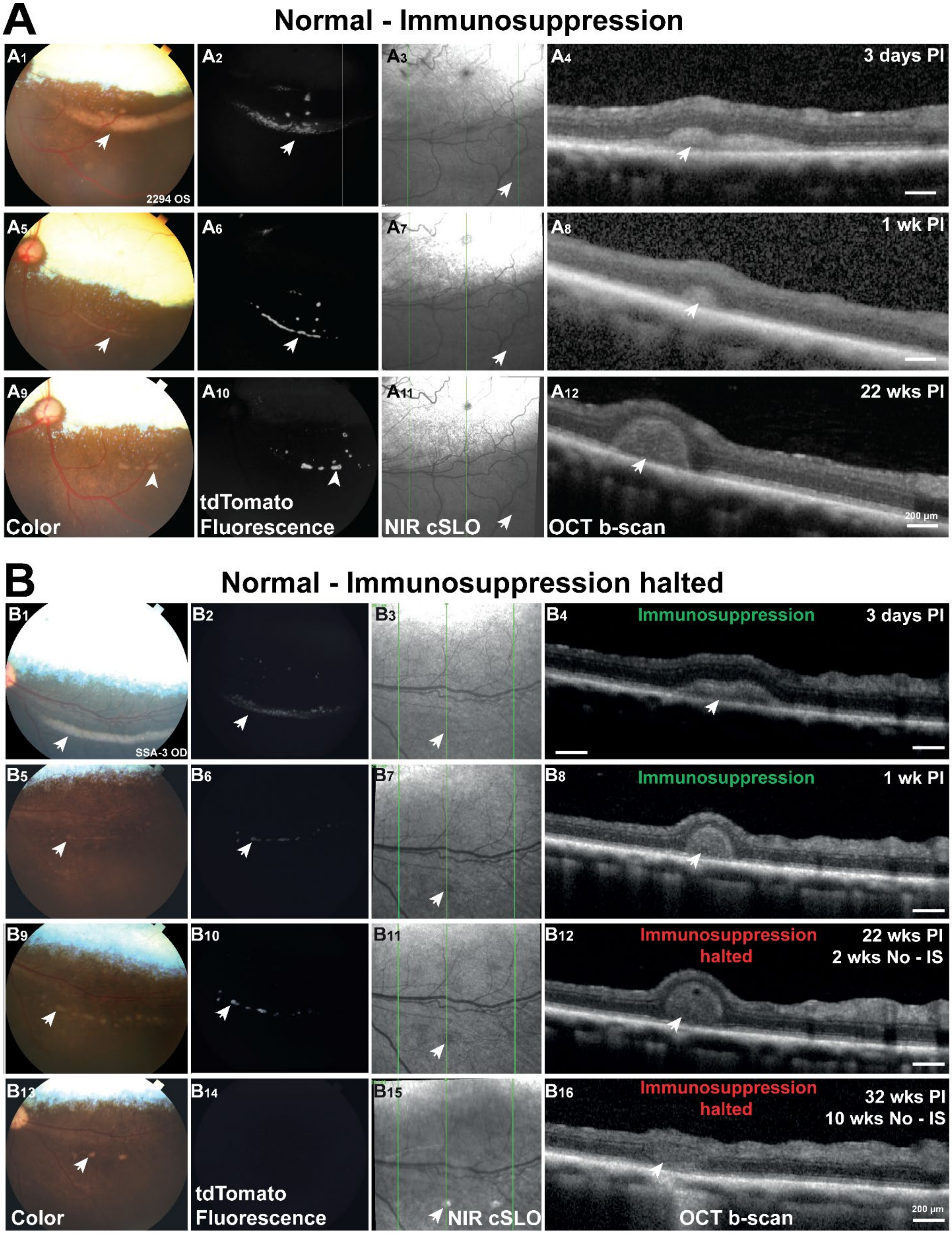

Supplemental Figure S2. Related to Figure 2. Significant donor cell graft loss occurs in the initial week after cell transplantation in the canine subretinal space. Color (A1, A5, A9), fluorescence (A2,

A6, A10), NIR cSLO (A3, A7, A11), and OCT b-scan (A4, A8, A12) retinal imaging acquired 3 days (A1-4), 1 week (A5-8), and 22 weeks (A9-12) post injection (PI). Note that there was an initial cell loss seen between 3 days (A1, A4) and 1 week (A5, A8) PI, but then there was no major loss up to 22 weeks (A5, A9); instead, a reorganization of the graft structure is observed by OCT (A12). As a result of this graft remodeling, the graft area was smaller (A10) but its height is markedly increased (A12). OS: left eye. **Transplant rejection after halting systemic immunosuppression.** Color (B1, B5, B9 and B13), fluorescence (B2, B6, B10 and B14), NIR cSLO (B3, B7, B11 and B15), and OCT b-scan (B4, B8, B12 and B16) retinal imaging acquired three days (B1-4), 1 week (B5-8), 22 weeks (B9-12) and 32 weeks (B13-16) after subretinal cell delivery. In this animal, systemic immunosuppression was halted at 20 weeks after cell transplantation. Two weeks following medication withdrawal, there was an increase in cell mass (B12). Both fluorescence and cells visible by OCT were lost by 10 weeks after medication was halted (B14 and B16). OD: right eye.

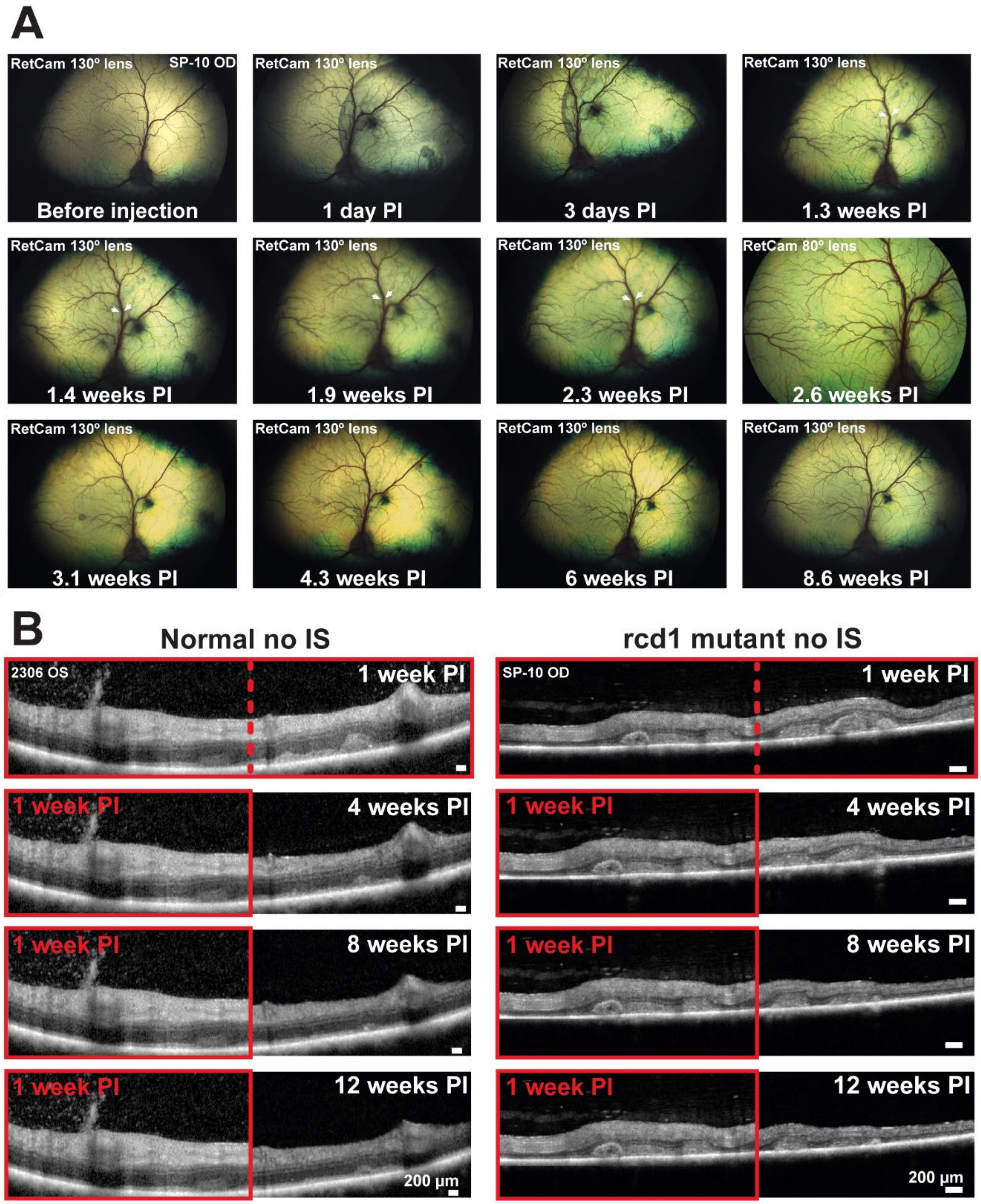

Supplemental Figure S3. Ophthalmoscopic and OCT signs of transplant rejection. Related to figure 2. A) Funduscopic signs of retinal vasculitis (vessel tortuosity, vascular enlargement, and perivascular cuffing) in a normal dog that was not under immunosuppression (IS) regimen. White arrows point at a representative vessel with perivascular cuffing. **B**) OCT alterations (mild retinal swelling,

punctate hyperreflective vitreal foci) in a normal and a mutant dog that were not under IS. Signs were visible at 1 week after transplantation and progressively decreased over the 12 weeks follow-up period. Left half of each panel shows a portion (red box) of the OCT b-scan at 1-week post-injection. OD: right eye; OS: left eye.

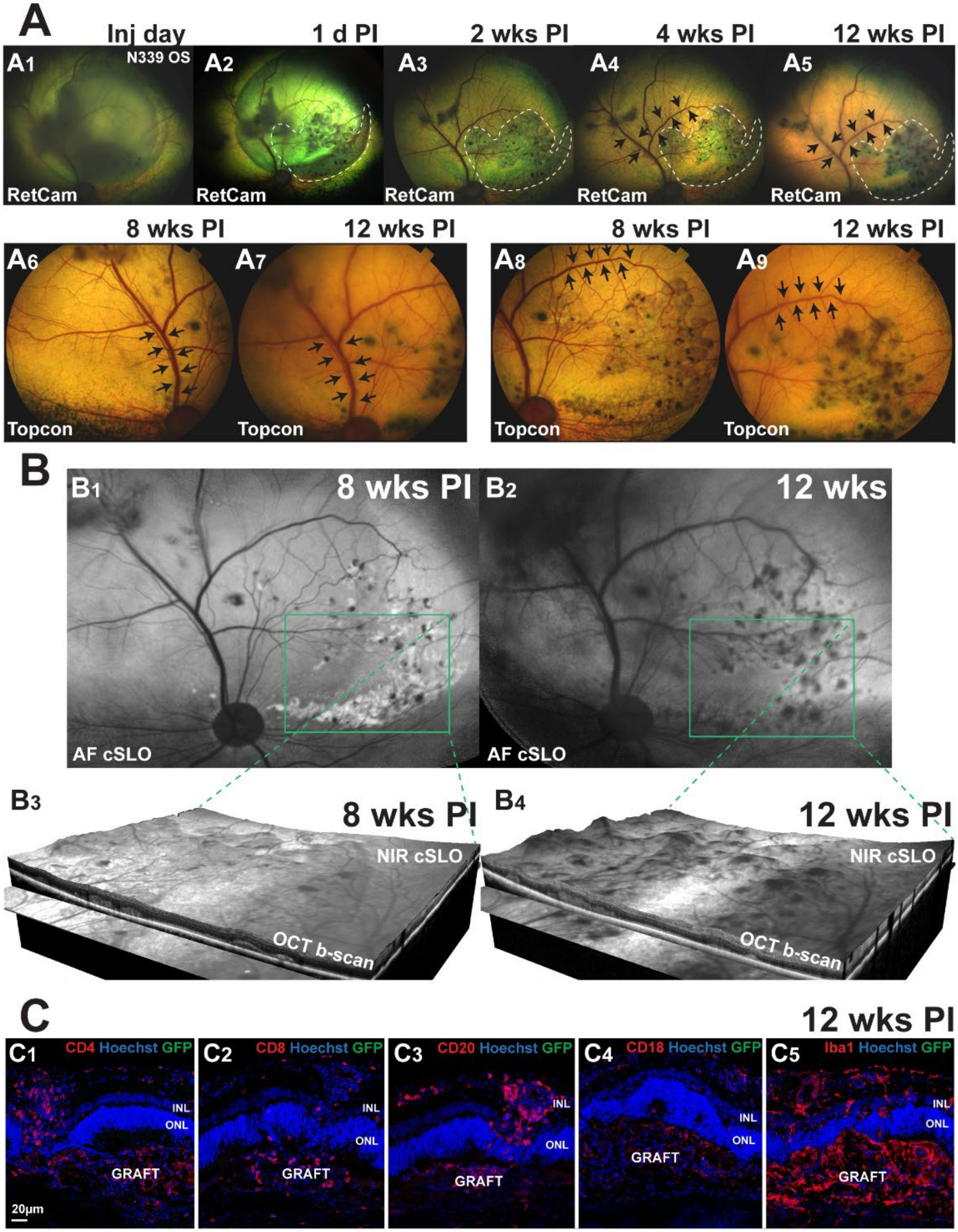
A
Inj day
1 d PI
2 wks PI
4 wks PI
12 wks PI
A1
N339 OS
A2
A3
A4
A5
RetCam
8 wks PI
12 wks PI
8 wks PI
12 wks PI
A6
A7
A8
A9
Topcon
B
B1
8 wks PI
B2
12 wks
AF cSLO
B3
8 wks PI
B4
12 wks PI
NIR cSLO
OCT b-scan
C
12 wks PI
C1 CD4 Hoechst GFP
C2 CD8 Hoechst GFP
C3 CD20 Hoechst GFP
C4 CD18 Hoechst GFP
C5 Iba1 Hoechst GFP
INL
ONL
GRAFT
20µm

Supplemental Figure S4. Transplant rejection in one normal dog that received systemic immunosuppression regimen. A) RetCam (A_1-A_5) and Topcon color (A_6-A_9) fundus photographs illustrating the subretinal cell mass (white dashed line). The retinal vessels were enlarged and their borders poorly delineated indicating vasculitis at 12 weeks post transplantation (A_5, A_7 and A_9, black arrows) in comparison with the previous time-point evaluated, 8 weeks post injection (A_4, A_6 and A_8, black arrows). The vitreous haziness prevented sharp visualization of the fundus, indicative of vitritis (A_5, A_7 and A_9). **B**) Autofluorescence cSLO imaging confirmed the presence of GFP^+ cells at 8 weeks post-delivery (B_1) but these were no longer visible by 12 weeks post injection (B_2). Generalized retinal swelling was also detected at 12 weeks (B_3) in comparison to 8 weeks post injection (B_4). **C**) A mixed inflammatory cell infiltrate was seen infiltrating the graft and was dominated by microglial cells (C_5). Helper and cytotoxic T-cells ($C_{1\text{-}2}$), B-cells (C_3), and macrophages (C_4) were also present. No donor cells were seen in the subretinal space, and a severe disruption of retinal structure was present. OS: left eye.

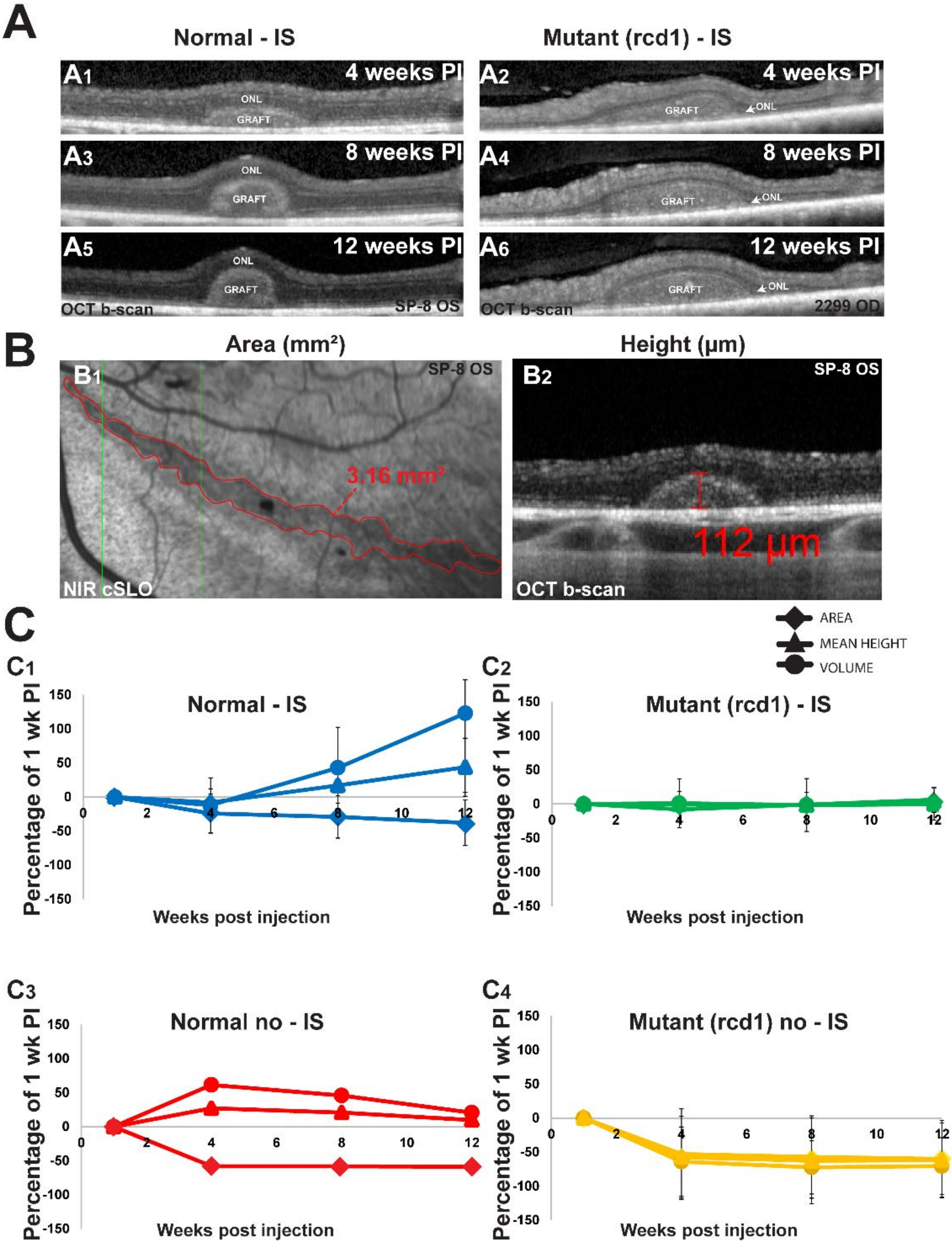

A
Normal - IS
Mutant (rcd1) - IS
A1
4 weeks PI
ONL
GRAFT
A2
4 weeks PI
GRAFT
ONL
A3
8 weeks PI
ONL
GRAFT
A4
8 weeks PI
GRAFT
ONL
A5
12 weeks PI
ONL
GRAFT
OCT b-scan
SP-8 OS
A6
12 weeks PI
GRAFT
ONL
OCT b-scan
2299 OD
B
Area (mm²)
Height (µm)
B1
SP-8 OS
3.16 mm²
NIR cSLO
B2
SP-8 OS
112 µm
OCT b-scan
C
AREA
MEAN HEIGHT
VOLUME
C1
Normal - IS
Percentage of 1 wk PI
Weeks post injection
C2
Mutant (rcd1) - IS
Percentage of 1 wk PI
Weeks post injection
C3
Normal no - IS
Percentage of 1 wk PI
Weeks post injection
C4
Mutant (rcd1) no - IS
Percentage of 1 wk PI
Weeks post injection

Supplemental Figure S5. Longitudinal characterization of the subretinal graft in normal and mutant animals with and without systemic immunosuppression. A) Representative OCT b scans from normal and mutant dogs under systemic immunosuppression (IS) illustrate morphological changes of the subretinal mass at 4 weeks ($A_{1\text{-}2}$), 8 weeks ($A_{3\text{-}4}$) and 12 weeks post injection ($A_{5\text{-}6}$). **B**) Area (B_1) and height (B_2) of the transplant measured respectively on the NIR cSLO image and OCT b scans. **C**) Changes in cell mass area, height, and volume as a function of time post injection (results are compared to values at 1 week PI). Data are represented as mean ± SD for each group: (C_1) Normal IS (n = 9 eyes, 5 dogs), (C_2) mutant IS (n = 4 eyes, 2 dogs at 4 weeks; n = 3 eyes, 2 dogs at 8 weeks; and n = 2 eyes, 1 dog at 12 weeks post injection), (C_3) normal no-IS (n = 1 eye) and (C_4) mutant no-IS (n = 2 eyes, 1 dog). OD: right eye; OS: left eye.

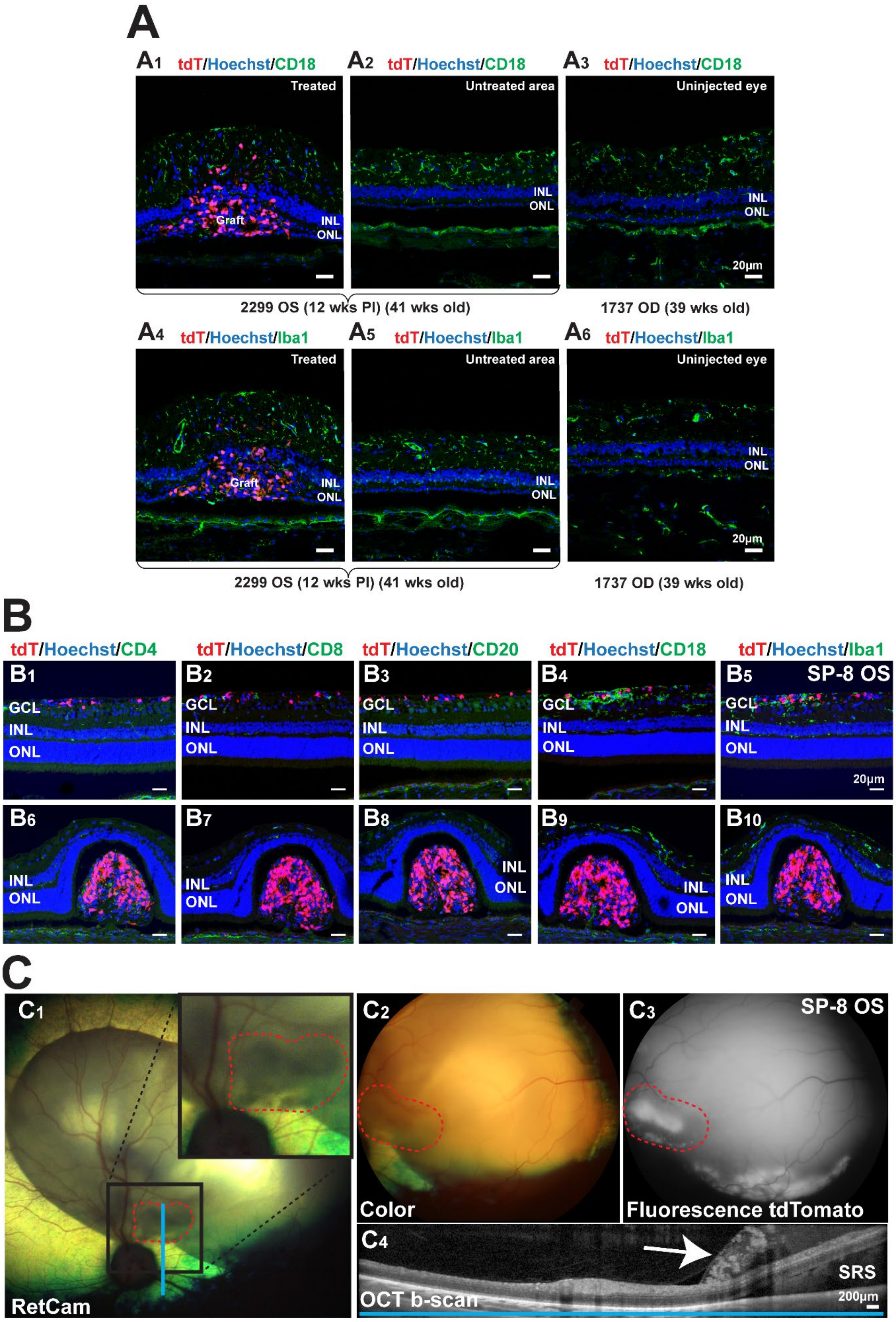
A
A1 tdT/Hoechst/CD18
Treated
Graft
INL
ONL
A2 tdT/Hoechst/CD18
Untreated area
INL
ONL
A3 tdT/Hoechst/CD18
Uninjected eye
INL
ONL
20µm
2299 OS (12 wks PI) (41 wks old)
1737 OD (39 wks old)
A4 tdT/Hoechst/Iba1
Treated
Graft
INL
ONL
A5 tdT/Hoechst/Iba1
Untreated area
INL
ONL
A6 tdT/Hoechst/Iba1
Uninjected eye
INL
ONL
20µm
2299 OS (12 wks PI) (41 wks old)
1737 OD (39 wks old)
B
tdT/Hoechst/CD4
tdT/Hoechst/CD8
tdT/Hoechst/CD20
tdT/Hoechst/CD18
tdT/Hoechst/Iba1
B1 GCL INL ONL
B2 GCL INL ONL
B3 GCL INL ONL
B4 GCL INL ONL
B5 SP-8 OS GCL INL ONL 20µm
B6 INL ONL
B7 INL ONL
B8 INL ONL
B9 INL ONL
B10 INL ONL
C
C1
RetCam
C2
Color
C3 SP-8 OS
Fluorescence tdTomato
C4
SRS
OCT b-scan
200µm

Supplemental Figure S6. Immunohistochemical characterization of CD18+ and Iba1+ cell mediated activation in the inner retina of rcd1/*PDE6B* mutant dogs. Related to figure 3. A) CD18+ cells infiltrating the inner retina of treated (A1) and untreated (A2) areas of the same eye, as well as in age-matched uninjected rcd1/*PDE6B* mutant eye (A3). Iba+ cells in the inner retina of treated (A4) and untreated (A5) areas of the same eye, as well as in an age-matched uninjected rcd1/*PDE6B* mutant eye (A6). Donor cells expressing tdTomato are labeled with red in all panels. OD: right eye; OS: left eye. **Donor cells unintentionally delivered under the inner limiting membrane trigger a more robust inflammatory response than those injected in the subretinal space in the same eye. B**) Located in the inner retina, numerous inflammatory cells consisting primarily of macrophages (CD18) and microglial cells (Iba1) were seen surrounding the donor cells (B1-5). In the same retinal section, inflammatory markers did not show evidence of infiltration of cells directed towards the subretinal transplant (B6-10). **C**) Cell mass (C1-3, red dotted line) found to have been injected under the inner limiting membrane (C4). The OCT b-scan in (C4) is extracted from the area marked with a blue line in (C1). Donor cells expressing tdTomato are labeled with red in all panels. OS: left eye; GCL: ganglion cell layer; INL: inner nuclear layer; ONL: outer nuclear layer.

SUPPLEMENTAL TABLES

Supplemental Table S1. Summary of the longitudinal monitoring by non-invasive imaging of donor cell survival following transplantation.

	Dog ID	Eye	4 ± 1 weeks PI		8 ± 1 weeks PI		12 ± 1 weeks PI		22 ± 1 weeks PI		32 ± 1 weeks PI	
			Fluo	OCT	Fluo	OCT	Fluo	OCT	Fluo	OCT	Fluo	OCT
**Normal IS**	**SP8**	OS	=	=	=	=	=	=				
	**AS2-427**	OD	=	=	=	↓	=	=				
		OS	=	↓	=	↓	=	=				
	**N339**	OD	=	↓	=	=	↓↓↓	↑↑↑				
		OS	=	↓	=	=	↓↓↓	↑↑↑				
	**2294**	OD	=	=	=	=	=	=	=	=		
		OS	=	=	=	=	=	=	=	=		
	**SSA-3**	OD	=	=	=	=	=	=				
		OS	=	=	=	=	=	=				
**Normal No-IS**	**SSA-3** *IS stopped at 20 wks PI*	OD							↓	↑↑↑	↓↓↓	↓↓↓
		OS							↓	↑↑↑	↓↓↓	↓↓↓
	**SP-10**	OD	↓↓↓	↓↓↓	↓↓↓	↓↓↓	↓↓↓	↓↓↓				
**Mutant (rcd1/*PDE6B*) IS**	**2299**	OD	=	=	=	=	=	=				
		OS	=	=	=	=	=	=				
	**2307**	OD	=	↓	=	=						
		OS	=	↓								
**Mutant (rcd1/*PDE6B*) No-IS**	**2306**	OD	↓↓↓	↓↓↓	↓↓↓	↓↓↓	↓↓↓	↓↓↓				
		OS	↓↓↓	↓↓↓	↓↓↓	↓↓↓	↓↓↓	↓↓↓				

IS: immunosuppression; OD: right eye; OS: left eye; Fluo: fluorescence observed by fundus photography and compared to the previous time-point; OCT: volume of cells observed by Optical Coherence Tomography compared to the previous time-point; =: stable; ↓: minimal decrease; ↓↓↓: severe decrease; ↑↑↑: severe increase.

Supplemental Table S2. Summary of immunohistochemistry findings in all processed eyes.

Finding on IHC				Cellular inflammation	Grafted cells displaced into host retina	Potential for synapses	Predominant cone differentiation	Graft proliferation		Cytoplasmic exchange
	**Dog ID**	**Eye**	**Weeks PI**	**CD4, CD8, CD20, CD18, Iba1**	**Ku80**	**PKCα, Goα, Synaptophysin**	**hArrestin3**	**PCNA**	**PHH3**	**$Ku80^-$ / tdTomato or GFP^+**
**Normal IS**	**SP8**	OS	11.6	-	+	+	+	+	-	-
	**SSA-1**	OD	1.6	-	-	-	+	+	NP	-
	**SSA-1**	OS	1.6	-	-	-	+	+	-	-
	**AS2-427**	OD	11.7	-	-	-	+	-	-	-
	**N339**	OD	12.1	+++	-	-	NP	-	-	-
	**N339**	OS	12.1	+++	-	-	NP	-	-	-
	**2294**	OD	20.6	-	+	+	+	-	-	-
	**2294**	OS	20.6	-	+	+	+	-	-	-
**Normal NO-IS**	**SSA-3**	OD	31.6	+++	-	-	NP	+	-	-
	**SP-10**	OD	11.6	+++	-	-	NP	NP	NP	-
**Mutant IS**	**2299**	OD	12.1	-	+++	+	+	-	NP	-
	**2299**	OS	12.1	-	+++	+	+	-	-	-
	**2307**	OD	9	-	+++	+	+	NP	NP	-
	**2307**	OS	9	-	+++	+	+	NP	NP	-
**Mutant NO-IS**	**2306**	OD	12.1	+++	-	-	NP	NP	NP	-
	**2306**	OS	12.1	+++	-	-	NP	-	-	-

IS: immunosuppression; NO-IS: no immunosuppression; OD: right eye; OS: left eye; PI: post injection; NP: not performed; +: minor; +++: severe; -: not detected.

Supplemental Table S3. Quantification of integration events in a cohort of animals.

Group	Eye (dog ID)	Weeks post injection	Frequency of integration	Number of cells injected (Aggr CRX$^{/tdTomato+}$)
Normal IS	Left eye (SP8)	11.6	0/10	4 million in 150 µL
Normal IS	Right eye (AS2-427)	11.7	0/10	2.7 million in 100 µL
Normal IS	Left eye (2294)	20.6	0/10	4 million in 150 µL
Mutant IS	Right eye (2299)	12.1	10/10	4 million in 100 µL
Mutant IS	Left eye (2299)	12.1	4/10	4 million in 100 µL
Mutant IS	Right eye (2307)	9	7/10	2 million in 100 µL

IS: immunosuppression; NO-IS: no immunosuppression.

Supplemental Table S4. List of primary antibodies used for immunohistochemistry.

Antigen	Host	Source, Catalog #	Working concentration	Normal Location
Ku80	Mouse monoclonal IgG1	(STEM101) Takara Bio, Y40400	1:500	Human nuclei
CD4	Rat	Bio-Rad Antibodies, MCA1038GA	1:50	Helper T cells
CD8	Rat monoclonal IgG1	Bio-Rad Antibodies, MCA1039GA	1:50	Cytotoxic T cells
CD18	Mouse monoclonal IgG1	Leukocyte Antigen Laboratory, UC Davis, Sacramento, CA	1:50	Macrophages (blood/monocyte-derived)
CD20	Rabbit	Thermo Fisher scientific, PA5-16701	1:400	B cells
Iba1	Rabbit	FUJIFILM Wako Pure Chemical Corporation, 019-19741	1:500	Microglia
Rod opsin (Rho)	Mouse monoclonal IgG1	Millipore Sigma, MAB5316	1:200	Outer segment of rods
Arrestin 3 (human cone arrestin, hCA)	Goat	Novus Biologicals, NBP1-37003	1:300	Cones (human)
M/L opsin	Rabbit	Millipore Sigma, AB5405	1:200	Red/Green cones
S opsin	Rabbit	Millipore Sigma, AB5407	1:200	Blue cones
PCNA	Mouse monoclonal IgG2a	Santa Cruz Biotechnology, sc-56	1:50	Proliferating cells or cells undergoing DNA repair
Phospho-Histone H3	Rabbit	Cell Signalling Technology, 9701	1:50	Proliferating cells
ZO-1	Rabbit	Thermo Fisher scientific, 40-2200	1:100	Outer limiting membrane
Protein kinase C (PKCα)	Mouse monoclonal IgG2b	BD Bioscience, 610108	1:50	Rod bipolar cells
G Protein Goα	Mouse monoclonal IgG1	Millipore Sigma, MAB3073	1:500	ON (rod and cone) bipolar cells
hSynaptophysin	Mouse	Invitrogen, 14-6525-82	1:100	Photoreceptor presynaptic vesicles (human)
GFAP	Rabbit	DAKO, Z0334	1:300	Müller glia
hGFAP	Mouse monoclonal IgG1	(STEM123) Takara Bio, Y40420	1:500	Human Müller glia

SUPPLEMENTAL EXPERIMENTAL PROCEDURES

Supplemental Experimental Procedures S1.

Details of hESC-PRPCs preparation and subretinal delivery.

Variation between organoids is common, as described by the Gamm lab in their 2019 Development paper (Capowski et al). To mitigate organoid to organoid variability, ~100-200 organoids were pooled for each transplantation, and the same cell line was used for each differentiation.

Stage 2 retinal organoids (ROs) (day 104-151 of differentiation, see Capowksi et al 2019 for description of organoid stages) were shipped overnight from the University of Wisconsin to the University of Pennsylvania in transport medium (Hibernate™ CTS media with 2% FBS, 2% B27, 1% PSA) at 4ºC where they were immediately transferred to retinal differentiation medium (RDM+RA+2% FBS) and maintained at 37ºC in a 5% CO2 controlled incubator (Model SCO6AD, Shel Lab, Cornelius, OR) with media changes performed until processing for transplantation. Following several rounds of washes with Hank's Balanced Salt Solution (HBSS) without calcium and magnesium (HBSS -/-), ROs were diluted in BSS and processed by mechanical fragmentation or enzymatic papain digestion, producing PRPC-rich aggregated or dissociated cell suspensions, respectively (Table 1). Stage 2 ROs were dissociated to single cells with papain (Worthington Biochemical) at 37°C with periodic trituration until organoids were fully dissociated. Following enzymatic deactivation with ovomucoid, cells were passed through a 40 µm filter and resuspended in sterile HBSS -/- to reach the desired cell concentration. Cell viability of 90-99% was routinely obtained following papain dissociation, and cell suspension was maintained on ice until transplantation.

The surgical procedures in dogs were conducted under general anesthesia achieved by propofol induction (4 mg/kg, PropoFlo™ Zoetis, Kalamazoo, MI) and isoflurane maintenance (2–3%; Akorn, Inc. Lake Forest, IL).

<u>Subretinal bolus injection technqiue</u>: The cell suspension containing PRPCs was delivered in the SRS of 10 dogs (18 eyes), using a subretinal injector (RetinaJect, SurModics, CA) (Komaromy et al., 2006) that was custom-modified to replace the 39 gauge polyimide cannula with either a 31 or 33 gauge cannula. In brief, the needle of the subretinal injector was inserted into the vitreal cavity via a pars plana approach and visualized with a Machemer magnifying lens (OMVI; Ocular Instruments Inc., Bellevue, WA, USA) and a surgical microscope (Zeiss Universal S2B Operating Microscope, Oberkochen, Germany). Once the needle was in the posterior vitreous, the inner polyimide cannula (33 or 31 gauge) was extended to contact the retina at the desired location, at which point manual bolus injection of the cell suspension created a retinotomy and a subretinal bleb began to form. The surgical outcome was documented immediately post-injection with a fundus camera (RetCam Shuttle, Clarity Medical Systems, Inc. Pleasanton, CA, USA). Except for one of the normal dogs that had a bilateral vitrectomy performed two years prior to the enrollment in this study, all other animals underwent this subretinal injection without prior vitrectomy.

<u>5-step subretinal injection technique</u>: The entre surgical procedure was performed under direct visualization of the fundus with a digital operating microscope (Zeiss OPMI Lumera 700, Carl Zeiss Meditec AG, Jena, Germany) equipped with intraoperative OCT (Zeiss Rescan 700) and a 3D visualization system (Ngenuity®, Alcon, Fort Worth , TX). Following a complete 3 port pars plana vitrectomy (step 1) (Stellaris, Bausch & Lomb, 25 G instruments), after removal of the vehicle triamcinolone acetonide (Kenalog, 40 mg/mL, Bristol-Myers-Squibb) was resuspended in BSS and a 0.1-0.2 mL (4-8 mg) solution was injected into the vitreal cavity to help with visualization and removal of the posterior hyaloid membrane (step 2). A pneumatic-assisted subretinal injection of BSS (~ 100 µL) was performed using a 38 gauge cannula (PolyTip® cannula 25g/38g, MedOne, Sarasota, FL) with a syringe (MicroDose™ Injector, MedOne) connected to the VFI tubing set of the Stellaris system with a maximal pressure set at 16 PSI (step 3). Following formation of the subretinal bleb, the cannula was maintained in place and slow reaspiration (max aspiration pressure set at 600 mmHg) of ~ 50 µL of BSS was performed (step 4). Delivery of the cells into the same bleb trhough the intial or a second retinotomy site

was perfomed by using a 31 gauge cannula (PolyTip® cannula 25g/31g, MedOne) connected to another MicroDose™ Injector under pressure-controlled assistance (step 5). Intraoperative OCT was used to confirm the formation of the bleb and delivery of cells into the SRS. Video recordings of the surgery were used to document absence of any significant vitreal reflux after retraction of the subretinal cannula.

Supplemental Experimental Procedures S2.

Perioperative pharmacological treatment

On the morning of surgery, all the dogs received a single application of topical antibiotic (Gentamicine sulfate solution 0.3%, Allergan USA, Inc. Madison, NJ) and topical non-steroidal anti-inflammatory (Flubiprofen 0.03%, Bausch+Lomb, Tampa, FL). The animals from the no-IS (Fig. S1 B) group also received a single application of topical corticosteroids (Prednisolone acetate 1% suspension, Allergan, Irvine, CA) preoperatively. A triple topical mydriatic protocol was used in all the dogs before the surgical intervention, that included: tropicamide 1% (Akorn, Inc. Lake Forest, IL), atropine sulfate 1% solution (Akorn, Inc. Lake Forest, IL) and phenylephrine hydrochloride 10% (Paragon Bioteck, Portland, OR), with all three drugs given three times 30 minutes apart. Immediately after the surgery, topical application of antibiotic and steroid ointment (Neomycin Sulfate, Polymyxin B Sulfate and Dexamethasone, Bausch and Lomb, Bridgewater, NJ) was given together with atropine sulfate 1% ointment (Bausch+Lomb, Bridgewater, NJ). This atropine ointment was continued twice a day for the first week PI to all the dogs. Immediately after the surgical procedure, a subconjunctival injection of 4 mg of triamcinolone acetonide 40 mg/mL (Bristol-Myers Squibb, Montreal, Canada) was given to dogs in the IS group (Fig. S1 A); this was repeated at 4 weeks PI. A broad-spectrum oral antibiotic therapy was administered (Amoxicillin trihydrate/clavulanate potassium 12.5-20 mg/kg, twice a day, Dechra Veterinary Prodcuts, Overland Park, KS) during the first two weeks (no IS group) or five weeks (IS group). Prednisolone acetate 1% was also given postoperatively in the IS animals (Fig. S1 A), twice a day for 2 weeks and once a day for another 2 weeks.

Immunosuppression monitoring

Throughout the evaluation period, physical examinations, blood and urine collection were performed to assess if the IS protocol was well tolerated by the dogs and to ensure that proper IS levels were achieved. Complete blood count, biochemical analysis of hematologic and urine samples, and urine culture were carried out before injection and at 1-, 4-, 8-, 12-, and 20-weeks PI (data not shown). PK and PD assays were used to ensure that the CsA blood levels and the IS achieved were adequate (Fig. S1 C-D). CsA therapeutic range was considered optimal when the levels were between 800-1400 ng/ml, 2h post drug administration, or 400-600 ng/ml, before the next dose (Fig. S1 C). For PD evaluation, a molecular PCR-based assay of activated T-cell mRNA IL-2 expression was performed. We considered sufficient IS when the suppression of T-cells, measured indirectly by IL-2 mRNA inhibition, was within 50 to 100% to that of control dogs (moderate to marked IS), as recommended by the laboratory (Pharmacodynamic Laboratory, Mississippi State University, MS) (Fig. S1 D). These assays were carried out at weeks 1, 4, 8, 12, and 20 PI. Since no such analyses are developed for measuring MMF- or prednisolone-related IS in dogs, we evaluated the results of CsA PK/PD assays together with the clinical signs of transplant rejection in order to assess if modifications to the IS regimen were needed.

Supplemental Experimental Procedures S3.

In vivo quantification of the subretinal cell clusters over time

The initial time-point of measurement was one week PI, and subsequent measurements of the same region (using the follow-up tool of the Spectralis unit) at 4-, 8- and 12-weeks PI were compared to the initial value. Two types of measurements were combined to provide information of the transplant modifications: transplant area (mm2) and mean transplant height (µm). The transplant area was measured on the near infrared cSLO image, by delineating the region that co-located with the subretinal cell cluster on OCT to define the donor-host boundary. HEYEX software was used to outline the transplant and estimate the area (Fig. S5 A1). The mean transplant height was measured in all the animals by manually placing the caliper bar (HEYEX software) from the base of the transplant, perpendicular to the RPE, to the innermost detectable limit of the subretinal cells (Fig. S5 A2); this was repeated every 10 scans from the raster scan (every 1.2 mm). The percentage of the cell mass or height compared to that obtained at 1 week PI, was averaged for the animals within each group (normal IS, normal no IS, mutant IS and mutant no IS).

A final combined analysis of the graft volume was estimated. To this end, we assumed that the area and mean height of the transplant formed a geometrical cone shape. Then, we calculated the volume of the “grafted cone” by multiplying the cone area by 1/3 of the cone height. This volume was normalized to that found at 1 week PI and the results were sorted by group.

Supplemental Experimental Procedures S4.

Preparation of immunostained sections.

The eyes were fixed in 4% paraformaldehyde for 3 hours, followed by 2% paraformaldehyde for 24 hours, trimmed, cryoprotected in 15%–30% sucrose/PBS solution, and embedded in optimal cutting temperature media. Ten microns-thick cryosections were obtained (Microm HM550; Thermo Fisher Scientific, Waltham, MA) from the areas where the photoreceptor precursors were previously identified by OCT; the region was localized by landmarks of the retinal vascular pattern in comparison with infrared cSLO images and OCT. As a complementary analysis, archival retinal sections from three uninjected rcd1/*PDE6B* mutant animals of 22, 26 and 39 weeks with both genders represented, were studied by IHC. These were used to accurately interpret the histological findings in the hESC-PRPCs treated mutant dogs.

The sections were stained with different IHC markers (Table S2). Antigen retrieval was performed prior to immunolabelling against ZO-1, PCNA and PHH3 antibodies by two cycles of 125 ºC/1.5 min and 90 ºC/10s in the presence of Antigen Unmasking Solution (Vector Laboratories, Burlingame, CA) using a decloaking chamber (Biocare medical, Concord, CA). The antigen-antibody complexes were visualized with fluorochrome-labeled secondary antibodies (Alexa Fluor, 1:200; Molecular Probes), and Hoechst 33342 nuclear stain (Molecular Probes) was used to label cell nuclei.

Supplemental Experimental Procedures S5.

Immunohistochemical quantification of the integration events in a cohort of normal and mutant animals

The frequency of integration of the major cell cluster was investigated in a cohort of normal and mutant animals. We selected three eyes from the mutant-IS group and three eyes from normal-IS animals for further quantification of the frequency of integration events of the main donor cell cluster into the host retina. In each eye, we identified the main cell cluster located in the pseudohypopyon area based on fundus photography and selected 10 consecutive retinal cryosections, each section being 10 μm thick. In each slide a yes/no answer was recorded according to the presence or absence of migration of the major cell cluster into the host retina.

SUPPLEMENTAL REFERENCES

Capowski, E.E., Samimi, K., Mayerl, S.J., Phillips, M.J., Pinilla, I., Howden, S.E., Saha, J., Jansen, A.D., Edwards, K.L., Jager, L.D., et al. (2019). Reproducibility and staging of 3D human retinal organoids across multiple pluripotent stem cell lines. Development (Cambridge, England) 146. 10.1242/dev.171686.

Komaromy, A.M., Varner, S.E., de Juan, E., Acland, G.M., and Aguirre, G.D. (2006). Application of a new subretinal injection device in the dog. Cell Transplant 15, 511-519. 10.3727/000000006783981701.

Supplemental Video S1. 3D confocal imaging of donor human PRPCs integrated into a normal canine retina and adopting a cone-like morphology. Two tdTomato-positive PRPCs expressing the human nuclear antigen (Ku80) have migrated their cell bodies into the host's ONL. These cells show an elongated photoreceptor-like morphology that includes an inner segment, and an axon extending towards the host's outer plexiform layer and ending with a pedicle-like structure. Images are shown with and without differential interference contrast (DIC)/Nomarski optics.

Supplemental Video S2. 5 step surgical procedure used to optimize in the canine eye the subretinal delivery of cell suspensions by avoiding reflux into the vitreal cavity.
